# Bleating, growling, barking, and spitting: Metaphorical extensions and valency patterns of verbs of speaking

**DOI:** 10.1371/journal.pone.0325807

**Published:** 2025-06-10

**Authors:** Ivana Brač, Kristina Š. Despot, Branimir Belaj

**Affiliations:** 1 Institute for the Croatian Language, Zagreb, Croatia; 2 Faculty of Humanities and Social Sciences, Josip Juraj Strossmayer University of Osijek, Osijek, Croatia; Dr NGP Institute of Technology, INDIA

## Abstract

This corpus-based and qualitative study examines the valency patterns of Croatian verbs that encode verbal activity and belong to the semantic field of verbs of speaking through metaphorical and metonymic extensions, using a cognitive linguistics framework, specifically the usage-based model. The analysis focuses on examples such as the metaphoric use of animal sounds (e.g., *blejati* ‘to bleat’) and verbs associated with bodily processes (e.g., *srati* ‘to shit’), which often convey negative or stereotypical attitudes towards speakers or messages, or even extreme disdain. This paper contributes to the understanding of cross-domain figurative extensions of verb meanings and their valency adaptations. A dataset of 438 example sentences containing 152 verbs with figurative extensions targeting the domain of speaking was meticulously compiled from Croatian corpora. This dataset enabled a manual annotation and analysis of the transfer and adaptation of valency patterns across domains. The study addresses the following key questions: 1. What source domains are used for verbs of speaking as targets? 2. Do verbs retain the valency patterns of the source domain or adopt those of the target domain? 3. Is the passivization of transitive verbs possible in metaphorical contexts? The findings indicate that with a metaphorical shift in meaning, verbs often adopt new valency patterns from the target domain. Our examples of valency pattern change as a result of a metaphorical meaning shift demonstrate that verbs can appear with arguments not explicitly subcategorized by the verb itself.

## 1. Introduction

Verbs of speaking (VoS) are among the most frequent yet highly expressive linguistic elements, often used to encode nuanced attitudes, evaluations, and interpersonal dynamics. They play a distinctive role in describing cognitive processes, as interlocutors frequently use them to convey facts, ideas, and emotions, making them an intriguing research topic for linguists of various backgrounds, particularly cognitive linguists.

It has been noted that Slavic languages employ a greater variety of verbs of speaking than languages such as English [1,2], and this observation holds true for Croatian as well [3–5]. While general verbs of speaking (e.g., *say, tell, speak*) are among the most frequent in Croatian, verbs from various semantic domains are often used figuratively as verbs of speaking to express particular attitudes toward the interlocutor or the communicative situation. This phenomenon arises from metaphorical or metonymic shifts in verb meanings. By selecting a verb from a specific domain rather than a prototypical verb of speaking, speakers incorporate their subjective evaluation of the situation being described. For instance, verbs associated with animal sounds are used as verbs of speaking, creating metaphorical mappings from the animal to the human world, often with the intention of describing the interlocutor’s voice as unpleasant or unintelligent (e.g., *blejati* ‘bleat’). This usage can extend further down the “Great Chain of Being” [6], as seen with verbs of sound emission. For example, *otkucati* in *Sat je otkucao ponoć* (‘The clock struck midnight’) is metaphorically extended to a human agent in *Otkucao ga je policiji* (‘He ratted him out to the police’). Verbs associated with bodily processes, such as *srati* (‘shit’) or *pljuvati* (‘spit’), are used to express an extremely negative attitude toward the interlocutor’s words.

The figurative use of these verbs frequently highlights certain aspects of the act of speaking [7,8]. Consequently, these verbs are typically classified as verbs of manner of speaking [4,9–11]. However, they are often regarded as non-prototypical members of a wider category of verbs of speaking, which can be broadly defined as any verb semantically related to the action of speaking [12].

In this study, we examine Croatian verbs that, while not originally verbs of speaking in their denotative meanings, have become verbs of speaking through metaphorically and metonymically motivated meaning shifts. Our aim is to conduct a corpus-based and qualitative investigation into the mechanisms underlying these meaning transfers and their impact on valency patterns.

The analysis is conducted within the framework of cognitive linguistics, specifically the usage-based model [13–18], conceptual metaphor theory [7,19–21], and constructional approaches to grammar, primarily Cognitive Grammar [13,16,22,23] and, to a lesser extent, Construction Grammar [24] (for more details on theoretical background see Ch. 2).

Our study employs a relatively novel approach (for details on methods see Ch. 3) by using corpus data to explore the relationship between the metaphoric usage of verbs and their valency patterns (Ch. 4) – a topic that has, to date, received little to no attention in linguistic research. The answers to our research questions and the conclusions about this relationship are presented in Ch. 5.

## 2. Theoretical background

### 2.1. Previous work

Verbs of manner of speaking, like prototypical verbs of speaking, denote the transfer of a message through speech. However, they place emphasis on aspects such as volume, intensity, speed, the speaker’s attitude or intention, the effect on the hearer, directionality, persistence, and other features [10,11]. Emotional, judgmental, and evaluative components are also consistently present [4].

Their distinctive semantic properties, as noted above, and their syntactic peculiarities – such as extraction, complementizer omission, double object constructions, and more – have been extensively studied in relation to verbs of speaking [25–32]. These findings support the thesis that verbs of manner of speaking constitute a distinct semantic class [9,33].

In [3], English verbs that do not primarily belong to the category of verbs of speaking but can be used as such due to metonymic (verbs of breathing, verbs of laughing, verbs of crying, verbs of spitting, and other verbs of physiological processes) or metaphorical extensions (verbs of sounds made by animals, verbs of sound emission, verbs of movement and force) are detected. Verbs of breathing and verbs of sounds made by animals are analyzed in more detail, explaining their syntactic behavior. However, [3] does not analyze other English classes or any Croatian verb that have undergone metonymic or metaphorical extensions.

In [4], Croatian verbs of manner of speaking are analyzed, with the focus on the semantic differences between prototypical verbs of speaking and verbs of manner of speaking and their impact on valency patterns, as well as the differences between different classes of verbs of manner of speaking. This analysis reveals that, contrary to earlier claims, these verbs in Croatian are not exclusively monovalent but can also exhibit bivalent and even trivalent valency patterns. This analysis also includes verbs that primarily belong to different semantic domains. More precisely, the class of verbs of meaningless speaking and verbs of complaining, which include verbs that primarily belong to the classes of verbs of sounds made by animals and sound emission, and verbs with an emphasis on an emotional component, which includes breathe verbs or psych-verbs, verbs of sounds made by animals, and sound emission. However, the mechanisms of meaning extension are not analyzed, nor is there an attempt to answer the research questions posed in this study. In [34], the ideological consequences of various interpretations of the metaphor HUMANS ARE ANIMALS are examined. It contrasts perspectives that view humans as complex animals in a literal sense with those that understand the statement as a metaphor. Sociobiological theories, reinforced by metaphors found in English dictionaries, highlight competition and aggression as traits common to both humans and nonhuman animals. In contrast, other perspectives emphasize cooperation and symbiosis. Some of these theories are normative, as metaphorical patterns in English often frame animal behavior as something humans should avoid. On the other hand, sociobiologists argue that behaving like animals is natural and justified, reflecting the naturalistic fallacy. Meanwhile, certain cultural theories propose that the statement is purely metaphorical, emphasizing human uniqueness as our defining trait. This work demonstrates that with a target as complex and multi-faceted as ‘animal’ there is enormous scope for different source metaphors derived from different concepts of humanity and different social and cultural systems within which that humanity takes its shape. This study offers a valuable theoretical framework for understanding the negative emotional valence associated with certain metaphorically extended verbs of animal sound production (see Ch. 4.2.2). However, it does not address the structural and syntactic implications of metaphorical verb usage, which form the central focus of our research.

Metaphorical mapping from the domain of the animal world to the human world in the Russian language, with reference to other languages, is studied in [35,36], where inarticulate sounds made by humans are classified into 1. non-verbal and 2. verbal sounds. Verbal sounds are further divided into 1. disapproving reactions (weak and aggressive confrontation), 2. approving reactions, 3. plural subject, and 4. semiotically significant speech. In [35,36], the focus is on the construction of a typology of verbs denoting animal sounds and addressing the question of sound categorization across different languages. As previously stated, the study covers only verbs of animal sounds, whereas our study includes verbs of sound emission, bodily processes, crying, throwing, breathing, and deconstruction. It does not examine the syntax-semantic interface.

In [5], the focus is on verbs that use the metaphor of animal sounds to describe human speech (e.g., *beknuti*_PF_ ‘bleat’, *blejati*_IMPF_ ‘bleat’, *cvrkutati*_IMPF_ ‘chirp’, *kreketati*_IMPF_ ‘croak’, *kokodakati*_IMPF_ ‘cluck’, *lajati*_IMPF_ ‘bark’, *kriještati*_IMPF_ ‘tsquawk’, etc.). This research reveals that these metaphoric verbs carry a strong evaluative component, often characterizing speech as boring, empty, rapid, repetitive, tiresome, overly persistent (in a negative sense), excessively sweet or flattering (used manipulatively), or banal. It is shown that these verbs are frequently employed for highly negative evaluations and ironic commentary about speech, including doubts regarding the truthfulness of the content, the accuracy of the speaker’s information transfer, or the rationality of their conclusions. Additionally, the study explores the prominence of this negative evaluation and irony in relation to gender. This study is primarily pragmatic and does not deal with valency patterns and meaning transfers.

In [37], the effectiveness of semantic generalizations and classifications in capturing the regularities of verb behavior concerning their metaphoricity is examined. Beginning with orthographic word unigrams, the study explores various methods for defining semantic classes for verbs – grammatical, resource-based, and distributional. The effectiveness of these semantic classes is then evaluated in classifying all verbs within a running text as either metaphorical or non-metaphorical. This resource-based classification is based on the VerbNet database [38], which provides a classification of verbs according to their participation in frames – syntactic patterns with semantic components, based on Levin’s classes [9].

While our study builds on this previous research regarding different implications of the HUMANS ARE ANIMALS metaphor, our study takes a different approach by focusing on the structural and syntactic implications of metaphorical verb use. Specifically, we examine how metaphorically extended verbs undergo valency pattern changes, a phenomenon that has not been systematically explored in previous research. Whereas prior work has primarily addressed how metaphorical projections reflect societal values and ideological structures, our analysis delves into the linguistic mechanisms that facilitate these metaphorical shifts, particularly in terms of argument structure and syntactic adaptation. By investigating how verbs from various source domains (e.g., animal sounds, physiological processes) acquire new valency patterns when used as verbs of speaking, our study offers a novel contribution to both cognitive linguistics and metaphor research, expanding the discussion beyond conceptual framing to encompass the morphosyntactic consequences of metaphorical extension.

### 2.2. Current study

In this paper, we focus on verbs of manner of speaking that have undergone metaphorical meaning shifts. Our objective is to explore the mechanisms behind these meaning shifts and their impact on valency patterns by addressing the following research questions:

1. What source domains are used for verbs (of manner) of speaking as targets, and what might speakers aim to achieve by employing figurative uses of verbs from other domains instead of prototypical verbs of speaking?
2. Do verbs retain their valency patterns from the source domain, or do they adopt new patterns characteristic of the target domain?
3. Is the passivization of transitive verbs possible in metaphorical contexts?

Through this investigation, we aim to provide a deeper understanding of the interaction between metaphorical meaning shifts and syntactic valency adaptations.

This study is grounded in conceptual metaphor theory [7,19,39] and the theory of primary metaphors [20,40], using a widely recognized and thoroughly tested metaphor identification method (MIP) [41]. Conceptual metaphor theory (CMT) underscores the central role of metaphors in everyday language use and positions them as a fundamental component of our conceptual system. This perspective posits that metaphors are integral to the encoding, storage, representation, and retrieval of concepts, with the activation of metaphorical structures being an inherent part of conceptual thought. This view has gained broad acceptance across disciplines such as linguistics, literature, psychology, cognitive science, sociology, and neuroscience. According to CMT, abstract concepts are predominantly understood through metaphorical frameworks. Primary metaphors are often rooted in direct perceptual experiences [40,42]. As a result, abstract notions such as love, friendship, and morality are typically conceptualized in metaphorical terms, often linked to more concrete experiences like warmth, closeness, or cleanliness. A growing body of experimental evidence supports the cognitive connection between the source and target domains of metaphors, particularly in the case of primary metaphors [21,43,44]. We employ the notions of metaphorical *highlighting* and *hiding* [7,8]. Due to the inherently partial nature of metaphorical mappings, applying a source frame to a target selectively emphasizes specific aspects of the target (highlighting) while downplaying others (hiding). As a result, conceptual metaphors bring certain elements of the target frame into focus (i. e., profiling them), while obscuring those aspects that do not align with the metaphor [45].

Like metaphor, or perhaps even to a greater extent, metonymy has proven to be pervasive across cognition, grammatical meaning and form, pragmatic inferences and discourse, language change, and extralinguistic domains such as art and gesture (for an overview of the role of metonymy in all these areas, see [46]. Metonymy, as a mapping of one role onto another within the same semantic frame, in contrast to metaphor, which maps roles from one semantic frame onto those of another, also has a conceptual nature [7]. In [47], a detailed analysis of further distinctions between metaphor and metonymy is provided, some of which are particularly relevant to our discussion. Unlike metaphor, where the source domain is often more concrete than the typically abstract target domain, this is not necessarily the case in metonymy. Conceptual metonymy involves entire domains and their constituent parts, meaning that differences in concreteness or abstractness are not as pronounced. As a result, metonymic mappings can sometimes be reversed, making metonymy, unlike metaphor, inherently reversible [48]. The relationship between metaphor and metonymy is often complex and multilayered, and has been the subject of extensive research in cognitive linguistics. For instance, in [49], it is argued that metonymy underlies all metaphors, whereas in [8], only correlation-based (primary) metaphors are seen as metonymically grounded. In [50], this interaction is explored in depth and the concept of *metaphtonymy* is introduced to describe the interplay between conceptual metaphor and metonymy.

While verbs of speaking have been extensively studied (see previous chapter) across various languages and theoretical frameworks, including their syntactic and semantic characteristics, even within Croatian, the relationship between metaphorical mappings and verb valency pattern inheritance remains an underexplored area of research. This corpus-based study on the Croatian language aims to address this gap, offering insights that may serve as a foundation for investigating whether valency pattern inheritance in metaphorical mappings (from the source domain or the target domain) is a universal phenomenon or subject to cross-linguistic variation.

## 3. Methods: Materials and procedure

For this study, we compiled a list of 152 figurative uses of verbs from various source domains that are used as verbs of speaking. The list of 122 verbs was taken from [4]. This list was then extended by adding aspectual counterparts and other related verbs found in literature and dictionaries. In this study, from that initial list, we excluded verbs that had not undergone metaphorical extension, i.e., whose meaning is not marked as figurative in dictionaries. These verbs are mainly verbs from the subclass of verbs with an emphasis on the volume, such as *vikati* ‘shout, yell’, *vrištati* ‘scream’, and verbs of incomprehensible speaking, such as *frfljati* ‘gibber’, *mrmljati* ‘mutter’, *mucati* ‘stutter’. This resulted in a list of 104 verbs, which was then extended to 152 verbs by adding synonyms and verbs from the same semantic domains found in kontekst.io, a portal containing synonyms in Croatian, and in examples from the corpora. All verbs were attested in several Croatian corpora, mainly the *Croatian Web Corpus* – hrWaC [51], *Hrvatska jezična riznica* [52], and the *Croatian National Corpus* – HNK [53] available in Sketch Engine [54] since many of the analysed verbs are not frequent.

For each verb, an example from the corpora was provided, with non-relevant elements of the sentence removed to focus on aspects pertinent to the research. If a verb exhibited only one valency pattern, at least one example was included. For verbs with multiple valency patterns, examples were provided for each pattern, resulting in a total of 438 examples. In addition to providing examples, the corpora were used to confirm the verb’s usage as a verb of speaking, identify its valency patterns, and determine the contexts in which it is employed. All examples in this paper are from the corpora.

For more frequent verbs, we selected a representative sample of 2,000 sentences using random sampling and then manually reviewed their meanings and valency patterns. For less frequent verbs, a comprehensive manual review was conducted by analyzing all available examples to determine their meanings and valency pattern. For more frequent verbs, in addition to manual review, we utilized CQL (Corpus Query Language) queries to identify more suitable examples for specific valency patterns. It is important to note that while other valency patterns may exist, our research was based exclusively on corpus data, and any frames not present in the corpora were not included in this study. All examples were manually annotated by two annotators (Author1 and Author2) who evaluated each example to determine whether it involved metaphorical or metonymic meaning shift, identified the corresponding source domain, and annotated the valency patterns for each instance using a widely recognized and thoroughly tested metaphor identification method (MIP) [41], which includes: 1) reading the example in the context; 2) identifying potentially metaphorical lexical units; 3) (a) determining the contextual meaning of the potentially metaphorical lexical unit, (b) identifying the basic meaning of the potentially metaphorical lexical unit, (c) comparing the contextual and basic meanings of the potentially metaphorical lexical unit; 4) identifying metaphorical lexical units. Determining basic meaning follows the criteria established by the Pragglejaz Group [41], which defines basic meaning as one that is: more concrete, directly linked to the sensory domain, related to bodily functions (sensorimotor-motivated), cognitively more structured, and historically older. In determining the meanings of lexical units, we also relied on available historical and contemporary dictionaries of the Croatian language, as well as online lexicographic resources. For the difference between metaphor and metonymy, see Ch. 2.2.

The annotation of valency patterns included several levels for each argument: (a) the syntactic phrase type (e.g., quotation, NP, PP), (b) its morphological realization (e.g., prepositions and cases, which are morphologically marked in Croatian), and (c) its semantic role (partially adapted from VerbNet’s semantic roles. For the full list of roles used, see Appendix 1 in S1 File).

In addition, valency patterns were defined for each verb in its primary sense, as well as for four prototypical verbs of speaking (*govoriti* ‘speak,’ *pričati* ‘talk,’ *reći* ‘say,’ *kazati* ‘tell’). After calculating the inter-annotator agreement, any discrepancies were reviewed and resolved in collaboration with a third researcher (Author3) to reach a consensus. The subsequent step involved comparing the valency frames of the 152 figurative uses of verbs with their primary (non-figurative) valency patterns and those of the prototypical verbs of speaking. This comparison aimed to address the second research question regarding whether the valency patterns of these verbs are inherited from the source domain or adapted to the target domain.

Regarding determining valency patterns, the annotation agreement was 90.64%. The main disagreement involved the assignment of semantic role to the third participant, i.e., the PP *na* ‘at’ + accusative, as in (19), and the NP in the accusative as in (51), which is explained in detail in Ch. 4.2.2 and 4.2.5.

## 4. The analyses

### 4.1. Figurative mappings and source domain analyses for the VoS target domain

**Fig 1:**
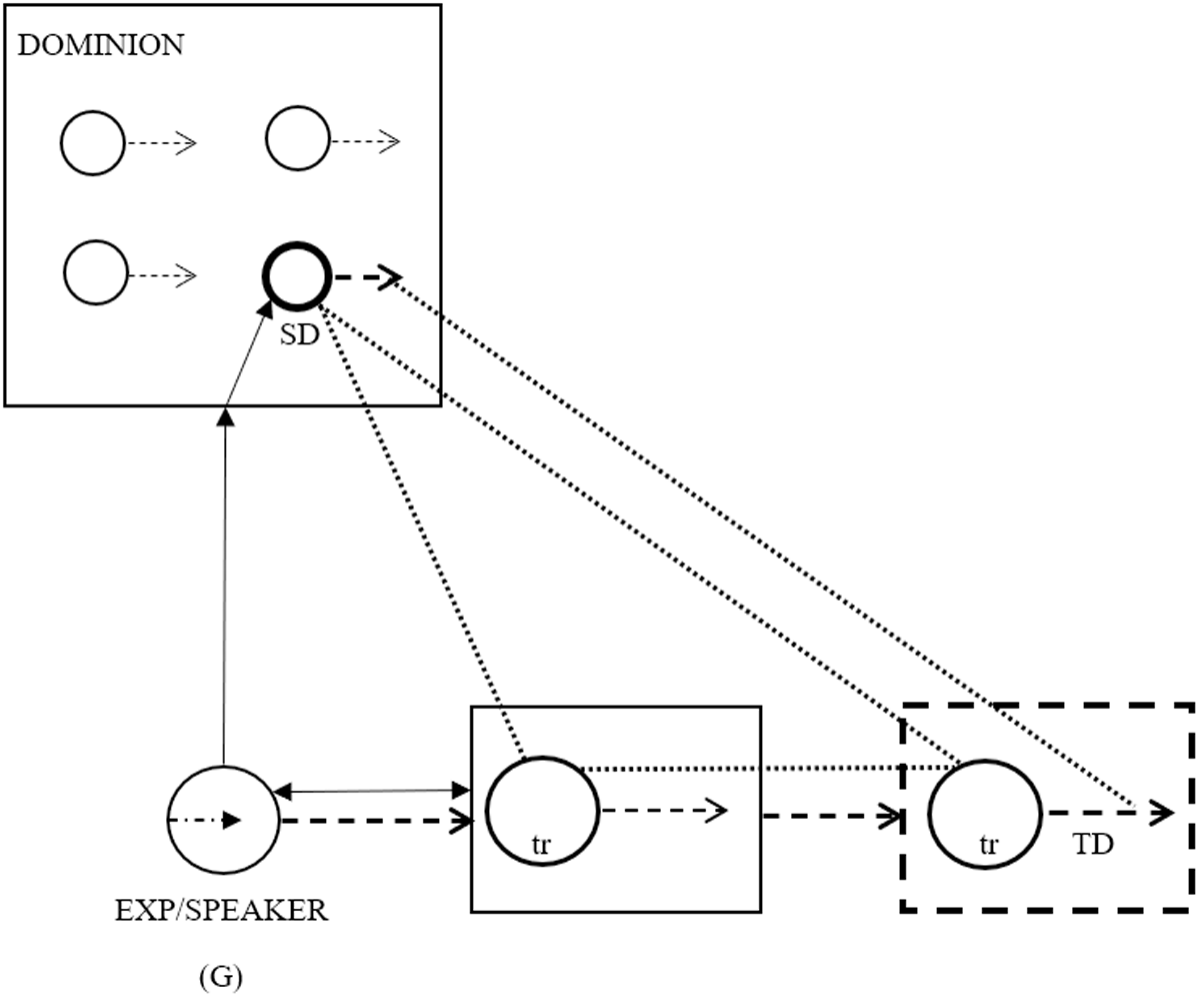
Metaphorical mapping of various aspects of sound or vocalization onto human speech.

Fig 1 illustrates the general metaphorical mapping of various aspects of sound or vocalization onto human speech. The Figure represents a Speaker, who is an experiencer entity belonging to the Ground. The Ground itself anchors the speech act and its participants into space and time. When the Speaker comes into perceptual, i.e., auditory interaction (double-headed arrow) with another person whose manner of expression reminds him of the sound, action or vocalization of an animal, in his mind a conceptual dominion is called up consisting of a range of sounds or actions of that type, from which the Speaker selects (solid-line arrow) the most fitting one as the source domain for metaphorical mapping (bolded circle). Broken-line arrows represent the more abstract action of speaking on the part of both Speaker and the person referred to by the utterance; dotted lines signal correspondences between source and target domain entities and the broken-line rectangle represents the metaphorically expressed target domain.

As the first step of the analysis, we categorized all verbs from our dataset according to their source domain (see Table 1). The verbs were classified manually by two annotators, starting with the classification adapted from [9] with a few modifications. For instance, verbs of crying and laughing are treated as two distinct categories rather than being grouped together under the broader class of verbs of nonverbal expression. The breathe verbs and exhale verbs, which are subclasses of the class verbs involving the body and the subclass verbs of bodily processes, were merged into a single category and separated from the class of verbs of bodily processes, which in our study comprises verbs denoting bodily elimination (e.g., *shit*, *poop*, *spit*). The class of verbs of deconstruction includes verbs from the classes of verbs of cutting – such as cut verbs (e.g., *saw*), carve verbs (e.g., *grind*, *crush*) – and verbs of separating and disassembling, such as split verbs (e.g., *saw*) [9]. Additionally, verbs of sound emission and weather verbs are merged into a single category, as only one aspectual counterpart is included in the research (*grmjeti*_IMPF_/*zagrmjeti*_PF_ ‘thunder’). In this paper, we analyze verbs belonging to classes with five or more members.

**Table 1:** Distribution of verbs by source domain.

	Source domain	Number	Percentage
1.	Sounds made by animals	75	49.34%
2.	Sound emission	31	20.39%
	Weather verbs	2	1.32%
3.	Bodily processes	12	7.89%
4.	Crying (psych-verbs)	10	6.58%
5.	Throwing	6	3.95%
6.	Breathing	5	3.29%
7.	Deconstruction	5	3.29%
8.	Laughing	2	1.32%
9.	Coloring	2	1.32%
10.	Singing	1	0.66%
11.	Social interaction	1	0.66%
		Σ 152	

As can be seen in Table 1, the largest class consists of verbs from the animal world domain.

### 4.2. Analysis of the change in valency patterns caused by metaphoric meaning extension

In this chapter, we provide a comprehensive analysis of the valency patterns of all verbs included in our dataset. In Section 4.2.1, we examine the prototypical verbs of speaking, focusing on their possible valency patterns. In the subsequent sections, we analyze the valency patterns of metaphorical verbs of speaking (comparing them to valency patterns of the same verbs in their literal meanings), organized according to their respective source domains.

#### 4.2.1. Verbs of speaking.

According to the Wordlist in several Croatian corpora (e.g., hrWac, Riznica, MaCoCu), the most frequent verbs of speaking in Croatian are *reći* ‘say, tell’, *govoriti* ‘speak, talk’, *kazati* ‘say, tell’, and *pričati* ‘talk’. With all of them, the theme can be expressed by quotation (QUOT) (1), clausal complement (2), noun phrase (NP) in the accusative (3a), prepositional phrase (PP) *za* + accusative (4a), but they differ regarding the PPs *o* ‘about’ + locative (5), *protiv* ‘against’ + genitive (6), and *na* + accusative (9a).

(1) *Sabina je rekla Zorki: - Čudan je tvoj otac!*

‘Sabina told Zorka: - Your father is strange!

(2) *Govoriš ljudima da su najgenijalnija bića na ovom svijetu.*

‘You tell people that they are the most brilliant beings on this planet.’

The theme in the accusative always denotes narrative-related expressions (*gluposti* ‘nonsense’, *istina* ‘truth’, *laži* ‘lies’, *priča* ‘story’, etc.) (3a) [4] or indefinite pronouns (*nešto* ‘something’, *ništa* ‘nothing’, *svašta* ‘everything’). Since it concerns the act of speaking, the result of the action is various verbal expressions that represent an effected object, which necessarily appears in the accusative case. The result, consequence, or product of the action is closely related to the (achieved) goal of the action as a schematic accusative meaning [55].

(3a) *Premijer govori gluposti.*

‘The prime minister is talking nonsense.’

Expressions that are not narrative-related cannot be expressed by the accusative (3b) but by the PP *o* ‘about’ + locative (3c).

(3b) *Premijer govori vrijeme.

‘*The prime minister is talking the weather.’

(3c) *Premijer govori o vremenu.*

‘The prime minister is talking about the weather.’

The theme expressed by the prepositional phrase *za* ‘for, about’ + accusative needs contextualization by clausal complement (4a) or NP in the accusative (4b).

(4a) *…ti si govorio za Arhelaja da je sretan.*

‘…you said for Archelaus that he was happy. = You said Archelaus was happy.’

(4b) *…ti si govorio za Arhelaja to.*

‘…you said that for Archelaus. = …you sad that about Archelaus.’

(4b) *…ti si govorio za Arheleja.

‘*…you said for Archelaus.’

The imperfective verbs *govoriti* ‘speak’ and *pričati* ‘talk’, and the perfective verbs *reći* ‘say’ and *kazati* ‘tell’ differ regarding the PPs *o* ‘about’ + locative and *protiv* ‘against’ + genitive. With the imperfective verbs *govoriti* ‘speak’ and *pričati* ‘talk’, those PPs can be used without any restriction (5–6), while with the perfective verbs *reći* ‘say’ and *kazati* ‘tell’, the PP cannot be used without an NP in the accusative (7b) or a clausal complement (8b) [4,56] since they are a resultative.

(5) *Najradije je govorio o svom zavičaju*.

‘He liked talking about his hometown the most.’

(6) *Robi govori protiv EU projekata*.

‘Robi speaks against EU projects.’

(7a) *Nikad nisam ništa rekao protiv suca.*

‘I’ve never said anything against the judge.’

(7b) *Nikad nisam rekao protiv suca.

‘*I’ve never said against the judge.’

(8a) *…o njemu su svi rekli što misle i što ne misle.*

‘…everyone has said about him what they think and what they don’t think.’

(8b) *…o njemu su svi rekli.

‘*…everyone has said about him.’

On the other hand, the PP *na* ‘on’ + accusative cannot be used with the imperfective verbs *govoriti* ‘speak’ and *pričati* ‘talk’ in any case (see details in Ch. 5), while with the verbs *reći* ‘say’ and *kazati* ‘tell’, contextualization by NP in the accusative or a clausal complement is necessary (9).

(9a) *…grad Split ni riječ ne reče na gubitak takvog znanstvenika.*

‘…the city of Split did not say a word about the loss of such a scientist.’

(9b) *grad Split ne reče na gubitak takvog znanstvenika.

‘…the city of Split did not say about the loss of such a scientist.’

Regarding the recipient, with general verbs of speaking, it can be expressed by NP in the dative (1). All general verbs of speaking can be used in the sense ‘give a statement to the media’, when the prepositional phrase *za* ‘for’ + accusative is used (10), or in the sense ‘to orally transfer a message in front of someone or a group of the people’ when the prepositional phrase *pred* ‘in front of’ + instrumental is used (11).

(10) *O tome i o aktualnoj gospodarsko-socijalnoj situaciji Kunst govori za Vjesnik*.

‘About that and the current economic and social situation Kunst talks for Vjesnik.’

(11) *Prije glasovanja Berlusconi će govoriti pred zastupnicima*.

‘Before the vote, Berlusconi will speak in front of the deputies.’

The construction *govoriti za Vjesnik* ‘talk for Vjesnik’ can be interpreted as a semantic extension motivated by one of the most common meanings of the PP *za* ‘for’ + accusative – namely, the meaning of purpose. In this case, the speech content is intended for publication in *Vjesnik*. While this represents a peripheral or extended meaning of purpose, the connection remains evident. Furthermore, this construction involves referential metonymy, specifically NEWSPAPER FOR THE PEOPLE WHO WORK IN IT. Similarly, in (11), the domain of message transfer inherently includes the spatial prelocation of the speaker, which can consequently stand for the act of conveying the message to listeners. This metonymic process can be defined as SPATIAL FRAME (SETTING) FOR ARGUMENT/PARTICIPANT/RECIPIENT/OBJECT COMPLEMENT, as seen in the case where *govoriti pred zastupnicima* ‘speak in front of representatives’ effectively means *govoriti zastupnicima* ‘speak to representatives’. A more concise formulation of this metonymy would be SETTING FOR RECIPIENT.

#### 4.2.2. Verbs of sounds made by animals.

Verbs associated with the sounds made by animals, when used in their literal meaning, are typically linked to the specific sounds produced by particular animals or groups of animals. For instance, a chicken can cluck, a frog croak, and a bird screech. In such literal instances, these verbs are predominantly intransitive, with a specific animal occupying the subject position (12).

(12) *Jata galebova letjela su i kričala od zore do mraka*.

‘Flocks of seagulls flew and screeched from dawn to dusk.’

These verbs often undergo a metaphorical meaning shift through the metaphor HUMAN SPEECH IS AN ANIMAL SOUND (an entailment of a more general metaphor PEOPLE ARE ANIMALS). PEOPLE ARE ANIMALS metaphor is an instantiation of the GREAT CHAIN METAPHOR [7], which provides a framework for understanding nonhuman attributes through the lens of human character traits through hierarchy *human beings > animals > plants > complex objects > natural physical things*. Metaphors based on the Great Chain consistently represent cases of one-correspondence metaphors [57]. In such metaphors, a single characteristic of the source domain is isolated and mapped onto the target domain. For example, in *Mary is a snake*, the malice and spite commonly associated with snakes (particularly adders) are used to characterize Mary’s behavior [58]. Through the PEOPLE ARE ANIMALS metaphor, we can interpret human attributes in terms of corresponding animal characteristics, and since it is based on the Great Chain metaphor, it is one-correspondence metaphor that highlights one specific aspect such as the manner of speaking (e.g., voice quality) or the speaker’s psychophysical state (13). For instance, when a human is described as barking, it means they are shouting in a loud, rough voice or speaking in a curt, loud, and typically angry tone [58].

(13) *Voditeljica krešti, griješi, uzdiše ko da se ona penjala*…

‘The hostess screeches, makes mistakes, sighs as if she were climbing…’

When used metaphorically, verbs of sounds made by animals can become transitive, or even ditransitive (see Table 2). As with verbs of speaking, the theme can be expressed by a quotation (14), a clausal complement (15), an NP in the accusative (16), or the PPs *o* ‘about’ + locative (17), *protiv* ‘against’ + genitive (18), while the PP *na* ‘for, about’ + accusative was attested only in one example with the verb *kokodaknuti* (20). It is interesting that with the PPs *o* ‘about’ + locative and *protiv* ‘against’ + genitive, contextualization is not needed, even with perfective verbs of sounds made by animals (*MMF-ovac, neki Grk, odmah je histerično zakriještao protiv toga.* ‘An IMF guy, some Greek, immediately screeched hysterically against it.’). This represents a difference in the valency pattern compared to the non-metaphoric perfective verbs *reći* and *kazati* ‘say, tell’ (cf. 7b, 8b). This occurs because verbs primarily belonging to the class of verbs of sounds made by animals do not require a direct object, even when used in their denotative meaning (*Na krovu stare vojarne zakriještao kapetanov paun.* ‘On the roof of the old barracks, the captain’s peacock screeched.’).

**Table 2:** Valency patterns of verbs of sounds made by animals used as VoS.

Theme	Recipient	Example
Quotation	NP in DAT	(14) *Uzalud je Nuša gukala Matanu: “Što će ti spišulje, sunce moje?”* ‘Nuša cooed to Matan in vain: “Why do you need those little women, my dear?”’
Clausal complement		(15) *Skupit će se šačica ljudi kojima je ‘pomoglo’ i kvakat će okolo da stvar funkcionira.* ‘A handful of people for whom it “helped” will gather together and quack= drone that the thing works.’
NP in ACC		(16) *A danas imamo mnoštvo partija a sve kokodaču istu priču.* ‘And today we have a multitude of parties, all clucking the same story.’
PP *o* ‘about’ + LOC		(17) *Ona kreštava crvenokosa političakra više ne krešti o pravima žena* ‘The shrill, red-haired female politician no longer caws about women’s rights.’
PP *protiv* ‘against’ + GEN	NP in DAT	(18) *Znam ja, tko tebi laje protiv mene!* ‘I know who is barking against me (to you)!’
PP *za* ‘for’ + ACC Beneficiary	PP *na* ‘at’ + ACC Patient/Experiencer/Stimulus	(19) *Joj kad se sjetim kak ste režali za Šukija, a sad režite na njega.* ‘Oh, I remember how you used to all howl for Šuki, and now you’re howling at him.’
PP *na* ‘on, about’ + ACC		NA (20) *Što vi možete kokodaknut na ovu temu?* ‘What can you cluck about on this topic?’

The PP *za* ‘for’ + accusative in (4a) and (19) semantically differs. In (4a), it denotes the theme, while in (19), the PP denotes the participant in whose favor one wants to achieve something, i.e., the beneficiary.

Regarding the recipient, it can be expressed by the dative (14, 18) and *na* ‘at’ + accusative (19), which is not attested with the prototypical verbs of speaking. It is interesting that it is attested with the verbs of loud speaking, such as *vikati* ‘shout, yell’ and it denotes anger or other negative emotions directed at the recipient. The verbs used to express the anger of animals that we perceive as dangerous (e.g., *horse*, *dog*, *bear*, *snake*) have that argument in the source domain (*frktati*_IMPF_/*frknuti*_PF_ ‘snort’, *režati*_IMPF_*/zarežati*_PF_ ‘growl’, *štektati*_IMPF_*/zašetaktati*_PF_ ‘yap’, *lajati*_IMPF_/*zalajati*_PF_ ‘bark’, *brundati* ‘grumble’, *siktati*_IMPF_*/siknuti*_PF_ ‘hiss’, etc.), but it is also attested with smaller and less frightening animals, such as birds (*graknuti* ‘caw’, *kokodakati* ‘cluck’, *kriještati* ‘screech’, *kričati* ‘screech’, etc.). This PP is attested with some psych-verbs that include the emotion of anger, such as *ljutiti se* ‘be mad, angry’ (21). In these cases, the preposition *na* ‘at’ is used metaphorically [cf. 59–61]. For example, in the construction *pucati na nekoga* (‘shoot on someone’ = ‘shoot at someone’), *nekoga* ‘someone’ is a landmark that metaphorically represents a potential destination where the bullet will land. This parallels the primary spatial meaning found in constructions such as *staviti knjigu na stol* ‘put a book on the table’. In examples such as (19), due to the metaphorical extension, *njega* ‘him’ is the target of an emotion. Therefore, the PP *na* ‘at’ + accusative in (19) emerges from a metaphorical chain that progresses from concrete supralocational contact between a trajector (*knjigu* ‘a book’) and a spatial landmark (*stol* ‘a table’ in *staviti knjigu na stol* ‘put a book on the table’), to a metaphorical extension with the verb *pucati* ‘shoot’. In this extension, the preposition *na* ‘on/at’ profiles the potential contact between a bullet as trajectory and a person as destination. This metaphorical progression further evolves into cases where sound functions as an abstract trajector directed toward a destination, e.g., a person. In this sense, metaphor is already present in the source domain (e.g., *Pas laje na mene.* ‘The dog barks at me.’) and is then metaphorically mapped onto humans through the conceptual metaphor PEOPLE ARE ANIMALS. The metaphor that allows *pucati na nekoga* (‘to shoot at someone’) to be extended to *lajati/režati/siktati na nekoga* (‘to bark/growl/hiss at someone’) can be described as NEGATIVE AND DANGEROUS VERBAL ACTIVITY IS NEGATIVE AND DANGEROUS PHYSICAL ACTIVITY. Additionally, the metaphor underlying the extension of the fundamental meaning of the PP *na* + accusative from constructions such as *staviti knjigu na stol* (‘to put a book on the table’) to more abstract meanings found in *pucati na nekoga* (‘to shoot at someone’) and *režati na nekoga* (‘to growl at someone’) involves two key conceptual mappings. The first is the transfer of supralocality from spatial destinations to human targets, which pertains to the accusative complement as the landmark. The second is the transfer of PROTOTYPICAL PHYSICAL SUPRALOCAL CONTACT TO ABSTRACT PHYSICAL OR SOUND-BASED SUPRALOCAL CONTACT, which relates to the preposition *na* ‘on/at’ and the trajector that is being supralocated.

(21) *I onaj starac u debeloj kabanici ljutio se na ženu*.

‘And that old man in a thick overcoat was getting angry at the woman.’

The annotators disagreed on the semantic role of this participant, with one annotating it as a recipient and the other as a patient. After discussion, we concluded that this PP could be classified as a recipient, a patient, and an experiencer. It qualifies as a recipient because the message is directed toward them, but this role is not profiled. However, since the message is delivered in a hostile manner, causing the individual to undergo a psychological change and experience fear, the individual could also be classified as an experiencer and a patient. The sequence of semantic roles is as follows: first, *Šuki* in (19) is a recipient (though this role is not profiled), as he initially hears someone’s insults; then he is simultaneously an experiencer and patient, as the negative experience of the insults directed at him and a change in his psychological state occur simultaneously. In certain contexts, *Šuki* can also be interpreted a stimulus, as his act or behavior provoked someone to howl at him, which applies to the PP *za njega* ‘for him’. We argue that, as long as the participant hears the speaker’s words, they can simultaneously assume all three (or four) semantic roles. The same conclusion was reached for the NP in the dative with the verbs *kvocati* ‘cluck=nagg’ and *siktati*_IMPF_/*prosiktati*_PF_ ‘hiss’. Assigning multiple semantic roles to a single argument is contrary to the theta-criterion [62].

When used figuratively to denote a manner of speaking, verbs of sounds made by animals are most often associated with negative emotional valence and can even be used offensively. Sounds made by small animals or those perceived as non-threatening (e.g., *kokoš* ‘hen,’ *pijetao* ‘rooster,’ *žaba* ‘frog,’ *patka* ‘duck,’ *koza* ‘goat,’ *ovca* ‘sheep’) are employed to express a negative attitude toward either the content of the speech or the interlocutor. These verbs serve to characterize the speaker or their speech as boring, irritating, or unintelligent, drawing on the specific conceptual metaphor BORING, IRRITATING, OR UNINTELLIGENT HUMAN SPEECH IS AN UNPLEASANT ANIMAL SOUND. The metaphorical mappings highlight parallels between the source domain (*animal sounds*) and the target domain (*human speech*). Specifically, the repetitive, irritating, or unintelligible qualities of animal sounds are mapped onto the corresponding attributes of human speech. For example, verbs like *njakati* ‘heehaw’ and *kvocati* ‘peck’ convey that the speaker is nagging, complaining, or being persistently annoying, reflecting the relentless and grating nature of these sounds. Similarly, verbs associated with animals producing harsh or unpleasant calls (e.g., *graktati* ‘caw’, *kreštati* ‘screech’) are used to describe a speaker with an unpleasant or grating voice, further emphasizing the negative evaluation through this metaphorical framework. As noted in [34], “the negative emotional slant of these metaphors reinforces the ideology of human superiority and disdain for animals” as well as the tendency for sex-specific pejorative metaphors to apply to females.

Verbs denoting sounds made by animals are rarely associated with positive emotional valence because humans generally perceive animals as lower beings (see, e.g., [34]), and their inarticulate sounds are considered more trivial and less refined compared to human articulated speech. When they are associated with positive emotional valence, they are typically linked to the vocalizations of particular small birds (e.g., *golub* ‘dove’, *grlica* ‘turtledove’) and convey a sense of gentle communication, often within the context of interactions between loved ones. This usage is based on the specific conceptual metaphor GENTLE, AFFECTIONATE HUMAN SPEECH IS A PLEASANT ANIMAL SOUND. The metaphorical mappings involve transferring qualities of the source domain (*bird vocalizations*) – such as softness, harmony, and a soothing nature – to the target domain (*human speech*). This mapping highlights the emotional warmth, tenderness, and intimacy associated with the speech of loved ones, drawing on the cultural and natural associations of particular bird sounds as symbols of peace, affection, and gentle interaction (14).

#### 4.2.3. Verbs of sound emission.

Verbs of sound emission, such as *bubnuti* ‘slam, *tresnuti* ‘slap’, *lupiti* ‘slam’, *klepetati* ‘clatter’, when used in their literal sense, have an inanimate entity in the subject position with the role of the theme (22), as this is an absolute single-participant thematic process conceptualized autonomously [16].

(22) *Vrata su tresnula o drveni zid*.

‘The door slammed against the wooden wall.’

However, these verbs can be used in transitive constructions, where the agent or the causer that induces the thematic process is expressed in the subject position, and the door in the object position in either the accusative or instrumental case (23).

(23) *Mašo je tresnuo vrata*_ACC_*/vratima*_INST_*.*

‘Mašo slammed the door.’

When the verbs from this semantic class are used metaphorically as verbs of speaking (see Dataset in S2 File and Table 3), the theme can be expressed by quotation (24), clausal complement (25), and NP in the accusative (26). In comparison to the prototypical verbs of speaking, the verbs from this class can have the PP *o* about’ + locative without contextualization (27), even when they are perfective (*Nije sve tako bajno, a o inim nebajnostima lupit ću u idućim kronikama.* ‘Not everything is that great, and I’ll bang=rant about other unpleasant things in the next chronicles.’). The reason for this is that these verbs do not require a direct object in their denotative meaning, as explained regarding the verbs of sounds made by animals in the previous chapter. The PP *protiv* ‘against’ + genitive was attested only with the verb *grmjeti* ‘thunder’ (28) as the verb *grmjeti* ‘thunder’ denotes the expression of negative emotions and attitudes, which aligns with the meaning of opposition. Therefore, the PP *protiv majki ubojica* in (28) has the role of maleficiary, which is opposed to the beneficiary role encoded by the PP *za* ‘for’ + accusative in (19). The PP *za* + accusative with the role of the theme was attested with the verb *zvrndati* ‘buzz’ and *bubnuti* ‘slam’ (29). The PP *na* ‘on, about’ + accusative was not attested with this verb class. A recipient can be expressed by a NP in the dative (25, 30), while the PP *na* ‘at’ + accusative was attested with the verb *grmjeti* ‘thunder’, which incorporates a component of loud voice. The motivation for the use of the PP *na* ‘at’ + accusative with the verb *grmjeti* ‘thunder’ is the same as with verbs of sounds made by animals, e.g., *režati* ‘growl’ (19), which are described in detail in Ch. 4.2.2. Regarding the NP in the dative in (25) and PP *na* ‘at’ + accusative in (19), it should be emphasized that, besides the role of the recipient, they also fulfill the roles of the patient, experiencer, and possibly stimulus (cf. Ch. 4.2.2).

**Table 3:** Valency patterns of verbs of sound emission used as VoS.

Theme	Recipient	Example
Quotation		(24) *“Hoćete li Ivana Forčića za podbana?” “Hoćemo”, tulila skupština* ‘“Do you want Ivan Forčić as deputy?” “Yes, we do”, the assembly wailed.’
Clausal complement	NP in DAT Patient/Experiencer	(25) *Hoćeš li mu zvocati da si ti planirala nešto drugo?* ‘Are you going to clang=nag to him that you had planned something else?’
NP in ACC (narrative-related)		(26) *Bude ti neugodno kad lupiš glupost.* ‘You feel embarrassed when you bang=blurt something stupid.’
PP *o* ‘about’ + LOC		(27) *Bitno je prodavati novine i klepetati o laznom suosjecanju s obitelji.* ‘It is important to sell newspapers and clatter=chatter about fake sympathy with the family.’
PP *protiv* ‘against’ + GEN	(*na* ‘at’ + ACC *grmjeti/ zagrmjeti*) Patient/Experiencer	(28) *…najlakše je onda pravedno grmjeti protiv majki ubojica.* ‘...then it is easiest to righteously thunder against women who abort.’
PP *za* ‘for, about’ + ACC		(29) *Ono što je Tolja bubnuo za Rozgu više je dosadno nego uvredljivo.* ‘What Tolja slammed=blurted for=about Rozga is more boring than offensive.’
NP in ACC(animate)	NP in DAT	(30) *Ne zna da ju je netko otkucao policiji.* ‘She doesn’t know that someone struck=snitched on her to the police.’

The general metaphor (or perhaps metonymy, see the discussion in Ch. 5) underlying all the figurative uses of these verbs is: HUMAN SPEECH IS A NONHUMAN SOUND. This encapsulates the idea that human speech is conceptualized and described through metaphors grounded in sounds produced by nonhuman entities, such as animals, objects, or natural phenomena. These metaphors emphasize various characteristics of speech – its volume, tone, quality, intent, or impact – by mapping them onto familiar nonhuman sounds that carry corresponding qualities, such as abruptness, repetitiveness, or unpleasantness.

Verbs from this class, when used metaphorically, are predominantly used in negative contexts. Verbs such as *gruvati* ‘pound’, *lupati*_IMPF_*/lupiti*_PF_ ‘bang’ (26), *tresnuti* ‘slap, blabber’ *bubnuti* ‘slam, blabber, blurt out’ (29) and *prasnuti* ‘burst’ are employed to indicate that someone has said something foolish, thoughtless, or impulsive, often in a sudden and unexpected manner. The rationale behind the use of these verbs becomes evident: just as the sound of a gun firing or a door slamming is abrupt and unforeseen, so too is the nature of an unconsidered or impulsive remark – sudden, unanticipated, and without prior deliberation. It is precisely these aspects that the conceptual metaphor highlights. The specific conceptual metaphor underlying these expressions is THOUGHTLESS SPEECH IS A LOUD, UNEXPECTED SOUND. This metaphor draws on the similarity between the abrupt, disruptive nature of loud, unforeseen sounds (e.g., a gun firing or a door slamming) and the sudden, impulsive nature of thoughtless or foolish speech. Both share qualities of being unanticipated, jarring, and lacking prior consideration.

When someone is speaking excessively, pointlessly, or in vain, verbs like *klepetati* ‘clatter’ (27), *tandrkati* ‘rattle,’ and *tuliti* ‘blare, wail’ (24) are used figuratively. The verbs *zvo(n)cati* ‘nag’ (25) and *zvrndati* ‘twirl’ are employed to describe situations where someone persistently draws attention to others’ mistakes, habits, or characteristics in a manner that irritates or annoys those around them. The source domain for these metaphorical mappings derives from the experience of irritating, repetitive sounds, such as a shutter clattering annoyingly in the wind or a horn or siren blaring incessantly. These sounds are perceived as irritating and monotonous, and it is precisely these aspects of the metaphorical mappings that are emphasized. The specific conceptual metaphor underlying these expressions is ANNOYING, REPETITIVE SPEECH IS AN ANNOYING, REPETITIVE SOUND.

When we want to express that someone has revealed a secret or snitched, the verbs *cinkati* ‘clang’ and *otkucati* ‘strike’ (30) are often used figuratively. These verbs carry interesting metaphorical associations. In their literal sense, a bell clanging or a clock striking midnight signals a transition – a beginning or an end. Similarly, in the metaphorical sense, the act of snitching represents a significant shift: the secret is no longer concealed, initiating a new reality both for the exposed individual and the one who revealed the secret. The specific conceptual metaphor underlying these expressions can be framed as REVEALING A SECRET IS A SIGNIFICANT, ATTENTION-GRABBING SOUND. The source domain involves sounds associated with important transitions, such as a bell’s clang or a clock’s chime, while the target domain pertains to the act of disclosing hidden information. The mappings highlight the transformational aspect of these actions – just as a bell or clock marks a change in time or state, snitching marks the transition from secrecy to exposure. This metaphor emphasizes the irreversible nature of the act and the consequential changes it brings to all parties involved.

The verb *šuškati* ‘rustle, whisper’ (31) is used figuratively to describe someone speaking in a low voice, either to keep something secret or in a manner that is difficult to understand. This usage is based on the specific conceptual metaphor QUIET SPEECH IS RUSTLING (source and target specification of INCOMPREHENSIBLE SPEECH IS AN INDISTINCT SOUND), where the source domain (*rustling sounds*, such as leaves or paper) maps onto the target domain (*whispering or secretive speech*). The mapping highlights qualities shared by both domains: subtlety, softness, and an element of obscurity or difficulty in being fully understood. Just as rustling sounds are often faint and indistinct, whispering is quiet and sometimes intentionally cryptic, aimed at preventing others from overhearing.

(31) *Okupljeni u povjerljivoj grupici, ljudi su šuškali da se Titovo stanje pogoršalo*.

‘Gathered in a confidential group, people rustled=whispered that Tito’s condition had worsened.’

The verbs *prozviždati* ‘whistle’ (32) and *brujati* ‘buzz’ (33) are used figuratively to indicate that certain information has become public, resulting in widespread discussion among the media and people. This usage is grounded in the specific conceptual metaphor PUBLIC SPEECH IS A LOUD SOUND, where the source domain (*loud sounds*, such as *whistling* or *buzzing*) maps onto the target domain (*widespread public discourse*). The mappings emphasize shared characteristics: just as loud sounds are pervasive, attention-grabbing, and difficult to ignore, public speech or widely circulated information is prominent, far-reaching, and capable of dominating collective attention. This metaphor highlights the intensity and omnipresence of public discourse, likening it to an environment filled with persistent, resonant noise.

(32) *Mnogi koji su prozviždali o velikim kriminalnim radnjama ostali su bez posla*.

‘Many who blew the whistle on major criminal activities lost their jobs.’

(33) *Mediji su brujali o iznosima od 13 milijuna kuna*.

‘The media were buzzing about amounts of 13 million kuna.’

#### 4.2.4. Verbs of bodily processes.

With the verbs of bodily processes, when used in their literal sense, a human or animal can be in the subject position. They are usually intransitive, but the result (34) or the goal (35) can be expressed.

(34) *Odjednom krava poskoči i valjda od muke, posere oveće govno*.

‘Suddenly, the cow jumped and, probably out of distress, shat=took a large shit.’

(35) *…je njena mačka srala po travi i onda to zakopavala.*

‘…her cat shat=took a shit on the grass and then buried it.’

When they are used figuratively as verbs of speaking, the theme can be expressed by a clausal complement (36), NP in the accusative (37), PP *o* ‘about’ + locative (38) and *protiv* ‘against’ + genitive (39) without contextualization (see Table 4). The PP *za* ‘for, about’ has a different meaning from its meaning in prototypical verbs of speaking. In (4a), the PP denotes the theme, while in (40), it is both the theme but also the stimulus, as well as the cause, since (40) can be paraphrased as *srao mi je zbog ocjena* ‘he was shitting=nagging me because of my grades’ (cf. (19)). Contextualization is not needed.

**Table 4:** Valency patterns of verbs of bodily processes used as VoS.

Theme	Recipient	Example
Clausal complement	NP in DAT	(36) *Sereš ljudima da su nepotrebni i neproduktivni, bome mi i ti djeluješ kao beskorisna jedinka.* ‘You’re shitting on people, telling them they’re unnecessary and unproductive; well, you sure seem like a useless individual to me too.’
NP in ACC		(37) *Eto, malo sam nasrao gluposti al mi smo presretni sa ovim rezultatom.* ‘Well, I shat some bullshit, but we are overjoyed with this result.’
PP *o* ‘about’ + LOC		(38) *Samo neka prestane srati o pravednosti i EMPATIJI.* ‘Just let them stop shitting=going on about righteousness and EMPATHY.’
PP *protiv* ‘against’ + GEN		(39) *Prestani više kakit protiv Keruma svaki drugi dan.* ‘Stop shitting=talking shit against Kerum every other day.’
PP *za* + ACC Stimulus	NP in DAT	(40) *Onda sam bio sa starim na kavi, pa mi je srao za ocjene* ‘Then I was having coffee with my dad, and he was shitting=nagging me for=about my grades.’
PP *po* ‘on’ + LOC		(41) …*kada ćeš početi malo kakiti po komunjarama?* ‘…when will you finally start shitting on communists?’

In (41), the PP *po* ‘on’ + locative occurs. This PP is not attested with the prototypical verbs of speaking, nor with other analysed verbs from different domains. It can be concluded that it is inherited from the source domain where it is used with the verbs *srati* ‘shit’, *kenjati* ‘crap’, *kakiti* ‘poop’, *pljuvati* ‘spit’ and denotes the goal (35). There is a semantic difference between examples with the PP *o* ‘about’ + locative (42) and *po* ‘on’ + locative (43). In (42), the person is the theme, while in (43) the person is both the theme of conversation and also the recipient or goal of criticism. With the verb *pljuvati* ‘spit’ there is a third possibility – NP in the accusative (44). With the accusative, the object is more affected, and the speaker’s negative attitude towards the object is emphasized more than when using the preposition *po* ‘on’. The fundamental meaning of the preposition *po* is distribution across a surface [60,61], and in this example it denotes that someone criticizes Kosor for one thing and then another, making her less affected than with the accusative.

(42) *I zašto ljudi stalno tako pljuju o njoj, a stvarno na temelju ničega*.

‘And why do people keep spitting about her=badmouthing her like that, really without any basis?’

(43) *Danas je moderno pljuvati po J. Kosor*.

‘Nowdays, it’s trendy to spit on J. Kosor=to badmouth J. Kosor.’

(44) *…koliko god kod kuće pljuvali domovinu, vani je počinjemo strasno voljeti*.

‘…no matter how much we spit on the homeland=badmouth the homeland at home, abroad we begin to love it passionately.’

The verbs *srati* ‘shit,’ *kenjati* ‘crap,’ *kakiti* ‘poop,’ and *pljuvati* ‘spit’ are used metaphorically as verbs of speaking to equate the thoughts or words of the speaker with feces or other excretions. This usage conveys an extremely negative attitude toward both the interlocutor’s ideas and the interlocutors themselves. These verbs function as verbs of speaking to express that someone is criticizing something or someone in an irritating, overly preachy, or unhelpful manner (36). They are also employed to label someone as a hypocrite (38) or, in some contexts, to suggest that the speaker is lying about something or someone. The underlying conceptual metaphor is NEGATIVE OR UNTRUSTWORTHY SPEECH IS BODILY WASTE OR POLLUTION. The source domain involves physical waste products, such as feces or spit, which are universally associated with unpleasantness, contamination, and rejection. These qualities map onto the target domain of human speech, highlighting the perceived lack of value, offensiveness, or harm in the speaker’s words. The mappings emphasize: a) Unpleasantness: Just as waste is undesirable and repellent, the speaker’s words are considered irritating or offensive; b) Contamination: Just as waste can pollute, the speaker’s speech is perceived as corrupting or undermining truth and integrity; and c) Rejection: Just as waste is discarded, the speaker’s ideas are dismissed as worthless or hypocritical. These metaphorical mappings underline the intensely negative evaluation.

#### 4.2.5. Verbs of deconstruction.

The verbs of deconstruction in their literal meaning (*mljeti* ‘grind’, *drobiti* ‘crush’, *piliti* ‘saw’, *rešetati*_IMPF_*/izrešetati*_PF_ ‘riddle with bullets’) are transitive verbs with the patient in the accusative (45).

(45) *U svom mlinu meljemo kukuruz, ali i pšenicu*.

‘In our mill, we grind corn, but also wheat.’

When they are used figuratively as VoS, the theme can be expressed by a clausal complement (46), NP in the accusative (47), or PPs *o* ‘about’ + locative (48) and *protiv* ‘against’ + genitive without contextualization (49) (see Table 5).

**Table 5:** Valency patterns of verbs of deconstruction used as VoS.

Theme	Recipient	Example
Clausal complement		(46) *Uostalom zasto pilim da vozi brzo kad spavam* ‘After all, why do I saw=nag that he drives fast =nag at him about driving fast.’
NP in ACC		(47) *Opet Bozanić melje gluposti.* ‘Again, Bozanić is grinding=spouting nonsense.’
PP *o* ‘about’ + LOC	NP in DAT	(48) *…. koja je doslovno svima pilila o tome kako ju u školi krivo gledaju* ‘… who sawed=nagged everyone about how they gave her dirty looks at school.’
PP *protiv* ‘against’ + GEN		(49) *A šta vi svi drobite protiv nje?* ‘Why are you all grinding=railing against her?’
PP *za* ‘for’ + ACC	NP in ACC: Patient/ Experiencer	(50) *… naši braniteljski vitezovi... koji su ga pilili za ćirilicu* ‘… our defenders of the knights... who sawed=nagged him about using Cyrillic.’
PP *o* ‘about’ + LOC	NP in ACC: Patient/ Experiencer	(51) *Kaže da su inženjeri strepili od susreta s Jobsom u dizalu, jer bi ih tada “rešetao” o tome što trenutno rade.* ‘He says that engineers dreaded meeting Jobs in the elevator because he would then “grill” them about what they were currently working on.’

With the verb *piliti* ‘saw’ (50), NP in the accusative was annotated as a patient by one annotator and as a patient/recipient by the other. Similarly, this was the case with the verb *rešetati*_IMPF_*/izrešetati*_PERF_ ‘riddle with bullets’ (51). We agreed that it can represent both, as well as an experiencer. With the verb *pitati* ‘ask’, the recipient is in the accusative; thus, by analogy, this verb has the same valency pattern. Additionally, the message is addressed to them in an aggressive or frightening manner, causing a psychological change in the recipient. Consequently, it can be concluded that it also assumes the semantic role of patient and experiencer, the same as with verbs that have the PP *na* ‘at’ + accusative (19). The increase or decrease in the passivity of an entity affected by an action can be seen with these verbs. The complement of the verb *rešetati*_IMPF_*/izrešetati*_PF_ ‘riddle with bullets’ (51) is ranked higher on this scale (greater passivity) than the complement of the verb *piliti* ‘saw’ (50), while the complements of verbs such as *režati* ‘howl’ (19) would be ranked the lowest in terms of passivity. Thus, in all cases, the roles of recipient, experiencer, patient, and, possibly, stimulus are present, but the role of the patient can be graded in terms of the degree of passivity.

The verbs *mljeti* ‘grind,’ *drobiti* ‘grind, crush,’ *piliti* ‘saw’ denote repetitive, monotonous activities that produce an annoying sound. As such, they are used metaphorically as verbs of speaking to convey that the speaker or the content of the speech is boring or tiresome. In relation to these verbs, notable differences emerge in terms of the profiling of specific aspects, which stem from inferences derived from the source domain. Specifically, the verb *drobiti* is more frequently used in contexts involving speech characterized by the excessive expression of nonsense, whereas *mljeti* is more strongly associated with prolonged and irritating verbal imposition on the interlocutor and does not necessarily involve nonsensical content; rather, it conveys the notion of being tedious and overly persistent. In contrast, *drobiti* and its derivatives are more commonly used in contexts where the speaker is talking nonsense. This distinction is also reflected in the valency patterns of the verbs: *drobiti* and its derivatives require the lexicalization of an accusative object, unlike *mljeti*. The semantic extension of *drobiti* results from the metaphorical mapping of an element from the source domain, specifically the outcome of the crushing action – irregular, amorphous, and unrecognizable fragments of a material. These elements are mapped onto incoherent and meaningless words in the target domain. Conversely, the verb *mljeti* in the source domain yields a uniform, homogeneous substance (e.g., flour produced by grinding wheat or corn), which is metaphorically transferred to monotonous, dull, and uninteresting speech in the target domain. Since uniformity is typically associated with dullness and lack of interest, the verb *mljeti* aligns with the metaphor MONOTONOUS, TEDIOUS, AND IRRITATING SPEECH IS A HOMOGENEOUS OUTCOME OF PHYSICAL PROCESSING. Meanwhile, *drobiti* corresponds to the metaphor INCOHERENT AND MEANINGLESS SPEECH IS AN AMORPHOUS AND IRREGULAR OUTCOME OF PHYSICAL PROCESSING. Additionally, grinding (*mljevenje*) is a more prolonged process than crushing (*drobljenje*), which suggests that the metaphorical extension of *mljeti* is more likely to be associated with a tedious process rather than the final result. This corresponds to the metaphor THE PROCESS OF MONOTONOUS, PROLONGED, TEDIOUS, AND IRRITATING SPEECH IS A MONOTONOUS AND PROLONGED PROCESS OF PHYSICAL PROCESSING. The distinction between *mljeti* and *drobiti* in these contexts, therefore, highlights and profiles different aspects of the target domain.

The verb *rešetati*_IMPF_*/izrešetati*_PF_ ‘riddle with bullets’ is used when describing a situation in which someone aggressively asks questions, typically in rapid succession, akin to bullets flying. The underlying conceptual metaphors are BORING/IRRITATING SPEECH IS THE UNPLEASANT SOUND OF REPETITIVE ACTION and AGGRESSIVE SPEECH IS THE SOUND OF LOUD, DAMAGING ACTION. The source domains of repetitive, noisy physical activities (e.g., grinding, sawing, and riddling with bullets) are mapped onto the target domain of speech, emphasizing the negative qualities of the speaker’s actions or the content of their speech. The mappings highlight repetition (conceptualizing the interlocutor’s words as monotonous or tiresome), annoyance or intrusiveness, and aggression (in the case of the verb *rešetati*_IMPF_*/izrešetati*_PF_ emphasizing the overwhelming or intrusive nature of the speech).

#### 4.2.6. Verbs of throwing.

With verbs of throwing in their literal sense (*dobaciti* ‘throw, toss’, *nabaciti* ‘throw, toss on’, *nabacati* ‘throw around, scatter’, *nabacivati* ‘throw around continuously’), the theme and the recipient or goal can be expressed (52).

(52) *Mogao je lako dobaciti loptu prvom svojem igraču sa baze*.

‘He could easily toss the ball to his first baseman.’

When they are used figuratively as VoS, the theme can be expressed by a quotation (53), clausal complement (54), NP in the accusative (55) and PP *o* ‘about’ + locative (56) (see Table 6).

**Table 6:** Valency patterns of verbs of throwing used as VoS.

Theme	Recipient	Example
Quotation	NP in DAT	(53) *Nakon utakmice u prolazu je novinarima dobacio: “Puklo je, gotov sam.”* ‘After the match, as he was passing by, he threw out=called out to the reporters: ‘It’s snapped, I’m done.’
Clausal complement	NP in DAT	(54) *Na takve optužbe Čeljuska mu je dobacio kako on njegovu ženu ne bi dotaknuo ni štapom.* ‘In response to such accusations, Čeljuska threw=retorted (to him) that he wouldn’t touch his wife with a stick.’
NP in ACC	NP in DAT	(55) *Kad ju je Hanibal prije tri ili Četiri godine prvi put vidio kano gospodju, bilo je oboje tako već umireno, da je on mogao nabaciti joj nekoliko običnih komplimenata.* ‘When Hannibal first saw her, they were both so calm that he was able to throw= give her a few ordinary compliments.’
PP *o* ‘about’ + LOC	NP in DAT	(56) *Kad me nadje slučajno samu, dršćuć vas zasopljen mi nabaci o ljubavi i malo ne uteČe od stida.* ‘When he happens to find me alone, trembling and out of breath, he toss about love=stammers something about love and almost runs away in embarrassment.’

The verbs of throwing are used figuratively as verbs of speaking in neutral or mildly negative contexts. These verbs express quick comments, sharp or rapid replies, or responses to something that has been said, often intended as a defense or counterargument. Additionally, they are used to describe poking – a quick, usually witty, and sometimes provocative remark. It is interesting that the recipient is expressed more often with this class of verbs than with any other, including prototypical verbs of speaking. This is because these verbs, both in their literal and figurative senses, involve a prototypical recipient, as the underlying conceptual metaphor is SPEECH IS THE THROWING OF OBJECTS, which is a specification of the more general CONDUIT METAPHOR [63,64], where COMMUNICATION IS SENDING and THOUGHTS ARE OBJECTS. The source domain involves physical actions of throwing or scattering objects, and the target domain pertains to speech acts. In line with the conduit metaphor, the mappings emphasize transference (the act of object exchange symbolizes the transfer of an idea or message from one person to another), but the specific choice of throwing verbs highlights the manner of throwing/speech, i.e., speed, precision, swiftness, and directness of verbal replies or comments, as throwing an object can be a quick, targeted action, capturing the dynamic and sometimes aggressive nature of the interaction. These verbs conceptualize communication as the rapid exchange of ideas, akin to objects being thrown back and forth in a physical interaction. As the examples suggest, *nabaciti* ‘throw around, scatter’ generally implies uttering several consecutive comments. This results in greater speed, lower precision, and less focus by the agent on the interlocutor as the target. This verb also reflects the lower significance of the comments. If the speaker wishes to convey something important to the interlocutor, they will express it with the verb accompanied by a complement in the singular, i.e., they will use the verb *dobaciti* ‘to throw a single comment’, thereby emphasizing the importance of what is being said. For this reason, the verb *nabaciti* typically pairs with plural complements, while *dobaciti* is usually accompanied by singular ones.

#### 4.2.7. Verbs of crying.

Verbs such as *jaukati*_IMPF_*/jauknuti*_PF_ ‘moan’, *jecati*_IMPF_*/zajecati*_PF_ ‘sob’, *ječati* ‘groan’, *kmečati* ‘whine’, *naricati* ‘wail, lament’, *plakati* ‘cry’ can, in their literal sense, be classified as verbs of crying, but also psych-verbs since an emotional component is emphasized.

When they are used figuratively as VoS, the theme can be expressed by a quotation (57), clausal complement (58), or the PP *o* ‘about’ + locative (59), which were attested with the prototypical VoS (see Table 7).

**Table 7:** Valency patterns of verbs of crying used as VoS.

Theme	Recipient	Example
Quotation		(57) *Zašto, zašto? - jaukala je pokrivajući uši dlanovima.* ‘Why, why?, she wailed, covering her ears with her palms.
Clausal complement		(58) *Samo je jecala kako njezina sina više nema.* ‘She just sobbed that her son is gone.’ ‘She was sobbing about her son being gone.’
PP *o* ‘about’ + LOC		(59) *Priča nema happy end i završava se uzdahom glavnog junaka dok kroz suze jeca o svojoj sudbini.* ‘The story has no happy ending and it concludes with the protagonist’s sigh as he is sobbing through tears about his fate.’
PP *nad* ‘over’ + INST		(60) …*u ta vremena lijepa naša nije jaukala nad natalitetom.* ‘…in those times, our beautiful country did not lament over its birthrate.’
PP *za* ‘afer’ + INST		(61) *…ranjeni putnici zvali u pomoć i jaukali za svojim gubicima.* ‘…wounded passengers called for help and wailed after=about their losses.’

However, with these verbs, the theme can also be expressed by the prepositional phrase *nad* ‘over’ + instrumental (60) or *za* ‘after’ + instrumental (61). These PPs are attested with psych-verbs such as *žaliti* ‘mourn’ (62–63), *patiti* ‘suffer’, *Čeznuti* ‘long’, *tugovati* ‘grieve’, and they denote a participant who is no longer present, which causes sadness.

(62) *Žalit će oni za tobom jer si dobro radila*.

‘They will mourn for you because you worked well.’

(63) *Zatvoriti se u kuću i žaliti nad svojom sudbinom, a ne poduzeti ništa u vezi toga?*

‘To lock yourself in the house and lament over your fate, and to not do anything about it?’

In this case, we could interpret the PPs *nad* ‘over’ and *za* ‘after’ + instrumental, as well as the clausal complement, as a stimulus, since with psych-verbs, the causer or cause of an emotional state is profiled. Consequently, in (57)–(61), while speaking, an emotional component is emphasized, and the participant in the subject position can be identified as both an agent and an experiencer.

Verbs of crying, when used figuratively as verbs of speaking, are not immediately associated with a negative attitude; rather, they often convey sympathy for the person speaking through tears or pain. However, the verb *kmečati* ‘whine’, typically used to describe a spoiled or overly dramatic child crying without legitimate reason, can indicate that the speaker or the content of their speech is boring or overly dramatic (64). Verbs such as *naricati* ‘lament, wail’, *plakati* ‘cry’, and *jaukati*_IMPF_*/jauknuti*_PF_ ‘wail’ can be used in both sympathetic and negative contexts, depending on the situation (cf. 58 and 65). The underlying conceptual metaphor is EMOTIONAL SPEECH IS CRYING OR WAILING. The source domain involves sounds of crying, moaning, or wailing, which are inherently metonymically linked to emotional expressions through the general metonymy EFFECT (OF THE EMOTION) FOR THE CAUSE (OF THE EMOTION), while the target domain pertains to speech that conveys or elicits strong emotions. The metaphorical mappings highlight the intensity of emotion (just as crying or wailing reflects intense emotional states, these verbs suggest that the speech is deeply emotional, whether due to pain, sadness, or frustration, and monotony or irritation in cases such as *kmečati* ‘whine’ with the target frame of the repetitive, grating nature of a child’s unsubstantiated crying. The context of use determines whether the mapping highlights sympathy (for genuine emotional distress) or annoyance (for perceived excessiveness or triviality).

(64) *…stalno smo kmečali da hoćemo veliki studio*.

‘…we kept whining that we wanted a big studio.’

(65) *SAMI ste to odlučili, sada plačete kako je to nepravedno*.

‘YOU decided that yourselves, and now you’re crying about how unfair it is.’

#### 4.2.8. Verbs of breathing.

The verbs *dahtati*_IMPF_*/dahnuti*_PF_ ‘gasp’, *soptati* ‘chug’, *uzdisati*_IMPF_*/uzdahnuti*_PF_ ‘sigh’ are intransitive when they are used in their literal sense. In some cases, they are used figuratively as VoS with the theme expressed by a quotation (66) or a clausal complement (67) (see Table 8).

**Table 8:** Valency patterns of verbs of breathing used as VoS.

Theme	Recipient	Example
Quotation	NP in the dative	(66) *Jurka - dahnu ona baki - u prvome mjestu nabavi mojoj jagodi što gođ ljepšeg nađete.* ‘Jurka - she breathed=whispered to grandmother - first of all, get my darling the prettiest thing you find.’
Clausal complement		(67) *Nitko od moje generacije ne stenje da mu je loše.* ‘Nobody from my generation moans that they are doing poorly = moans about not doing well.’

When used in this way (66–67), the emphasis is placed on the psychophysical state of the speaker. The focus is on the flow of air associated with breathing, typically caused by fatigue, excitement, or passion. The underlying conceptual metaphor is (MANNER OF) SPEAKING IS (MANNER OF) BREATHING. This metaphor highlights the connection between emotional states and verbal expression. The source domain (*breathing patterns*) is mapped onto the target domain (*manner of speech*), with irregular or exaggerated breathing reflecting physical or emotional states, such as fatigue, excitement, or passion. *Gasping*, *chugging*, or *sighing* all highlight effort or intensity, mapping onto the speaker’s verbal delivery to emphasize emotional or physical strain. In [3], these figurative meaning transfers were interpreted as metonymically motivated. However, we argue that these cases involve an interplay between metaphor and metonymy. Specifically, the elevated emotional state (the highlighted meaning in these figurative uses) causes exaggerated or unusual breathing, which, in turn, affects the speech, making it sound unusual. The movement of air is physically present in both the acts of breathing and speaking, which occur simultaneously, potentially prompting the interpretation that they belong to the same semantic frame. Nevertheless, we propose that these are two distinct, albeit conflated, domains. Furthermore, to metaphorically highlight the overly emotional manner of speech, we must incorporate a metaphorical mapping from the domain of an elevated emotional state. In our interpretation, the manner of breathing metonymically (via the general metonymy EFFECT FOR CAUSE) represents an emotionally charged state, which is then metaphorically mapped onto the distinct domain of speaking.

### 4.3. Is the passivization of transitive verbs possible in metaphorical contexts?

Regarding passivization, the following question arises: when a primarily intransitive verb is employed with an object, is passivization possible, and what does that indicate about the object’s status?

We will now zoom in on examples featuring direct objects in the active voice. When other types of objects are involved, the constructions tend to be impersonal rather than passive. Compare for example (68b) and (69b):

(68a) *…nisu cijelo vrijeme kokodakale o ljepoti.*

‘…they weren’t clucking=rabbiting on about beauty the whole time.’

(68b) *Nije kokodakano o ljepoti cijelo vrijeme.*

‘There was no clucking=rabbiting on about beauty the whole time.’

(69a) *…mi smo lajali o dignitetu DR.*

‘…we were barking=yapping about the dignity of DR.’

(69b) *Lajano je o dignitetu DR.*

‘There was barking=yapping about the dignity of DR.’

Concerning the examples featuring direct objects, the metaphorical extensions will be compared to the prototypical concrete meanings. Specifically, we will juxtapose metaphorical examples (71 and 73) to their non-metaphorical transitive counterparts (70 and 72):

(70) *Ovo je meso dobro jer su ga mljeli dva puta*.

‘This meat is good because they ground it twice.’

(70a) *Ovo je meso dobro jer je mljeveno dva puta.*

‘This meat is good because it was ground twice.’

(71) *Bozanić je opet mljeo gluposti*.

‘Bozanić talked (lit. ground) nonsense again.’

(71a) *Gluposti su opet mljevene od Bozanića.

‘Nonsense was talked (lit. ground) again by Bozanić.’

(72) … *drobili [su] grožđe u Međimurju.*

‘…they were crushing grapes in Međimurje.’

(72a) *Grož*đ*e je drobljeno u Me*đ*imurju.*

‘The grapes were crushed in Međimurje.’

(73) *Bozanić je opet drobio gluposti*.

‘Bozanić talked (lit. ground) nonsense again.’

(73a) *Gluposti su opet drobljene od Bozanića.

‘Nonsense was talked (lit. ground) again by Bozanić.’

Metaphorically intended passives are unacceptable because they violate two criteria of the prototypical scenario of transitivity [23,65–67]:

1) Entities must be in contact; a dynamic energy transfer should unfold along an action chain starting with the subject and moving toward the object; as the target entity, this object ends up being affected and undergoes a change.
2) Every prototypical transitivity scenario assumes a conceptual distinctness between subject and object; they are two distinct entities. Participants in a transitive event are concrete entities that must be maximally distinct and asymmetrical (the opposition agent/patient (theme), affected/effected objects).

These criteria are met in examples (70) and (72). In the metaphorical extension, though, an abstract entity (words) substitutes for the physical entity. The lack of contact and of the dynamic energy transfer between subject and object pushes the scenario toward the periphery of transitivity. The criterion of conceptual distinctness is flouted too. The entities are not conceptually distinct. Rather, they participate in a PART/WHOLE relationship, i.e., the object is a part of the subject. As an effected entity, it emerges from the activity itself – consequently, passivization is not possible, as witnessed by (71a) and (73a). A similar case arises with the verbs *tresnuti* ‘lit. slam’ and *lupati* ‘lit. bang’. Compare (74) and (75):

(74) *On ih je tresnuo o pod*.

‘He slammed them onto the floor.’

(74a) *Oni su tresnuti o pod.*

‘They were slammed onto the floor.’

(75) …*pa sam jednostavno tresnuo glupost*.

‘…so I just blurted out (lit. slammed) some nonsense.’

(75a) *Glupost je tresnuta.

‘*Nonsense was blurted (lit. slammed).’

It is the opposite with the verb *otkucati* ‘strike’. Its prototypical meaning involves a PART/WHOLE scenario, which precludes the passive, cf. (76) and (76a):

(76) *Sat je otkucao podne*.

‘The clock struck noon.’

(76a) *Podne je otkucano od sata.

‘Noon was struck by the clock.’

In the metaphorical meaning, in turn, the subject and the object are conceptually distinct, which makes passivization possible, cf. (77) and (77a):

(77) *Netko ga je otkucao policiji*.

‘Someone ratted him out (lit. slammed) to the police.’

(77a) *Otkucan je policiji.*

‘He was ratted out (lit. slammed) to the police.’

The verb *poklopiti* ‘cover’ is a good illustration of the ‘contact’ criterion. According to this criterion, there is a dynamic energy transfer within the action chain from the subject to the object, resulting in the affectedness and change of the latter. In the metaphorical construal, the verb is no longer passivizable, cf. (78) and (78a) as well as (79) and (79a):

(78) *…on poklopi kamenom onu rupu, u kojoj je zmija bila*.

‘…he covered the hole where the snake had been with a stone.’

(78a) *Rupa u kojoj je zmija bila poklopljena je kamenom.*

‘The hole where the snake had been was covered with a stone.’

(79) *Poklopio ga je odmah švedski premijer Fredrik Reinfeldt, izjavivši da…*

‘He was immediately put down (lit. covered) by the Swedish Prime Minister Fredrik Reinfeldt, who stated that…’

(79a)*?Poklopljen je odmah od švedskog premijera…*

‘He was immediately put down (lit. covered) by the Swedish Prime Minister…’

Also interesting are the verbs *nabaciti* ‘lit. throw something onto something’ and *dobaciti* ‘lit. throw something to someone’, cf. (80–83):

(80) *Samo su malo zemlje nabacili na to*.

‘They just threw a bit of dirt on it.’

(80a) *Malo zemlje je nabačeno na to.*

‘A bit of dirt was thrown on it.’

(81) …*nabacili su mu pokoji negativni komentar*.

‘…they tossed a few negative comments his way.’

(81a*)??Nabačen mu je pokoji negativni komentar*.

‘A few negative comments were tossed his way.’

(82) *…nakon što je djevojčica igračima dobacila loptu za početak igre*.

‘…after the little girl threw the ball to the players to start the game.’

(82a) *Lopta im je dobačena.*

‘The ball was thrown to them.’

(83) *…a njima sam dobacio komentar da na mladima svijet ostaje*.

‘…and I threw them a comment that the world belongs to the young.’

(83a*)??Dobačen im je komentar da na mladima svijet ostaje*.

‘A comment was thrown at them that the world belongs to the young.’

Like the verbs featured in examples (80) and (82), these verbs are also passivizable in their prototypical readings, where they evoke scenarios with an affected concrete object entity targeted by a conceptually distinct subject in a dynamic energy transfer. However, these verbs surface very frequently in their metaphorical readings (probably even more so than in their literal readings) (81, 83), where they feature effected abstract objects resulting from verbal activity. Consequently, although they also manifest a PART/WHOLE relationship between the object *rijeČi* ‘words’ as PARTS and the subject as a WHOLE, the passive is not entirely unacceptable; rather, it is marked (81a and 83a). Because they are so high in frequency, these metaphorical VPs have become entrenched with abstract effected objects, and their passives have become more acceptable. In other words, with their entrenchment, the PART/WHOLE relationship and the physical contact between the entities become less of an obstacle for passivization.

## 5. Discussion and conclusions

In this paper, we have presented the dataset containing a large number of Croatian metaphorical verbs of speaking annotated for their semantic roles and syntactic structure. We then thoroughly analyzed the underlying metaphorical mappings and connected the potential change of the valency pattern and the figurative meaning extension.

Before summarizing our answer to the first RQ – *What source domains are used for verbs (of manner) of speaking as targets?* – it is important to note that even prototypical constructions with verbs of speaking (i.e., *to say something to someone*), are in fact metaphorical constructions. They hinge on the ‘conduit metaphor’ that construes communication as an act of transfer [63,64,68] through the metaphors IDEAS ARE OBJECTS, LINGUISTIC EXPRESSIONS ARE CONTAINERS, COMMUNICATION IS SENDING, drawing on the ‘transfer caused motion’ extension. This figurative pattern is also found with verbs of manner of speech as reported in [69]. To treat figurative uses in our dataset as figurative extensions that can themselves involve additional extensions would make the analysis hard to follow. Therefore, for the sake of clarity, and since the change of the verb valency pattern as a result of figurative meaning extension was our focus, we treated prototypical verbs of speaking as literal senses.

Another general observation concerns the question of whether these figurative uses of verbs from various source domains as verbs of manner of speaking are metaphorically or metonymically motivated. Our analysis has demonstrated that metaphor is the predominant motivation for these uses, except in the case of verbs of breathing and crying where a partial metonymic interpretation can be justified. However, one might argue that the general conceptualizations underlying all these figurative verb uses could be understood as instances of frame metonymy. The term *frame metonymy* refers to “all usages where one reference to an element of a frame is used to refer to either the frame as a whole or to other associated elements of the frame” [21]. If we treat sound emission as a semantic frame, then the different sounds analyzed in this paper (e.g., sounds of human speech, animal sounds, sounds of object deconstruction, pleasant sounds, annoying sounds, loud sounds, etc.) could be considered elements within this frame. In this interpretation, all these cases would fall under the category of frame metonymy, as the figurative use of each verb involves referring to one element of the sound emission frame to evoke another.

Relying on the framework of the Great Chain of Being, we observe that verbs with a human as the subject in their source domain can be used in a range of contexts, from neutral, as seen with verbs of crying and breathing, to extremely negative, as with verbs associated with bodily processes. The latter are often used to indicate that someone is criticizing others, being overly preachy, hypocritical, or lying. Positioned between these extremes are verbs of throwing, which metaphorically represent sharp replies or poking. Our analysis revealed that 93.51% of the verbs denoting sounds made by animals are used in negative contexts. Their positive usage is rare and typically associated with the chirping of small birds, often in romantic contexts. Verbs with inorganic substances as subjects are similarly infrequent in positive contexts, with the notable exception of *buzz*. They appear rather more often in neutral contexts, such as describing someone speaking very softly or incomprehensibly, or when information becomes widely known. Nonetheless, these verbs are predominantly employed in negative contexts, further emphasizing their critical or dismissive connotations.

The answer to the second RQ – *Do verbs retain their valency patterns from the source domain, or do they adopt new patterns characteristic of the target domain?* – is that verbs from different source domains adopt new valency patterns from the target domain, yet they also carry over patterns from the source domain (see Appendix 2 in S3 File). The main difference between these verbs and prototypical verbs of speaking is that they are often intransitive in their source domain (verbs of sounds made by animals, verbs of sound emission, verbs of breathing). However, when used as VoS, they become transitive or ditransitive. Some of these verbs involve two participants in the source domain (verbs of bodily processes, verbs of deconstruction), while as VoS, they can have three participants. Verbs of throwing, on the other hand are ditransitive in both the source and target domain, but with different valency patterns. The analysed prototypical verbs of speaking and verbs from different domains figuratively used as VoS can have the theme expressed by a quotation, clausal complement, or an NP in the accusative, which always denotes narrative-related expressions. The difference between general VoS and those from different domains figuratively used as VoS lies in their use of the prepositional phrase *za* ‘for, about’ + accusative. Namely, with VoS, this phrase denotes the theme (4), as does the prepositional phrase *o* ‘about’ + locative. However, with verbs from the domain of sounds made by animals (*režati* ‘growl’, *graknuti* ‘caw’), there is an additional component of desire or effort to achieve something in someone’s favor (19). This use of the PP *za* ‘for’ + accusative is directly motivated by the construction *navijati za koga* ‘cheer for someone’. Another related construction is *glasati za koga* ‘vote for someone’. However, *navijati* ‘cheer’ implies a sound emission, making it a direct motivator for verbs like *režati* ‘growl’. These constructions reflect the schematic accusative meaning of a goal, as the well-being of the entity expressed by the PP *za* ‘for’ + accusative serves as the speaker’s ultimate objective. Additionally, with the verbs *piliti* ‘saw’ (50) and *srati* ‘shit’ (40), the PP *za* ‘for’ + accusative denotes the stimulus for someone being criticized.

Regarding the NP in the accusative and the PP *o* ‘about’ + locative with these verbs, contextualization of the theme is not necessary, even when they are perfective, in contrast to the perfective verbs *reći* and *kazati* ‘say, tell’, and similar to the imperfective verb *govoriti* ‘speak’ and *pričati* ‘tell’. The PP *protiv* ‘against’ + genitive, which appears with general verbs of speaking both with contextualization (*reći* ‘say’ and *kazati* ‘tell’) or without contextualization (*govoriti* ‘speak’ and *pričati* ‘talk’), is attested with verbs of sounds made by animals, bodily processes, and the verb *grmjeti* ‘thunder’ without contextualization. This PP has the role of a maleficiary, opposed to the PP *za* ‘for’ + accusative which serves as a beneficiary (cf. Ch. 4.2.3).

The PP *na* ‘on, about’ + accusative, which occurs with the verbs *reći* ‘say’ and *kazati* ‘tell’, is attested only with the verb *kokodaknuti* ‘cluck’ in a single example. Interestingly, this PP occurs exclusively with the perfective verbs *reći* ‘say’ and *kazati* ‘tell’, while it is not possible with imperfective verbs of speaking or any verb of manner of speaking (cf. Ch. 4.2.1).

Three questions arise concerning the *na* ‘on, about’ + accusative constructions:

1. Why are they restricted to perfective forms (cf. (84a–85a) and (84b–85b))?

(84a) *Što ti* **kažeš** *na to*?

‘What do you say about it?’

(84b) *Što ti **kazuješ** *na to*?

‘*What are you talking about it?’

(85a) *Što je on* **rekao** *na to*?

‘What did he say about it?’

(85b) *Što je on **govorio** *na to*?

‘*What was he speaking about it?’

1. Do they involve metaphor or metonymy?
2. Why are the constructions restricted to two or three general verbs of speaking (*reći* ‘say, tell’, *kazati* ‘say, tell’, or the dialectal *veliti* ‘say, tell’, but do not accept more specific verbs like *šapnuti* ‘whisper’, *ispričati* ‘recount’, *izustiti* ‘utter’ as in (86–88)?

(86) *Što ti **šapneš** *na to*?

‘What do you whisper about it?’

(87) *Što ti **ispričaš** *na to*?

‘What do you recount about it?’

(88) *Što ti **izustiš** *na to*?

‘What do you utter about it?’

Let’s start from the very meaning of constructions like (84a) and (85a). Do they focus on speech or thought? We submit that (84a) should actually be understood as meaning (89a–89b), while (85a) aligns with (89c–89d).

(89a) *Što ti* **misliš** *o tome?*

‘What do you think about it?’

(89b) *Koji je tvoj* **stav** *o tome?*

‘What’s your position on that?’

(89c) *Koje je njegovo* **mišljenje** *o tome?*

‘What’s his opinion on that?’

(89d) *Koji je njegov* **stav** *o tome?*

‘What’s his position on that?’

We propose that this construction rides on a meaning shift effected by the THINKING IS SPEAKING metaphor. This metaphorical meaning is coupled with a particular choice of aspect since similar constructions with the metaphors KNOWING IS SEEING and KNOWING IS HEARING also allow perfective verbs only (cf. (90a–90b) and (91a–91b)).

(90a) **Vidim** *da ti ništa nije jasno*.

‘I see you’re clueless.’

(90b) *Gledam *da ti ništa nije jasno.*

‘*I’m watching you’re clueless.’

(91a) **Čujem** *da ti ništa nije jasno.*

‘I hear you’re clueless.’

(91b) *Slušam *da ti ništa nije jasno.*

‘*I’m listening you’re clueless.’

Using imperfectives instead would invite focus on the *duration* of the activity (absolute use), and this in turn would emphasize the verbs’ basic meaning – in our case, on speaking, and in the cases above, on listening and watching. Since this is evidently not the case, i.e., since the construction profiles thinking or knowledge, only the perfective aspect is acceptable. Its relative character makes it eligible to express not only alternative meanings of verb tenses but also alternative lexical meanings. The perfective is never used for expressing absolute meanings.

Although our examples involving knowledge most certainly involve metaphor, we could arguably also interpret them metonymically, as instances of the SPEAKING FOR THINKING metonymy. The basic difference between metaphor and metonymy involves the number of domains involved in a mapping, viz. two vs. one [7,8], namely metonymy is a cognitive process in which one conceptual entity, the vehicle, provides mental access to another conceptual entity, the target, within the same domain. KNOWING and SEEING/HEARING appear to be far easier to relate to two distinct domains than our concepts of SPEAKING and THINKING. After all, speech and thought are associated more tightly and could therefore be conceptualized as elements of the same domain. On balance, while we do prefer a metaphorical interpretation, our constructions are plausibly analyzable as metonymic too.

Regarding our third question, when it comes to the ability to undergo meaning shifts away from the basic meaning by either metaphor or metonymy, only the most neutral and most schematic verbs qualify. More specific verbs of speaking profile (or highlight) only the basic meaning, e.g., *šapnuti* ‘whisper’.

The PPs *za* ‘after’ + instrumental and *nad* ‘over’ + instrumental are attested only with verbs from the domain of crying or psych-verbs, the same as the PP *po* ‘on’ + locative from the domain of bodily processes. Therefore, these PPs are adopted from the target domain.

Regarding the recipient, across all classes, it can be expressed by an NP in the dative. However, with verbs such as *piliti* ‘saw’, *rešetati*_IMPF_/*izrešetati*_PF_ ‘riddle with bullets’, and *kvocati* ‘cluck’, this NP has the semantic role of a recipient, patient, and an experiencer, as a message is directed to them in, mainly, a hostile manner (Ch. 4.2.5). Additionally, this participant in certain contexts may have the role of the stimulus, as they provoked someone’s reaction. The same applies to the PP *na* ‘at’ + accusative, which was attested with verbs of sounds made by animals, both in their source domain and the target domain, and the verb *grmjeti* ‘thunder’. This PP appears with verbs of loud speaking and verbs that include the semantic component of negative emotions, i.e., anger directed towards the recipient (Ch. 4.2.2). In line with the inferential structure of the source domain, the recipient is more frequently expressed with verbs of throwing than with any other class.

The fact that verbs from source domains adopt new valency frames from the target domain as a result of metaphorical mapping provides additional compelling evidence for the existence of meaningful abstract constructions – specifically, Argument Structure Constructions (ASCs) – which function independently of verbs and can combine with them to enable creative linguistic uses as shown by a well-known example, *sneeze the napkin off the table*, which has arguably become emblematic of Construction Grammar (CxG) over time, particularly in illustrating the constructionist approach to argument structure [24]. Our examples of the valency pattern change as a result of a metaphorical meaning shift demonstrate that verbs can appear with arguments not explicitly subcategorized by the verb itself.

In line with this, the data showing how metaphor is used to structure the domains of Communication, Thinking and Action, and a classification of metaphoric argument structure constructions are provided in [70]. The main dimension along which metaphoric argument structure constructions are classified is whether the verb in the clause evokes the target domain or the source domain of the metaphor (*I arrived at the conclusion* (verb evokes source) vs. *He cajoled her into marrying him* (verb evokes target)).

Regarding the third RQ – *Is the passivization of transitive verbs possible in metaphorical contexts?*– it has been shown that passives are unacceptable when two criteria of the prototypical scenario of transitivity (1. entities must be in contact; 2. conceptual distinctness between the subject and the object is necessary) are violated (73a, 75a). However, it is interesting that verbs that in their prototypical meaning involve a PART/WHOLE scenario, where passivization is not possible, can be passivized in their metaphorical meaning because the subject and the object are distinct entities (77a). A third situation arises with verbs that can be passivized in their literal meaning, while with the metaphorical extensions, both criteria are violated; however, in this case, passivization is not ungrammatical, but rather it is marked. This occurs because these verbs are frequently used in their metaphorical meaning, resulting in the cognitive embedding of the VP with an abstract affected object, which enables passivization (81a, 83a).

In conclusion, our findings indicate that with a metaphorical shift in meaning, verbs also often adopt new valency patterns from the target domain. Specifically, they acquire transitive forms and valency patterns characteristic of verbs of speaking. Our examples of valency pattern change as a result of a metaphorical meaning shift demonstrate that verbs can appear with arguments not explicitly subcategorized by the verb itself.

The figurative use of verbs from various source domains as verbs of manner of speaking reveals a rich interplay of metaphor and metonymy, grounded in conceptual frameworks such as the Great Chain of Being, conduit metaphor, and frame metonymy. These verbs draw on associations with sounds, actions, and physical processes to convey nuanced evaluations of speech, ranging from irritation, monotony, and aggression to empathy and affection. While negative contexts dominate – particularly with verbs of animal sounds and bodily processes – positive uses, though rare, often highlight tenderness or romantic communication. By mapping physical or nonhuman characteristics onto human speech, these verbs offer an insight into how language encodes both the content of communication and the emotional or social attitudes toward it. This study underscores the complex cognitive mechanisms underlying figurative language and its ability to reflect cultural and social perceptions of human interaction.

## Supporting information

S1 File Appendix 1. List of abbreviations. (PDF)

S2 File Dataset. Also available on Zenodo, https://doi.org/10.5281/zenodo.15210494.
(PDF)

S3 File Appendix 2. Overview of valency patterns. (PDF)

## References

[1] Mel’Čuk I. Poverxnostnyj sintaksis russkix Čislovyx vyraženij. Wien: Institut für Slawistik der Universität Wien; 1985.

[2] Buck CD. Words of speaking and saying in the Indo-European Languages: first paper. Am J Philol. 1915;36(1):1. doi: 10.2307/289517

[3] Tuđman Vuković N. Glagoli govorenja: kognitivni modeli i jeziČna uporaba. SintaktiČko-semantiČka studija. Zagreb: Hrvatska sveuČilišna naklada; 2010.

[4] BraČ I, Birtić M. Valency patterns of manner of speaking verbs in Croatian. Open Linguistics. 2023;9(1):1–30. doi: 10.1515/opli-2022-0236

[5] Memišević A, Matešić M. Is a Human’s Bark Worse Than His Bite? Animal Sounds in Croatian Verbs of Speaking. In: Memišević A, Matešić M, editors. Meaning in Language – From Individual to Collective. Berlin: Peter Lang; 2023. pp. 43–58.

[6] Lakoff G, Turner M. More than cool reason: A field guide to poetic metaphor. Chicago: University of Chicago Press; 2009.

[7] Lakoff G, Johnson M. Metaphors we live by. Chicago: University of Chicago Press; 1980.

[8] Kövecses Z. Metaphor: A Practical Introduction. Oxford/New York: Oxford University Press; 2010.

[9] Levin B. English verb classes and alternations. Chicago: The University of Chicago Press; 1993.

[10] Vergaro C, Sandford JL, Mastrofini R, Formisano YM. ‘Hollering from across the yard’: fictive path in manner of speaking events. Lang cogn. 2014;6(3):408–26. doi: 10.1017/langcog.2014.12

[11] Sandford JL. With a friendly or critical attitude, categorizing English manner of speaking verb components. Online Proceedings of UK-CLA Meetings. 2017;4:230–48.

[12] Ahn H. The lexicalization pattern of the verbs of speaking: Categorical compositionality in Russian. Slavic East Eur J. 2013;57(2):274–96.

[13] Langacker RW. Foundations of Cognitive Grammar. Vol. 1. Stanford: Stanford University Press; 1987.

[14] Langacker RW. A Usage-Based Model. In: Rudzka-Ostyn B, editor. Topics in Cognitive Linguistics. Amsterdam: John Benjamins; 1988. pp. 127–61.

[15] Langacker RW. A Dynamic Usage-Based Model. In: Barlow M, Kemmer S, editors. Usage-based models of language. Stanford: CSLI Publications; 2000. pp. 1–63.

[16] Langacker RW. Cognitive Grammar, A Basic Introduction. Oxford: Oxford University Press; 2008.

[17] Barlow M, Kemmer S. Usage-based models of language. Stanford: CSLI Publications; 2000.

[18] Bybee J, Hopper P. Frequency and the emergence of linguistic structure. Amsterdam: John Benjamins; 2001.

[19] Lakoff G. The contemporary theory of metaphor. In: Ortony A, editor. Metaphor and thought. New York: Cambridge University Press; 1993. pp. 202–251 doi: 10.1017/CBO9781139173865.013

[20] Lakoff G, Johnson M. Philosophy in the Flesh. New York: Basic Books; 1999.

[21] Dancygier B, Sweetser E. Figurative language. Cambridge: Cambridge University Press; 2014.

[22] Langacker RW. Foundations of Cognitive Grammar. Vol. 1. Stanford: Stanford University Press; 1987.

[23] Rice SA. Towards a cognitive model of transitivity. PhD Thesis, ProQuest Dissertations & Theses Global. University of California: San Diego; 1987.

[24] Goldberg AE. Constructions: A Construction Grammar Approach to Argument Structure. Chicago: Chicago University Press; 1995.

[25] Stowell TA. Origins of phrase structure. PhD Thesis, MIT. 1981. Available from: http://www.ai.mit.edu/projects/dm/theses/stowell81.pdf

[26] Lehrer A. Checklist for verbs of speaking. Acta Linguistica Hungarica. 1988;38(1–4):143–61.

[27] Pinker S. Learnability and cognition: The acquisition of argument structure. Cambridge: MIT Press; 1989.

[28] Pesetsky D. Zero syntax. Experiencers and cascades. Cambridge: MIT Press; 1995.

[29] Dohrety C. Clauses without ‘that’: the case for bare sentential complementation in English. New York: Routledge; 2000.

[30] Erteschik-Shir N. Bridge phenomena. In: Everaert M, van Riemsdijk HC, editors. The Wiley Blackwell Companion to Syntax. Hoboken: Blackwell; 2017. pp. 284–294. doi: 10.1002/9781118358733.wbsyncom011

[31] Stoica I. The syntax and the semantics of manner of speaking verbs. University of Bucharest. 2019.

[32] Stoica I. Are manner of speaking verbs truly manner? Bucharest Working Papers Linguistics. 2020;XXII(1): 29–48.

[33] Zwicky AM. In a manner of speaking. Linguistic Inquiry. 1971;2(2):223–32.

[34] Goatly A. Humans, animals, and metaphors. Soc Animals. 2006;14(1):15–37. doi: 10.1163/156853006776137131

[35] Rakhilina E. Animal sounds: a human vantage point. Russ Contrast Oslo Stud Lang. 2010;2(2):319–38.

[36] Rakhilina E, Parina E. Les sons animaux. In: Rakhilina E, Chahine IK, Merle J-M, editors. Verba Sonandi: Représentation linguistique des cris d’animaux. Aix-en-Provence: Presses Universitaires de Provence, Aix-Marseille Universite; 2017. pp. 13–25.

[37] Beigman KB, Leong CL, Gutierrez ED, Shutova E, Flor M. Semantic classifications for detection of verb metaphors. In: Erk K, Smith NA, editors. Proceedings of the 54th Annual Meeting of the Association for Computational Linguistics (Volume 2: Short Papers). Berlin: Association for Computational Linguistics; 2016. pp. 101–106.

[38] Kipper K, Korhonen A, Ryant N, Palmer M. Extending VerbNet with Novel Verb Classes. In: Calzolari N et al., editors. Proceedings of the Fifth International Conference on Language Resources and Evaluation (LREC’06). Genoa: ELRA; 2006. pp. 1027–1032.

[39] Gibbs R. The poetics of mind: Figurative thought, language, and understanding. New York: Cambridge University Press; 1994.

[40] Grady J. Foundations of meaning: Primary metaphors and primary scenes. PhD Thesis, University of California; Berkeley: 1997. Available from: https://escholarship.org/uc/item/3g9427m2

[41] Pragglejaz Group. MIP: A Method for Identifying Metaphorically Used Words in Discourse. Metaphor Symbol. 2007;22(1):1–39. doi: 10.1080/10926480709336752

[42] Johnson CR. Constructional grounding: The role of interpretational overlap in lexical and constructional acquisition. University of California; Berkeley: 1999.

[43] Boroditsky L, Ramscar M. The roles of body and mind in abstract thought. Psychol Sci. 2002;13(2):185–9. doi: 10.1111/1467-9280.00434 11934006

[44] Despot KŠ. How language influences conceptualization: from Whorfianism to Neo-Whorfianism. Coll Antropol. 2021;45(4): 373–380. doi: 10.5671/ca.45.4.9

[45] Despot KŠ, Ostroški Anić A. A war on war metaphor: metaphorical framings in croatian discourse on Covid-19. Rasprave. 2021;47(1):173–208. doi: 10.31724/rihjj.47.1.6

[46] Barcelona A. Trends in cognitive-linguistic research on metonymy. Cognitive Linguist Stud. 2024;11(1):51–74. doi: 10.1075/cogls.00112.bar

[47] Brdar M. Metafore i metonimije u interakciji. In: Molvarec L, Pišković T, editors. Metafore u hrvatskome jeziku, književnosti i kulturi. Zagreb: Filozofski fakultet SveuČilišta u Zagrebu; 2019. pp. 51–94.

[48] Radden G, Kövecses Z. Towards a theory of metonymy. Cognitive Linguistics: Explorations, Applications, Research. Hamburg – Budapest: University of Hamburg – Eötvös Loránd University; 1996. pp. 999.

[49] Barcelona A. On the plausibility of claiming a metonymic motivation for conceptual metaphor. In: Barcelona A, editor. Metaphor and metonymy at the crossroads. Mouton de Gruyter; 2000. pp. 32–58. doi: 10.1515/9783110894677.31

[50] Goossens L. Metaphtonymy: The interaction of metaphor and metonymy in expressions of linguistic action. Cognitive Linguistics. 1990;1:323–40.

[51] Ljubešić N, KlubiČka F. {bs,hr,sr}WaC — Web corpora of Bosnian, Croatian and Serbian. In: Bildhauer F, Schäfer R, editors. Proceedings of the 9th Web as Corpus Workshop (WaC-9). Gothenburg: Association for Computational Linguistics; 2014. pp. 29–35.

[52] Brozović RD, et al. Croatian language corpus Riznica 0.1, Slovenian language resource repository CLARIN.SI; 2018. Available from: http://hdl.handle.net/11356/1180

[53] Tadić M. New version of the Croatian National Corpus. In: HlaváČková D, et al., editors. After half a century of Slavonic natural language processing. Brno: Masaryk University; 2009. pp. 199–205.

[54] Kilgarriff A, Baisa V, Bušta J, JakubíČek M, Kovář V, Michelfeit J, et al. The Sketch Engine. Lexicography. 2014;1(1):7–36. doi: 10.1007/s40607-014-0009-9

[55] Belaj B, Tanacković Faletar G. Kognitivna gramatika hrvatskoga jezika, knjiga prva, imenska sintagma i sintaksa padeža. Zagreb: Disput; 2014.

[56] Pranjković I. Glagoli govorenja i njihove dopune. Zbornik Matice srpske za slavistiku. 2007;71–72:133–41.

[57] Ruiz De Mendoza Ibáñez FJ. Metaphor, metonymy and conceptual interaction. Atlantis. 1997;19(1):281–95.

[58] Ruiz Gil E, Ruiz JH. New perspectives on the people are animals metaphor. Interlingüística. 2005;16:931–41.

[59] Brugman CM. The Story of Over: Polysemy, Semantics, and the Structure of the Lexicon. New York: Garland; 1988.

[60] Šarić Lj. Prostor u jeziku i metafora. KognitivnolingvistiČke studije o prefiksima i prijedlozima. Zagreb: Naklada Jesenski i Turk; 2014.

[61] Matovac D. Prijedlozi u hrvatskome jeziku. Zagreb: Hrvatska sveuČilišna naklada; 2017.

[62] Chomsky N. Lectures on Government and Binding. Dordrecht: Foris; 1981.

[63] Reddy MJ. The conduit metaphor: A case of frame conflict in our language about language. In: Ortony A, editor. Metaphor and thought. New York: Cambridge University Press; 1979. pp. 284–324.

[64] Lakoff G. Women, fire, and dangerous things: What categories reveal about the mind. Chicago: University of Chicago Press; 1987.

[65] Hopper PJ, Thompson SA. Transitivity in Grammar and Discourse. Language. 1980;56(2):251–99. doi: 10.1353/lan.1980.0017

[66] Hopper PJ. Causes and Affects. In: Eilfort WH, Kroeber PD, Peterson KL, editors. Papers from the Parasession on Causatives and Agentivity. Chicago: Chicago Linguistic Society; 1985. pp. 67–88.

[67] Belaj B, Tanacković FG. Kognitivna gramatika hrvatskoga jezika, knjiga druga, Sintaksa jednostavne reČenice. Zagreb: Disput; 2017.

[68] Fillmore CJ. The case for case. In: Bach E, Harms RT, editors. Universals in linguistic theory. New York: Holt, Rinehart, and Winston; 1968. pp. 1–88.

[69] Laporte S. Casting new light on the CAUSED-MOTION construction: Some evidence from Harry Potter. In: Gallez F, Hermann M, editors. Cognition and Contrast. Bruxelles: Presses universitaires Saint-Louis Bruxelles; 2022. pp. 119–132. doi: 10.4000/books.pusl.27868

[70] David O. Metaphor in the grammar of argument realization. University of California: Berkeley; 2016. Available from: https://escholarship.org/uc/item/9sk0f7xc

