# Supplementary material for: Bleating, growling, barking, and spitting: Metaphorical extensions and valency patterns of verbs of speaking
Source: PLoS One. 2025 Jun 10;20(6):e0325807. doi: 10.1371/journal.pone.0325807 (PMC12151387; doi:10.1371/journal.pone.0325807)
Supplement: S1 File — (PDF) [file pone.0325807.s001.pdf]

List of abbreviations

Syntactic marking used in the dataset and paper:

NP – noun phrase

PP – prepositional phrase

QUOT – quotation

Clausal complement

V – verb

Morphological marking used in the dataset and paper:

ACC – accusative

DAT – dative

GEN – genitive

INST – instrumental

LOC – locative

NOM – nominative

Semantic roles used in the dataset and paper:

Agent

Agent_METO

Beneficiary

Maleficiary

Patient

Recipient

Theme

Stimulus
