# Supplementary material for: Bleating, growling, barking, and spitting: Metaphorical extensions and valency patterns of verbs of speaking
Source: PLoS One. 2025 Jun 10;20(6):e0325807. doi: 10.1371/journal.pone.0325807 (PMC12151387; doi:10.1371/journal.pone.0325807)
Supplement: S2 File — Also available on Zenodo, https://doi.org/10.5281/zenodo.15210494. (PDF) [file pone.0325807.s002.pdf]

VERB	EXAMPLE	SOURCE DOMAIN	METAPHOR/METONYMY	VALENCY FRAME OF THE EXAMPLE	VALENCY FRAME IN THE SOURCE DOMAIN	VALENCY FRAME IN THE SOURCE DOMAIN	EMOTIONAL VALENCY	HIGHLIGHTED MAPPING
**govoriti 'speak'**	Jutros nije mogla govoriti.<br>'This morning she couldn't speak.'	speaking		NP_nom [Agent] V			NEUTRAL	
**govoriti 'speak'**	Govoriš ljudima da su najgenijalnija bića na ovome svijetu.<br>'You tell people that they are the most brilliant beings on this planet.'	speaking		NP_nom [Agent] V da ('that')_CP [Theme] NP_dat [Recipient]			NEUTRAL	
**govoriti 'speak'**	Govorim mađarski.<br>'I speak Hungarian.'	speaking		NP_nom [Agent] V NP_acc [Theme]			NEUTRAL	
**govoriti 'speak'**	Premijer govori gluposti.<br>'The prime minister is talking nonsense.'	speaking		NP_nom [Agent] V NP_acc [Theme]			NEUTRAL	
**govoriti 'speak'**	Svaki dan govorim igračima neke stvari.<br>'Every day I tell the players certain things.'	speaking		NP_nom [Agent] V NP_acc [Theme] NP_dat [Recipient]			NEUTRAL	
**govoriti 'speak'**	Lucien Favre ne govori lijepo o njemu.<br>'Lucien Favre doesn't speak nicely about him.'	speaking		NP_nom [Agent] V PP_o ('about')+loc [Theme]			NEUTRAL	
**govoriti 'speak'**	Najradije je govorio o svom zavičaju.<br>'He liked talking about his hometown the most.'	speaking		NP_nom [Agent] V PP_o ('about')+loc [Theme]			NEUTRAL	
**govoriti 'speak'**	Ja ću govoriti s njim o tom.<br>'I will talk to him about that.'	speaking		NP_nom [Agent] V PP_o ('about')+loc [Theme] PP_s+inst [Co_Agent]			NEUTRAL	
**govoriti 'speak'**	O tome i o aktualnoj gospodarsko-socijalnoj situaciji Kunst govori za Vjesnik.<br>'About that and the current economic and social situation Kunst talks for Vjesnik.'	speaking		NP_nom [Agent] V PP_o ('about')+loc [Theme] PP_za ('for')+acc [Recipient]			NEUTRAL	
**govoriti 'speak'**	Prije glasovanja Berlusconi će govoriti pred zastupnicima.<br>'Before the vote, Berlusconi will speak in front of the deputies.'	speaking		NP_nom [Agent] V PP_pred ('in front of')+inst [Recipient]			NEUTRAL	
**govoriti 'speak'**	Robi govori protiv Eu projekata.<br>'Robi speaks against EU projects.'	speaking		NP_nom [Agent] V PP_protiv ('against')+gen [Theme]			NEUTRAL	
**govoriti 'speak'**	Čudom se čude u Metkoviću, iako nitko neće govoriti za novine.<br>'They are marveling in disbelief in Metković, although no one wants to speak to the newspapers.'	speaking		NP_nom [Agent] V PP_za ('for')+acc [Recipient]			NEUTRAL	
**govoriti 'speak'**	...ti si govorio za Arhelaja da je sretan.<br>'You said for Archelaus that he was happy. = You said Archelaus was happy.'	speaking		NP_nom [Agent] V PP_za ('for')+acc [Theme] da ('that')_CP [Theme]			NEUTRAL	
**govoriti 'speak'**	Ni ja nisam starčić, a ti tako ružne riječi govoriš za svoju mamu.<br>'I'm not an old man either, and yet you say such ugly things about your mother.'	speaking		NP_nom [Agent] V PP_za ('for')+acc [Theme] NP_acc [Theme]			NEUTRAL	
**govoriti 'speak'**	Još dok je mladić bio, otac je govorio za njega: Ovaj moj Siman nit je na mene ni na pokojnu majku.<br>'Even when he was still a young man, his father used to say about him: This son of mine, Siman, takes after neither me nor his late mother.'	speaking		NP_nom [Agent] V PP_za ('for')+acc [Theme] QUOT [Theme]			NEUTRAL	
**govoriti 'speak'**	Neki francuski pisac je to<br>govorio za život.<br>'Some French writer was saying that about life.'	speaking		NP_nom [Agent] V PP_za ('for')+acc [Theme]NP_acc [Theme]				
**govoriti 'speak'**	Ako to nije ostvarenje američkoga sna, onda ne znam što je - govori za Reuters Tim Stodd.<br>'If that's not the American Dream come true, then I don't know what is," Tim Stodd says to Reuters.'	speaking		NP_nom [Agent] V QOUT [Theme] PP_za ('for')+acc [Recipient]			NEUTRAL	
**govoriti 'speak'**	O monumentalnosti Ceausescuove palače najbolje govori statistika.<br>'The statistics speak best to the monumentality of Ceausescu's palace.'	speaking	META	NP_nom [Agent_META] V PP_o ('about')+loc [Theme]			NEUTRAL	
**govoriti 'speak'**	Predstava govori o muškarcu koji se odlučio vratiti u svoju zemlju.<br>'The play says about the man=tells the story of a man who decided to return to his country.'	speaking	META	NP_nom [Agent_META] V PP_o ('about')+loc [Theme]			NEUTRAL	
**kazati 'tell'**	Kaži mi svoju stranu priče.<br>'Tell me your side of the story.'	speaking		NP_nom [Agent] V NP_acc [Theme] NP_dat [Recipient]			NEUTRAL	

VERB	EXAMPLE	SOURCE DOMAIN	METAPHOR/METONYMY	VALENCY FRAME OF THE EXAMPLE	VALENCY FRAME IN THE SOURCE DOMAIN	VALENCY FRAME IN THE SOURCE DOMAIN	EMOTIONAL VALENCY	HIGHLIGHTED MAPPING
**kazati 'tell'**	Nije mi htjela istinu kazati o sebi.<br>'She didn't want to tell me the truth about herself.'	speaking		NP_nom [Agent] V NP_acc [Theme] NP_dat [Recipient]			NEUTRAL	
**kazati 'tell'**	I sad neka mi netko nešto kaže protiv velikih trgovačkih centara.<br>'And now let someone try to say something against big shopping malls.'	speaking		NP_nom [Agent] V NP_acc [Theme] PP_protiv ('against')+gen [Theme]			NEUTRAL	
**kazati 'tell'**	Molim vas, ne tjerajte me da nešto kažem protiv Juventusa.<br>'Please, don't make me say something against Juventus.'	speaking		NP_nom [Agent] V NP_acc [Theme] PP_protiv ('against')+gen [Theme]			NEUTRAL	
**kazati 'tell'**	Bartol Kašić (...) kaže za ovu crkvu da ima krov.<br>'Bartol Kašić (...) says about this church that it has a roof=says that this church has a roof.'	speaking		NP_nom [Agent] V PP_za ('for')+acc [Theme] da ('that')_CP [Theme]			NEUTRAL	
**kazati 'tell'**	Ne treba tugovati - kaže otac.<br>'No need to be sad, says the father.'	speaking		NP_nom [Agent] V QUOT [Theme]			NEUTRAL	
**kazati 'tell'**	"Valja znati da je istraga o tim zločinima u završnoj fazi, i dosta je nervoze među ljudima koji su predmet te istrage", kazao nam je Hedl.<br>'"It should be noted that the investigation into these crimes is in its final phase, and there is quite a bit of nervousness among the people who are the subject of that investigation," Hedl told us.'	speaking		NP_nom [Agent] V QUOT [Theme] NP_dat [Recipient]			NEUTRAL	
**kazati 'tell'**	Zato smo se odlučili (...), kaže za Deutsche Welle Martin Faulstich (...)<br>'That's why we made the decision (...), Martin Faulstich told for Deutsche Welle=told Deutsche Welle (...)'	speaking		NP_nom [Agent] V QUOT [Theme] PP_za ('for')+acc [Recipient]			NEUTRAL	
**kazati 'tell'**	Nije htio kazati tko je ukrao pismo.<br>'He didn't want to say who stole the letter.'	speaking		NP_nom [Agent] V tko ('who')_CP [Theme]			NEUTRAL	
**pričati 'talk'**	Studenti pričaju da je cijena ispita bila - tisuću eura za dvojku.<br>'The students say that the price of the exam was – a thousand euros for a passing grade.'	speaking		NP_nom [Agent] V da ('that')_CP [Theme]			NEUTRAL	
**pričati 'talk'**	Vodič nam je pričao kako su ljudi prije izrađivali igračke i prodavali ih.<br>'The guide told us how people used to make toys and sell them.'	speaking		NP_nom [Agent] V da ('that')_CP [Theme] NP_dat [Recipient]			NEUTRAL	
**pričati 'talk'**	...opet pričaš gluposti...<br>'You're talking nonsense again.'	speaking		NP_nom [Agent] V NP_acc [Theme]			NEUTRAL	
**pričati 'talk'**	Ljudi pričaju svašta o meni.<br>'People say all sorts of things about me.'	speaking		NP_nom [Agent] V NP_acc [Theme] PP_o ('about')+loc [Theme]			NEUTRAL	
**pričati 'talk'**	Svašta su pričali protiv tebe.<br>'They talked all kinds of nonsense about you.'	speaking		NP_nom [Agent] V NP_acc [Theme] PP_protiv ('against')+gen [Theme]			NEUTRAL	
**pričati 'talk'**	S bakom rijetko kad pričam hrvatski.<br>'I rarely speak Croatian with my grandmother.'	speaking		NP_nom [Agent] V NP_acc [Theme] PP_s ('with')+inst [Co_Agent]			NEUTRAL	
**pričati 'talk'**	Kad se nađu, uvijek pričaju o nekakvim glupostima.<br>'Whenever they meet up, they're always talking about silly stuff.'	speaking		NP_nom [Agent] V PP_o ('about')+loc [Theme]			NEUTRAL	
**pričati 'talk'**	Prvo nam je pričao o slobodi i što znači biti slobodan.<br>'First, he told us about freedom and what it means to be free.'	speaking		NP_nom [Agent] V PP_o ('about')+loc [Theme]			NEUTRAL	
**pričati 'talk'**	Uglavnom pričamo o dnevnoj politici, filmovima koje smo gledali, kućnom budžetu.<br>'We mostly talk about daily politics, the movies we've watched, and the household budget.'	speaking		NP_nom [Agent] V PP_o ('about')+loc [Theme]			NEUTRAL	
**pričati 'talk'**	Nikad nisam pričala pred njim o tome.<br>'I never spoke about that in front of him.'	speaking		NP_nom [Agent] V PP_o ('about')+loc [Theme] PP_pred ('in front of')+inst [Recipient]			NEUTRAL	
**pričati 'talk'**	Svi mi pričamo s prijateljima o sexu.<br>'We all talk about sex with our friends.'	speaking		NP_nom [Agent] V PP_o ('about')+loc [Theme] PP_s+inst [Co_Agent]			NEUTRAL	

VERB	EXAMPLE	SOURCE DOMAIN	METAPHOR/METONYMY	VALENCY FRAME OF THE EXAMPLE	VALENCY FRAME IN THE SOURCE DOMAIN	VALENCY FRAME IN THE SOURCE DOMAIN	EMOTIONAL VALENCY	HIGHLIGHTED MAPPING
**pričati 'talk'**	O njihovim privatnim stvarima ne bi trebalo da pričam za medije.<br>'I shouldn't be talking for the media=to the media about their private matters.'	speaking		NP_nom [Agent] V PP_o ('about')+loc [Theme] PP_za ('for')+acc [Recipient]			NEUTRAL	
**pričati 'talk'**	Svatko priča protiv svakoga.<br>'Everyone talks against everyone.'	speaking		NP_nom [Agent] V PP_protiv ('against')+gen [Theme]			NEUTRAL	
**pričati 'talk'**	Kažeš da si već pričala sa razrednicom.<br>'You said you already talked to the homeroom teacher.'	speaking		NP_nom [Agent] V PP_s ('with')+inst [Co_Agent]			NEUTRAL	
**pričati 'talk'**	Ne pričam za HTV.<br>'I'm not talking for HTV=to HTV.'	speaking		NP_nom [Agent] V PP_za ('for')+acc [Recipient]			NEUTRAL	
**pričati 'talk'**	Ja ne govorim o Dinamu, pričam za navodni interes Šahtara za Callela.<br>'I'm not talking about Dinamo; I'm speaking about Shakhtar's supposed interest in Callelo.'	speaking		NP_nom [Agent] V PP_za ('for')+acc [Theme]			NEUTRAL	
**pričati 'talk'**	Sada ću, pored svih fotografija u albumu svog života, eto, imati i ove ", priča za Jutarnji list.<br>'Now, in addition to all the photos in the album of my life, I'll have these as well, he talks for Jutarnji list=he says to Jutarnji list.'	speaking		NP_nom [Agent] V QUOT [Theme] PP_za ('for')+acc [Recipient]			NEUTRAL	
**reći 'say'**	Vi ste rekli da je hrvatski jezik krasan.<br>'You said that the Croatian language is beautiful.'	speaking		NP_nom [Agent] V da ('that')_CP [Theme]			NEUTRAL	
**reći 'say'**	Policiji su rekli da su bili umorni od dalekog puta.<br>'They told the police that they were tired from the long journey.'	speaking		NP_nom [Agent] V da ('that')_CP [Theme] NP_dat [Recipient]			NEUTRAL	
**reći 'say'**	izvori bliski vladajućoj koaliciji rekli su za Reuters kako ne treba računati na raspisivanje prijevremenih izbora	speaking		NP_nom [Agent] V kako ('that')_CP [Theme] PP_za ('for')+acc [Recipient]			NEUTRAL	
**reći 'say'**	Kakvo je to ponašanje, reći ću te mami.<br>'What kind of behavior is that, I'm telling you to your mom= I'm telling your mom!'	speaking		NP_nom [Agent] V NP_acc [Patient]/[Theme] NP_dat [Recipient]			NEG	
**reći 'say'**	Netko je i rekao sličnu šalu.	speaking		NP_nom [Agent] V NP_acc [Theme]			NEUTRAL	
**reći 'say'**	...grad Split ni riječ ne reče na gubitak takvog znanstvenika.<br>'...the city of Split did not say a word on=about the loss of such a scientist.'	speaking		NP_nom [Agent] V NP_acc [Theme] PP_na ('at')+acc [Theme]			NEUTRAL	
**reći 'say'**	To je rekao pred desetak novinara.<br>'He said that in front of about ten journalists.'	speaking		NP_nom [Agent] V NP_acc [Theme] PP_pred ('in front of')+inst [Recipient]			NEUTRAL	
**reći 'say'**	Nikad nisam ništa rekao protiv suca.<br>'I've never said anything against the judge.'	speaking		NP_nom [Agent] V NP_acc [Theme] PP_protiv ('against')+gen [Theme]			NEUTRAL	
**reći 'say'**	O svojoj ideji zaljubljenici znanstvene fantastike nisu ništa rekli ni matičarki.<br>'The sci-fi fans didn't say anything about their idea - not even to the registrar.'	speaking		NP_nom [Agent] V PP_o ('about')+loc [Theme] NP_dat [Recipient]			NEUTRAL	
**reći 'say'**	...o njemu su svi rekli što misle i što ne misle.<br>'...everyone said about him both what they really think and what they don't.'	speaking		NP_nom [Agent] V PP_o ('about')+loc [Theme] što_CP [Theme]			NEUTRAL	
**reći 'say'**	Zanimljivo je i možda ne toliko poznato, da je Tolstoj rekao za Shakespearea da je vrlo površan i loš spisatelj.<br>'It's interesting — and perhaps not so well known — that Tolstoy said (that) Shakespeare was a very superficial and poor writer.'	speaking		NP_nom [Agent] V PP_za ('for')+acc [Theme] da ('that')_CP [Topic]			NEUTRAL	
**reći 'say'**	Ne znam zašto sam vam uopće rekla za bebu.<br>'I don't know why I even told you about the baby.'	speaking		NP_nom [Agent] V PP_za ('for')+acc [Theme] NP_dat [Recipient]			NEUTRAL	
**reći 'say'**	»Čovik i po« rekli bi za njega Šibenčani.<br>'"A man and a half," is what the people of Šibenik would say about him.'	speaking		NP_nom [Agent] V PP_za ('for')+acc [Theme] QUOT [Topic]			NEUTRAL	

VERB	EXAMPLE	SOURCE DOMAIN	METAPHOR/METONYMY	VALENCY FRAME OF THE EXAMPLE	VALENCY FRAME IN THE SOURCE DOMAIN	VALENCY FRAME IN THE SOURCE DOMAIN	EMOTIONAL VALENCY	HIGHLIGHTED MAPPING
**reći 'say'**	Sabina se bila razljutila na svoga muža kad je kucao na vratima i rekla Zorki: - Čudan je tvoj otac!<br>'Sabina got upset with her husband when he knocked on the door and said to Zorka, "Your father is strange!"'	speaking		NP_nom [Agent] V QUOT [Theme] NP_dat [Recipient]			NEUTRAL	
**reći 'say'**	Facebook je samo ponovio ono što je Sullivan već rekao za Reuters: " U kompaniji nikad nismo željeli okruženje u kojem zaposlenici mogu imati uvid u privatnu komunikaciju, tako da je zaista važno koristiti tehnologiju. "<br>'Facebook merely repeated what Sullivan had already told (for) Reuters: "At the company, we never wanted an environment where employees could access private communication, so it's really important to use technology."'	speaking		NP_nom [Agent] V QUOT [Theme] PP_za ('for')+acc [Recipient]			NEUTRAL	
**blejati 'bleat'**	...jednako kao što amerika prdne da donosi "demokraciju" i domah svi bleje da je nastupila - demokracija.<br>'...just like when America farts claiming it's bringing "democracy," and right away everyone bleats=gapes as if — democracy has arrived.'	animal	META	NP_nom [Agent] V da ('that')_CP [Theme]	NP_nom [Agent] V		NEG	collective, stupid
**blejati 'bleat'**	I potpitanje, zašto blejiš gluposti o kojima pojma nemaš i samim time autoru teme otežavaš situaciju?<br>'And a follow-up question: why are you bleating=spouting nonsense about things you clearly know nothing about, and in doing so, making things harder for the author of the topic?'	animal	META	NP_nom [Agent] V NP_acc [Theme]	NP_nom [Agent] V		NEG	collective, stupid
**blejati 'bleat'**	...bleje o zastavama dok jedva kruha imaju za jesti.<br>'...they bleat=babble on about flags while they can barely afford bread to eat.'	animal	META	NP_nom [Agent] V PP_o ('about')+loc [Theme]	NP_nom [Agent] V		NEG	collective, stupid
**blejati 'bleat'**	Bleje protiv čovika koji je uljudija Split.<br>'They're bleating=blabbering against the man who made Split more civilized.'	animal	META	NP_nom [Agent] V PP_protiv ('against')+gen [Theme]	NP_nom [Agent] V		NEG	collective, stupid
**blejati 'bleat'**	...pa umjesto što okolo blejite protiv svega što je vezano za Tita, i vi sami napravite nešto slično po vašem ćefu i uživate.<br>'...instead of bleating=ranting against everything related to Tito, why don't you do something similar your own way and enjoy it?'	animal	META	NP_nom [Agent] V PP_protiv ('against')+gen [Theme]	NP_nom [Agent] V		NEG	collective, stupid
**brbotati 'babble'**	Daj ne brbotaj bljezgarije.<br>'Stop babbling nonsense.'	animal	META	NP_nom [Agent] V NP_acc [Theme]	NP_nom [Agent] V		NEG	irritating, stupid
**brundati 'grumble'**	Mnogi brundaju kako je Valentinovo previše komercijalno i kako to sve skupa nema nikakvog smisla.<br>'Many grumble=complain that Valentine's Day is too commercialized and that it all makes no sense.'	animal	META	NP_nom [Agent] V kako ('that')_CP [Theme]	NP_nom [Agent] V	NP_nom [Agent] V PP_na ('at')+acc [Recipient])	NEG	complaining, low pitch, repetitive
**brundati 'grumble'**	...svi nešto brundaju.<br>'...everyone is grumbling something=about something.'	animal	META	NP_nom [Agent] V NP_acc [Theme]	NP_nom [Agent] V	NP_nom [Agent] V PP_na ('at')+acc [Recipient])	NEG	complaining, low pitch, repetitive
**brundati 'grumble'**	Ta molim te, ne brundaj mi više.<br>'Please, stop grumbling to me=at me.'	animal	META	NP_nom [Agent] V NP_dat [Recipient]	NP_nom [Agent] V	NP_nom [Agent] V PP_na ('at')+acc [Recipient])	NEG	complaining, low pitch, repetitive
**brundati 'grumble'**	I onda naše novine i naši vajni nacionalistički akademici brundaju na predsjednika republike što se u Trstu klanja mitu o 300.000 jadranskih talijanskih ezula.<br>'And then our newspapers and our so-called nationalist academics grumble at the president of the republic for bowing to the myth of the 300,000 Adriatic Italian exiles in Trieste.'	animal	META	NP_nom [Agent] V PP_na ('at')+acc [Patient]/[Recipient]	NP_nom [Agent] V	NP_nom [Agent] V PP_na ('at')+acc [Recipient])	NEG	complaining, low pitch, repetitive

VERB	EXAMPLE	SOURCE DOMAIN	METAPHOR/METONYMY	VALENCY FRAME OF THE EXAMPLE	VALENCY FRAME IN THE SOURCE DOMAIN	VALENCY FRAME IN THE SOURCE DOMAIN	EMOTIONAL VALENCY	HIGHLIGHTED MAPPING
**brundati 'grumble'**	Tražila je od prisutnih da potvrde njezinu pobjedu, ali te su se ulizice uskomešale i brundale o nekakvoj neregularnosti. 'She asked those present to confirm her victory, but those sycophants stirred up and grumbled about some irregularity.'	animal	META	NP_nom [Agent] V PP_o ('about')+loc [Theme]	NP_nom [Agent] V	NP_nom [Agent] V PP_na ('at')+acc [Recipient])	NEG	complaining, low pitch, repetitive
**brundati 'grumble'**	...nemojte brundati protiv školskog sustava i nastavnika. '...don't grumble=complain about the school system and teachers.'	animal	META	NP_nom [Agent] V PP_protiv ('against')+gen [Theme]	NP_nom [Agent] V	NP_nom [Agent] V PP_na ('at')+acc [Recipient])	NEG	complaining, low pitch, repetitive
**brundati 'grumble'**	Znao je brundati : Moj tata, neće to još ovako dugo. 'He used to grumble: "My dad, this won't last much longer."'	animal	META	NP_nom [Agent] V QUOT [Theme]	NP_nom [Agent] V	NP_nom [Agent] V PP_na ('at')+acc [Recipient])	NEG	complaining, low pitch, repetitive
**cičati 'squeak'**	...svi bi cičali da je to novi trend. 'Everyone would be squeaking that=about it being the new trend.'	animal	META	NP_nom [Agent] V da ('that')_CP [Theme]	NP_nom [Agent] V		NEG	high pitch, irritating
**cičati 'squeak'**	Ljubi me u obraze i počinje cičati o svemu i svačemu. 'She kisses me on the cheeks and starts squeaking=whining about everything and anything.'	animal	META	NP_nom [Agent] V PP_o ('about')+loc [Theme]	NP_nom [Agent] V		NEG	high pitch, irritating
**cičati 'squeak'**	Luđak - ciči ona tankim glasićem. '"Madman!" she squeaks in her high-pitched voice.'	animal	META	NP_nom [Agent] V QUOT [Theme]	NP_nom [Agent] V		NEG	high pitch, irritating
**cijukati 'squeak'**	Tata je cijukao : Ali šta ja tu mogu, ženska glavo? 'Dad squeaked: "But what can I do about it, woman?"'	animal	META	NP_nom [Agent] V QUOT [Theme]	NP_nom [Agent] V		NEG	high pitch, loud
**cijuknuti 'squeak'**	Sad se vidi velik brod! cijukne Tonka. '"Now you can see the big ship!" Tonka squeaks.'	animal	META	NP_nom [Agent] V QUOT [Theme]	NP_nom [Agent] V		NEUTRAL	high pitch, loud, surprise
**ciknuti 'squeak'**	Znamo, već vidimo nekog nabrijanog forumaša koji će ciknuti da nije Hrvatska nego Slovenija napravila od tih vukojebina svoje Kosovo. 'We already know some pumped-up forum user will squeak that it wasn't Croatia, but Slovenia, that made its Kosovo out of these godforsaken places.'	animal	META	NP_nom [Agent] V da ('that')_CP [Theme]	NP_nom [Agent] V		NEG	high pitch, irritating
**ciknuti 'squeak'**	Tanja sokirano cikne : - Pa kako? 'Tanja, shocked, squeaks: "But how?"'	animal	META	NP_nom [Agent] V QUOT [Theme]	NP_nom [Agent] V		NEUTRAL	high pitch, loudm surprise
**cviliti 'whine'**	Ne, ne mislim ni kukati ni cviliti... to ne radim ni u svom pravom dnevniku... niti ću pričati što me muči, to je moja privatna stvar... neki ionako znaju previše. 'No, I don't intend to whine or complain... I don't do that even in my real diary... nor will I talk about what's bothering me, that's my private matter... some people already know too much.'	animal	META	NP_nom [Agent] V	NP_nom [Agent] V		NEG	complaining, high pitch, irritating
**cvrčati 'chirp'**	Neki su stalno šuškali čipsima, štapićima i ostalim grickalicama, neki su slušali glazbu, neki spavali, a neki stalno cvrčali kako priroda " zove ".	animal	META	NP_nom [Agent] V kako ('that')_CP [Theme]	NP_nom [Agent] V		NEG	irritating
**cvrčati 'chirp'**	Već će sutra cvrčat drugu priču. 'Tomorrow, they'll be chirping a different story.'	animal	META	NP_nom [Agent] V NP_acc [Theme]	NP_nom [Agent] V		NEG	irritating
**cvrkutati 'tweet, chirp'**	Zato Banderas danas cvrkuće da neće voda poskupjeti, jer je već poskupjela, ili su promijenili način obračuna. 'That's why Banderas is chirping today that the water won't get more expensive, because it already has, or they've changed the way it's calculated.'	animal	META	NP_nom [Agent] V da ('that')_CP [Theme]	NP_nom [Agent] V		NEGATIVE (irony)	cheerful, pleasant
**cvrkutati 'tweet, chirp'**	I kako sad vi mene hoćete nagovoriti da ja cvrkućem o nečemu što je samo vaša iluzija? 'And now, how do you expect me to be convinced to chirp about something that's just your illusion?'	animal	META	NP_nom [Agent] V PP_o ('about')+loc [Theme]	NP_nom [Agent] V		POSITIVE	cheerful, pleasant

VERB	EXAMPLE	SOURCE DOMAIN	METAPHOR/METONYMY	VALENCY FRAME OF THE EXAMPLE	VALENCY FRAME IN THE SOURCE DOMAIN	VALENCY FRAME IN THE SOURCE DOMAIN	EMOTIONAL VALENCY	HIGHLIGHTED MAPPING
**cvrkutati 'tweet, chirp'**	Ana je cvrkutala o noći, o muzici, ugasila je svijeće da bi mi pokazala puno polje krijesnica koje se steralo pred hotelom.<br>'Ana chirped about the night, about the music, then she turned off the candles to show us the vast field of fireflies that had gathered in front of the hotel.'	animal	META	NP_nom [Agent] V PP_o ('about')+loc [Theme]	NP_nom [Agent] V		POSITIVE	cheerful, pleasant
**cvrkutati 'tweet, chirp'**	Ja ću zalijevati papriku veselo je cvrkutala dok ju je majka odvraćala i nagovarala da nastavi igru.<br>'"I will water the pepper," she chirped happily as her mother tried to distract her and persuade her to continue playing.'	animal	META	NP_nom [Agent] V QUOT [Theme]	NP_nom [Agent] V		POSITIVE	cheerful, pleasant
**ćurlikati 'chirp'**	...počnu ćurlikati kako im je bed.<br>'...they start chirping how they're upset=about how they're upset.'	animal	META	NP_nom [Agent] V kako ('that')_CP [Theme]	NP_nom [Agent] V		NEG	collective, irritating
**frknuti 'hiss'**	Osjetljivom razdoblju - frkne Tihana.<br>'"In a sensitive period," Tihana hisses.'	animal	META	NP_nom [Agent] V QUOT [Theme]	NP_nom [Agent] V		NEG	angry
**frknuti 'hiss'**	Ja ne dozvoljavam takva sranja - frknuo je Stjepan Mesić na novinarku Ivanu Brkić nakon što je ljutito prekinuo intervju za RTL-televiziju, ustao se i otišao.<br>'"I don't tolerate that kind of crap," Stjepan Mesić hissed at journalist Ivana Brkić after angrily interrupting the interview for RTL Television, getting up, and leaving.'	animal	META	NP_nom [Agent] V QUOT [Theme] PP_na ('at')+acc [Patient]	NP_nom [Agent] V	NP_nom [Agent] V PP_na ('at')+acc [Recipient]	NEG	angry
**frktati 'hiss'**	A ženske kolegice bi frktale sa strane da je ovakva i onakva.<br>'And the female colleagues would hiss on the side, that she's this way or that way=saying she's this way or that way.'	animal	META	NP_nom [Agent] V da ('that')_CP [Theme]	NP_nom [Agent] V	NP_nom [Agent] V PP_na ('at')+acc [Recipient]	NEG	contempt
**frktati 'hiss'**	Prezrivo sam frktao na nju: " Mama, gledaj ti svoja posla."<br>'I contemptuously hissed at her: "Mom, mind your own business."'	animal	META	NP_nom [Agent] V QUOT [Theme] PP_na ('at')+acc [Patient]	NP_nom [Agent] V	NP_nom [Agent] V PP_na ('at')+acc [Recipient]	NEG	angry, contempt
**gakati 'quak'**	A zasto onda kada se god spomene podatak da se u nas ne radja dovoljno djece sve koke odjednom pocnu histericno gakati o "strojevima za radjanje"?<br>'And why is it that whenever the fact comes up that not enough children are being born here, all the hens suddenly start hysterically quaking=clucking about "baby-making machines"?'	animal	META	NP_nom [Agent] V PP_o ('about')+loc [Theme]	NP_nom [Agent] V		NEG	irritating, collective
**gaknuti 'quak'**	Iz očaja, gaknuh im razočaranim gakom neku psovku.<br>'Out of despair, I squawked a curse at them.'	animal	META	NP_nom [Agent] V NP_acc [Theme] NP_dat [Recipient]	NP_nom [Agent] V		NEG	loudness?
**graknuti 'caw'**	Sada će pobornici guranja novinarske nepravde graknuti kako je u ovim slučajevima sud pogriješio, bio nepravedan.<br>'Now, the supporters of pushing journalistic injustice will caw=grunt that the court was wrong in these cases, that it was unfair.'	animal	META	NP_nom [Agent] V kako ('that')_CP [Theme]	NP_nom [Agent] V		NEG	loudness, irritating
**graknuti 'caw'**	...sljedeći dan uvijek isti novinari i političari graknu istu priču…<br>'...the next day, the same journalists and politicians always caw=grunt the same story…'	animal	META	NP_nom [Agent] V NP_acc [Theme]	NP_nom [Agent] V		NEG	irritating
**graknuti 'caw'**	Tada svi graknuše na nj, ali on nije bio kriv.<br>'Then they all cawed at him, but he wasn't guilty.'	animal	META	NP_nom [Agent] V PP_na ('at')+acc [Patient]/[Recipient]	NP_nom [Agent] V		NEG	loud, collective, agressive
**graknuti 'caw'**	To su bili svi oni koji odmah graknu na svaku novost te ju odsuđuju, mada se nisu dobro ni promislili.<br>'These were all those who immediately caw at every new development and condemn it, even though they haven't thought it through properly.'	animal	META	NP_nom [Agent] V PP_na ('at')+acc [Theme]	NP_nom [Agent] V		NEG	loud, agressive

VERB	EXAMPLE	SOURCE DOMAIN	METAPHOR/METONYMY	VALENCY FRAME OF THE EXAMPLE	VALENCY FRAME IN THE SOURCE DOMAIN	VALENCY FRAME IN THE SOURCE DOMAIN	EMOTIONAL VALENCY	HIGHLIGHTED MAPPING
**graknuti 'caw'**	...pa sam našao shodnim da i ja graknem o toj temi. '...so I found it fitting to caw about that topic as well.'	animal	META/METO	NP_nom [Agent] V PP_o ('about')+loc [Theme]	NP_nom [Agent] V		NEG	collective, loud
**graknuti 'caw'**	Nedavno je najglasnije graknuo protiv Vladine odluke o poskupljenju trajektnih karata i ukidanju broja linija. 'Recently, he cawed the loudest against the government's decision to increase ferry ticket prices and reduce the number of routes.'	animal	META/METO	NP_nom [Agent] V PP_protiv ('against')+gen [Theme]	NP_nom [Agent] V		NEG	loud
**graknuti 'caw'**	I inače nastavio potkupljivati (a i mi davali koliko se imalo), kada su npr. graknuli za pušenje u zatvorenom prostoru ili objavili (više puta) da se bliži fajrunt. 'And he continued to bribe (and we gave as much as we could), whenever, for example, they cawed for= about smoking indoors or repeatedly announced that closing time was approaching.'	animal	META/METO	NP_nom [Agent] V PP_za ('for')+acc [Theme]	NP_nom [Agent] V		NEG	loud
**graknuti 'caw'**	S taksijem iz Zagreba u Imotski? – graknuše desetorica odjednom. 'A taxi from Zagreb to Imotski? – they all cawed at once.'	animal	META	NP_nom [Agent] V QUOT [Theme]	NP_nom [Agent] V		NEG	loud, collective, agressive
**graknuti 'caw'**	Moj sin pijanac! Moj Teodoro ništarija! Čovječe, pazite, što govorite! graknu Barbara na starca. '"My son, a drunkard! My Teodoro, a good-for-nothing! Man, be careful what you say!" Barbara cawed at the old man.'	animal	META	NP_nom [Agent] V QUOT [Theme] PP_na ('at')+acc [Patient]/[Recipient]	NP_nom [Agent] V		NEG	loud, agressive
**graktati 'caw'**	A svi ste graktali na starog Jordana da trabunja gluposti. 'And you all cawed at old Jordan that he is babbling nonsense=for babbling nonsense.'	animal	META	NP_nom [Agent] V da ('that')_CP [Theme] NP_na ('at')+acc [Patient]/[Recipient]	NP_nom [Agent] V		NEG	collective, loud, agressive
**graktati 'caw'**	Koliko u veljači oba su graktali kako je to jedno izvanredno demokratsko dostignuće. 'In February, both of them cawed how this is an extraordinary democratic achievement=about how this is an extraordinary democratic achievement.'	animal	META	NP_nom [Agent] V kako ('that')_CP [Theme]	NP_nom [Agent] V		NEG	irritating, collective
**graktati 'caw'**	Pisci i općinstvo, sve to grakće jedno na drugo, pisci na općinstvo što ne kupuje, a ovo na pisce što il' ne pišu ili ako što ispišu, to ne vrijedi mnogo. 'Writers and the public, all they do is caw at each other – writers caw at the public for not buying, and the public caws at the writers for either not writing, or if they do, what they write isn't worth much.'	animal	META	NP_nom [Agent] V PP_na ('at')+acc [Patient]/[Recipient]	NP_nom [Agent] V		NEG	agressive, irritating
**graktati 'caw'**	Da nije tako već bi graktali protiv njega kao što su svojedobno to radili protiv Mečiara u Slovačkoj. 'If it weren't like that, they would have cawed against him, just like they once did against Mečiar in Slovakia.'	animal	META	NP_nom [Agent] V PP_protiv ('against')+gen [Theme]	NP_nom [Agent] V		NEG	irrittaing, agressive, collective
**graktati 'caw'**	Dakle, oni su cijelo vrijeme graktali : "IE je smece". 'So, they were constantly cawing: "IE is garbage".'	animal	META	NP_nom [Agent] V QUOT [Theme]	NP_nom [Agent] V		NEG	irritating, collective
**groktati 'grunt'**	Ljudi ne žele da budu poistovjećeni sa politikom onih koji tamo sjede, žderu, piju i grokću za govornicom. 'People don't want to be associated with the politics of those who sit there, eat, drink, and grunt at the podium.'	animal	META	NP_nom [Agent] V	NP_nom [Agent] V		NEG	greed, dirtiness (immorality)

VERB	EXAMPLE	SOURCE DOMAIN	METAPHOR/METONYMY	VALENCY FRAME OF THE EXAMPLE	VALENCY FRAME IN THE SOURCE DOMAIN	VALENCY FRAME IN THE SOURCE DOMAIN	EMOTIONAL VALENCY	HIGHLIGHTED MAPPING
**gugutati 'coo'**	...jer iako svi guguču da imaju iste ponude neznaju svi isto potražiti. '...because even though everyone coos that they have the same offers, not everyone knows how to seek the same things.'	animal	META	NP_nom [Agent] V da ('that')_CP [Theme]	NP_nom [Agent] V		NEG	irritating, stupid
**gugutati 'coo'**	Drugo - nerealne gluposti da Hrvatska može proizvodnju struje prebaciti na solarne, vjetroelektrane, biomasu su plod izmišljanja " zelenih " udruga koje su financirane izvana da guguču gluposti. 'Another thing – the unrealistic nonsense that Croatia could switch its electricity production to solar, wind power, and biomass is the result of the imagination of "green" associations funded from abroad to coo nonsense.'	animal	META	NP_nom [Agent] V NP_acc [Theme]	NP_nom [Agent] V		NEG	irritating, stupid
**gukati 'coo'**	Četiri dana je šjor Medardo ležao u agoniji i zatim umro bez većih smetnja za okolinu, dosta jednostavno, gukajući nešto nerazumljivo, kao bolesni golub pod strehom kad beznadno zavuče glavu pod krilo i izdahne. 'For four days, Mister Medardo lay in agony and then died without much disturbance to those around him, quite simply, cooing=muttering something unintelligible, like a sick pigeon under the eaves when it hopelessly tucks its head under its wing and breathes its last.'	animal	META	NP_nom [Agent] V NP_acc [Theme]	NP_nom [Agent] V		NEUTRAL	faintly
**gukati 'coo'**	Kaže se da o ljubavi svi guču i pjevaju, šapuću i viču… 'It's said that about love, everyone coos and sings, whispers and shouts…'	animal	META	NP_nom [Agent] V PP_o ('about')+loc [Theme]	NP_nom [Agent] V		POSITIVE	gently
**gukati 'coo'**	Uzalud je Nuša gukala Matanu: " Što će ti spišulje, sunce moje? 'Nuša cooed to Matan in vain: "Why do you need those little women, my dear?"'	animal	META	NP_nom [Agent] V QUOT [Theme] NP_dat [Recipient]	NP_nom [Agent] V		POSITIVE	gently, alluring
**guknuti 'coo'**	A sad - gukni neku njihovu tajnu, ovo je pravi topik za to. 'And now – coo=spill one of their secrets, this is the perfect topic for that.'	animal	META/METO	NP_nom [Agent] V NP_acc [Theme]	NP_nom [Agent] V		NEUTRAL?	
**guknuti 'coo'**	Ajde gukni mi zelju. 'Come on, coo=tell me your wish.'	animal	META/METO	NP_nom [Agent] V NP_acc [Theme] NP_dat [Recipient]	NP_nom [Agent] V		NEUTRAL?	
**guknuti 'coo'**	Nikako da gukne o tome zašto nju smeta da se homo-parovi žene. 'She just won't coo=spill why it bothers her that gay couples get married.'	animal	META/METO	NP_nom [Agent] V PP_o ('about')+loc [Theme]	NP_nom [Agent] V		NEUTRAL?	
**guknuti 'coo'**	...isto što je idiotski nazivat danas Njemce nacistima, ili svakoga tko gukne protiv židovske doktrine antisemitom. '...just like it's idiotic to call Germans Nazis today, or anyone who cooes=speaks out against the Jewish doctrine an anti-Semite.'	animal	META/METO	NP_nom [Agent] V PP_protiv ('against')+gen [Theme]	NP_nom [Agent] V		NEUTRAL?	
**guknuti 'coo'**	Novi sluga, anđele - guknu čovječac. '"New servant, angel," the little man cooed.'	animal	META	NP_nom [Agent] V QUOT [Theme]	NP_nom [Agent] V		NEUTRAL?	
**guknuti 'coo'**	ajde gukni sto sad hoces… 'Come on, coo=spill what you want now…'	animal	META/METO	NP_nom [Agent] V što_CP [Theme]	NP_nom [Agent] V		NEUTRAL?	
**hukati 'roar'**	O bože, o moj dobri bože - hukala je Valpurga priređujući Lucijin krevet. 'Oh God, oh my good God, Valpurga roared, while preparing Lucie's bed.'	animal	META	NP_nom [Agent] V QUOT [Theme]	NP_nom [Agent] V		NEG	repetitive, fear, sadness
**kljucati 'cluck'**	- Nitko im ne kljuca kad će se oženiti i roditi. 'No one clucks=nags them about when they will get married and have children.'	animal	META	NP_nom [Agent] V kad_CP [Theme] NP_dat [Patient]/[Recipient]	NP_nom [Agent] V NP_acc [Theme]	NP_nom [Agent] V NP_acc [Theme]	NEG	repetitive, irritating, unpleasant

VERB	EXAMPLE	SOURCE DOMAIN	METAPHOR/METONYMY	VALENCY FRAME OF THE EXAMPLE	VALENCY FRAME IN THE SOURCE DOMAIN	VALENCY FRAME IN THE SOURCE DOMAIN	EMOTIONAL VALENCY	HIGHLIGHTED MAPPING
**kljucati 'cluck'**	On je stalno htio jesti, ali mu nisam smjela dati, a svi su kljucali sa strane kako će se udebljati.<br>'He constantly wanted to eat, but I couldn't let him, and everyone was clucking=nagging on the side about how he would get fat.'	animal	META	NP_nom [Agent] V kako ('that')_CP [Theme]	NP_nom [Agent] V NP_acc [Theme]	NP_nom [Agent] V NP_acc [Theme]	NEG	repetitive, irritating, unpleasant
**kokodakati 'cluck'**	A najgore je kad mi ovi doma počnu kokodakat kako trebam uhvatit knjigu i dignut ocjene.<br>'And the worst is when these people at home start clucking how I need to grab a book and improve my grades=about how I need to grab a book and improve my grades.'	animal	META	NP_nom [Agent] V kako ('that')_CP [Theme] NP_dat [Recipient]	NP_nom [Agent] V		NEG	irritating, collective, repetitive
**kokodakati 'cluck'**	A danas imamo mnoštvo partija a sve kokodaču istu priču.<br>'And today we have a multitude of parties, and they're all clucking the same story.'	animal	META	NP_nom [Agent] V NP_acc [Theme]	NP_nom [Agent] V		NEG	irritating, collective, stupid
**kokodakati 'cluck'**	...a ne vi samo gledate preko Neretve i kokodačete na Hrvate.<br>'...and you just look across the Neretva and cluck at the Croats.'	animal	META	NP_nom [Agent] V PP_na ('at')+acc [Patient]/[Recipient]	NP_nom [Agent] V		NEG	irritating, collective, repetitive
**kokodakati 'cluck'**	Kokodačite o svom proizvodu…<br>'You're clucking about your product…'	animal	META	NP_nom [Agent] V PP_o ('about')+loc [Theme]	NP_nom [Agent] V		NEG	irritating, collective, stupid
**kokodakati 'cluck'**	...ako se on borio za to da vi imate pravo kokodakati protiv Hrvatske, onda i ja imam pravo zalagati se za nju u pravom, hrvatskom smislu.<br>'...if he fought for you to have the right to cluck against Croatia, then I also have the right to stand up for her in the true, Croatian sense.'	animal	META	NP_nom [Agent] V PP_protiv ('against')+gen [Theme]	NP_nom [Agent] V		NEG	irritating, collective, repetitive
**kokodaknuti 'cluck'**	A vi kokodaknule da ga nema.<br>'And you all clucked that he's gone.'	animal	META	NP_nom [Agent] V da ('that')_CP [Theme]	NP_nom [Agent] V		NEG	loud, exaggerating importance
**kokodaknuti 'cluck'**	Koka bude kokodaknula kako je riječ o statističkoj grešci.<br>'The hen will cluck that it's a statistical error.'	animal	META	NP_nom [Agent] V kako ('that')_CP [Theme]	NP_nom [Agent] V		NEG	loud, exaggerating importance
**kokodaknuti 'cluck'**	Što vi možete kokodaknut na ovu temu?<br>'What can you cluck about this topic?'	animal	META/METO	NP_nom [Agent] V PP_na ('at')+acc [Theme]	NP_nom [Agent] V		NEG	
**kokodaknuti 'cluck'**	"Dodji s nama dernecit u subotu.", kokodaknula sam.<br>'"Come party with us on Saturday," I clucked.'	animal	META	NP_nom [Agent] V QUOT [Theme]	NP_nom [Agent] V		NEUTRAL?	loud
**kr(ij)eštati 'screech'**	Ja gledah sve EU i svjetske TV, jedino što puštaju su babe sa Dolca koje krešte kako su svi lopovi i da su sve pokrali, a baba u krznenom kaputu.<br>'I watched all the EU and world TV, and the only thing they're airing are old women from Dolac screeching that everyone is a thief and that they've stolen everything, while the old woman is in a fur coat.'	animal	META	NP_nom [Agent] V kako ('that')_CP [Theme]	NP_nom [Agent] V		NEG	loud, irritating
**kr(ij)eštati 'screech'**	...kreštao na jednu damu od 80 godina i zabranjivao joj da iznese vlastito mišljenje.<br>'...he screeched at an 80-year-old lady and forbade her from expressing her own opinion.'	animal	META	NP_nom [Agent] V PP_na ('at')+acc [Patient]/[Recipient]	NP_nom [Agent] V		NEG	loud, agressive
**kr(ij)eštati 'screech'**	Ona kreštava crvenokosa političakra navodno liberalne partije ugnijezdila se u vladi i više ne krešti o pravima žena.<br>'That shrill red-haired politician from the supposedly liberal party has nested in the government and no longer screeches/caws about women's rights.'	animal	META	NP_nom [Agent] V PP_o ('about')+loc [Theme]	NP_nom [Agent] V		NEG	irritating

VERB	EXAMPLE	SOURCE DOMAIN	METAPHOR/METONYMY	VALENCY FRAME OF THE EXAMPLE	VALENCY FRAME IN THE SOURCE DOMAIN	VALENCY FRAME IN THE SOURCE DOMAIN	EMOTIONAL VALENCY	HIGHLIGHTED MAPPING
**kr(ij)eštati 'screech'**	I zasjala je tako još više Ruža, i stekla status puno bolje osobe od svih, a to znači da su svi bukači protiv nje prepoznati kao politička bagra, polusvijet, ma kako god i koliko god nastavljali kriještati protiv te od naroda izabrane žene. 'And so Ruža shone even brighter, gaining the status of a much better person than anyone else, which meant that all the noise-makers against her were recognized as political scum, the underworld, no matter how much or how long they continued to screech against this woman chosen by the people.'	animal	META	NP_nom [Agent] V PP_protiv ('against')+gen [Theme]	NP_nom [Agent] V		NEG	loud, irritating, collective
**kr(ij)eštati 'screech'**	Već te čujem kako kriještiš : - Ne daj mu, ionako će sve zapiti. 'I can already hear you screeching: "Don't give it to him, he'll drink it all away anyway."'	animal	META	NP_nom [Agent] V QUOT [Theme]	NP_nom [Agent] V		NEG	loud, irritating
**kreketati 'croak'**	Mi krekećemo kako je UEFA licemjerna i bahata istovremeno za probleme u nogometu optužujući jedni druge. 'We croak that UEFA is hypocritical and arrogant, while simultaneously blaming each other for the problems in football.'	animal	META	NP_nom [Agent] V kako ('that')_CP [Theme]	NP_nom [Agent] V		NEG	collective, repetitive, irritating, vainly
**kreketati 'croak'**	Onda ćete vi kreketati nešto o tome kako se u Srbiji gaze nečija prava ZAJAMČENA USTAVOM. 'Then you'll croak something about how in Serbia, people's rights GUARANTEED BY THE CONSTITUTION are being trampled.'	animal	META	NP_nom [Agent] V NP_acc [Theme]	NP_nom [Agent] V		NEG	collective, repetitive, irritating, in vain
**kreketati 'croak'**	U političkoj bari opet se krekeće o svemu i ničemu. 'In the political swamp, they're croaking about everything and nothing again.'	animal	META	NP_nom [Agent] V PP_o ('about')+loc [Theme]	NP_nom [Agent] V		NEG	collective, repetitive, irritating, in vain
**kričati 'screech'**	Djeca su iz busa kričala da im otvorim vrata. 'The kids were screeching=shouting from the bus for me that I open the door= to open the door.'	animal	META	NP_nom [Agent] V da ('that')_CP [Theme]	NP_nom [Agent] V		NEG	loud, irritating
**kričati 'screech'**	I on se zauzme za svoju župljane, dogovorivši da će svi pustiti bradu i brkove, to će potrajati, a da im žene ne bi kričale, neka im se dade malo veći honorar. 'And he stands up for his parishioners, agreeing that everyone will grow beards and mustaches, which will take some time, and so that their wives don't screech=yell at them that they should be given a slightly higher fee.'	animal	META	NP_nom [Agent] V neka_CP [Theme] NP_dat [Recipient]	NP_nom [Agent] V		NEG	loud, irritating
**kričati 'screech'**	I on se umiješa u crkveno ustrojstvo, kao da baš taj isti sud i okoliš iz kojeg potječe ne kriči o rastavljenosti Crkve i države. 'And he meddles in the church's structure, as if that very same court and environment from which he comes doesn't schreech=shout about the separation of church and state.'	animal	META	NP_nom [Agent] V PP_o ('about')+loc [Theme]	NP_nom [Agent] V		NEG	loud, irritating
**kričati 'screech'**	Kako to? - stade kričati Trepetljika, a nos kano da joj se uzdigao uvis… 'How's that? – Trepetljika started screeching=shouting, and her nose practically rising up in the air…'	animal	META	NP_nom [Agent] V QUOT [Theme]	NP_nom [Agent] V		NEG	loud, irritating
**kričati 'screech'**	Sviraj ti - kriči na glasovirača, baca i njemu novac i pilji u ono nago plešuće kolo, uživa u razvratu cura… '"Play" she screeches=screams at the pianist, throwing him money and staring at the naked dancing circle, enjoying the girls' decadence…'	animal	META	NP_nom [Agent] V QUOT [Theme] PP_na ('at')+acc [Patient]/[Recipient]	NP_nom [Agent] V		NEG	loud, irritating, agressive
**kukati 'cuckoo; whine, lament'**	Dakle, cure i dečki, nemojte kukati (sebi ili drugima) da izgledate staro i umorno. 'So, girls and boys, don't cuckoo=whine (to yourselves or others) that you look old and tired.'	animal	META	NP_nom [Agent] V da ('that')_CP [Theme] NP_dat [Recipient]	NP_nom [Agent] V		NEG	repetitive, irritating, complaining

VERB	EXAMPLE	SOURCE DOMAIN	METAPHOR/METONYMY	VALENCY FRAME OF THE EXAMPLE	VALENCY FRAME IN THE SOURCE DOMAIN	VALENCY FRAME IN THE SOURCE DOMAIN	EMOTIONAL VALENCY	HIGHLIGHTED MAPPING
**kukati 'cuckoo; whine, lament'**	Teatralni Šiša, koji je više puta najavljivao svoje povlačenje, prvo se malo promišljao i predomišljao, da bi zatim javno kukao svoje žalopojke i na kraju ih sve prešišao.<br>'The theatrical Šiša, who had repeatedly announced his retirement, first thought it over and reconsidered, then publicly cuckooed=whined his complaints, and in the end, outdid them all.'	animal	META	NP_nom [Agent] V NP_acc [Theme]	NP_nom [Agent] V		NEG	repetitive, irritating, complaining
**kukati 'cuckoo; whine, lament'**	...pogotovo ovi što kukaju o (svojim) malim plaćama.<br>'...especially those who cuckoo=whine about (their) low salaries.'	animal	META	NP_nom [Agent] V PP_o ('about')+loc [Theme]	NP_nom [Agent] V		NEG	repetitive, irritating, complaining
**kukati 'cuckoo; whine, lament'**	Ne razumijem zašto kukaš protiv antikorupcijskog programa??<br>'I don't understand why you're cuckooing=whining against the anti-corruption program??'	animal	META	NP_nom [Agent] V PP_protiv ('against')+gen [Theme]	NP_nom [Agent] V		NEG	repetitive, irritating, complaining
**kukati 'cuckoo; whine, lament'**	Kukamo za radnim mjestima…<br>'We're cuckooing=whining after=about job opportunities.'	animal	META	NP_nom [Agent] V PP_za ('for')+inst [Theme]	NP_nom [Agent] V		NEG	repetitive, irritating, complaining
**kukurikati 'crow'**	Ovaj put su kukurikali, pa su mediji ostali prikraćeni za njihova suosjećajna lica.<br>'This time they crowed, and the media were deprived of their sympathetic faces.'	animal	META	NP_nom [Agent] V	NP_nom [Agent] V		NEG	irritating, in vain
**kukuriknuti 'crow'**	Ovih je dana kukuriknuo premijeru (…) da je vrijeme za zaustavljanje radova na podravskom ipsilonu jer tako je križevčanima obećano.<br>'Recently, he crowed to the prime minister that it's time to stop the construction of the Podravina Y highway because that's what was promised to the people of Križevci.'	animal	META	NP_nom [Agent] V da ('that')_CP [Theme] NP_dat [Recipient]	NP_nom [Agent] V		NEG	loud, sudden, irritating
**kukuriknuti 'crow'**	Predsjednik vlade Milanović sa svojim kukurikavcima, trebao bi kukuriknuti istinu, jer istina oslobađa.<br>'Prime Minister Milanović, along with his roosters, should crow the truth, because the truth sets you free.'	animal	META/METO	NP_nom [Agent] V NP_acc [Theme]	NP_nom [Agent] V		NEUTRAL	for everybody to hear
**kukuriknuti 'crow'**	Pa Bajić nije kukuriknuo ništa o pljački drugova iz SDPa u aferi DAIMLER.<br>'But Bajić didn't crow anything about the theft by his comrades from the SDP in the DAIMLER scandal.'	animal	META/METO	NP_nom [Agent] V NP_acc [Theme]	NP_nom [Agent] V		NEUTRAL	for everybody to hear
**kukuriknuti 'crow'**	Ajde, kukuriknite opet na patriotizam…<br>'Come on, crow again at=about patriotism…'	animal	META/METO	NP_nom [Agent] V PP_na ('at')+acc [Theme]	NP_nom [Agent] V		NEG	irritating, for everybody to hear
**kukuriknuti 'crow'**	One koji godinama nisu željeli "kukuriknuti" o kriminalu i korupciji u vrhovima vlasti.<br>'Those who for years refused to "crow" about the crime and corruption at the top of the government.'	animal	META/METO	NP_nom [Agent] V PP_o ('about')+loc [Theme]	NP_nom [Agent] V		NEUTRAL	for everybody to hear
**kvakati 'quak'**	Skupit će se šačica ljudi kojima je "pomoglo" i kvakat će okolo da stvar funkcionira.<br>'A handful of people for whom it "helped" will gather together and quack= drone that the thing works.'	animal	META	NP_nom [Agent] V da ('that')_CP [Theme]	NP_nom [Agent] V		NEG	irrittaing, collective, stupid?
**kvakati 'quak'**	Pa evo mi moja pačja škola šapće na uho da "neopredijeljeni" nisu Hrvati, koliko god mi kvakali o tome.<br>'Well, here's my duck school whispering in my ear that the "undecided" are not Croats, no matter how much we quack about it.'	animal	META	NP_nom [Agent] V PP_o ('about')+loc [Theme]	NP_nom [Agent] V		NEG	irritating, collective

VERB	EXAMPLE	SOURCE DOMAIN	METAPHOR/METONYMY	VALENCY FRAME OF THE EXAMPLE	VALENCY FRAME IN THE SOURCE DOMAIN	VALENCY FRAME IN THE SOURCE DOMAIN	EMOTIONAL VALENCY	HIGHLIGHTED MAPPING
**kvocati 'cluck; nag'**	Cijeli tjedan kvocam šefu da mi treba obnoviti ugovor.<br>'I've been clucking=nagging my boss all week to renew my contract.	animal	META	NP_nom [Agent] V da ('that')_CP [Theme] NP_dat [Patient]/[Recipient]	NP_nom [Agent] V		NEG	irritating, agressive, complaining
**kvocati 'cluck; nag'**	I onda mi još kvocaju nad glavom da im se ustanem. A to što ja imam torbu punu knjiga nije problem.<br>'And then they're still clucking=nagging me to get up. But the fact that I have a bag full of books isn't a problem.'	animal	META	NP_nom [Agent] V da ('that')_CP [Theme] NP_dat [Patient]/[Recipient]	NP_nom [Agent] V		NEG	irritating, agressive, complaining
**kvocati 'cluck; nag'**	Pa normalno da ne trebam kvocati djetetu stalno o nekoj aktivnosti…<br>'Well, of course, I shouldn't have to cluck=nag my child constantly about some activity…'	animal	META	NP_nom [Agent] V PP_o ('about')+loc [Theme] NP_dat [Patient]/[Recipient]	NP_nom [Agent] V		NEG	irritating, agressive, complaining
**kvocati 'cluck; nag'**	I mužu doma kvocala protiv države koje ljudima onemogućuje da od poštenog rada žive.<br>'And at home, she clucked=nagged her husband against=about the state that makes it impossible for people to live off honest work.'	animal	META	NP_nom [Agent] V PP_protiv ('against')+gen [Theme] NP_dat [Patient]/[Recipient]	NP_nom [Agent] V		NEG	irritating, agressive, complaining
**lajati 'bark'**	Svi ti Leopoldi Mandeli, Neumanni, Friedjungi i Nopče, Prohaske i Pester Lloydi, Az Ešt i Neue Freie Presse lajali su bijesno i krvavo kako ta divlja slavenska balkanska plemena oru još drvenim plugovima...<br>'All those Leopold Mandels, Neumanns, Friedjungs, Nopčes, Prohaskas, Pester Lloyds, Az Esht, and Neue Freie Presse barked fiercely and bloodily about how those wild Slavic Balkan tribes still plow with wooden plows...'	animal	META	NP_nom [Agent] V kako ('that')_CP [Theme]	NP_nom [Agent] V	NP_nom [Agent] V PP_na ('at')+acc [Recipient])	NEG	agressive, harming (psych.)
**lajati 'bark'**	…da bi najbolje bilo da drži svoju gubicu i da ne laje tu sve koještarije neke bezglave i besmislene.<br>'...that it would be best for him to keep his mouth shut and not bark out all sorts of headless and meaningless nonsense.'	animal	META	NP_nom [Agent] V NP_acc [Theme]	NP_nom [Agent] V	NP_nom [Agent] V PP_na ('at')+acc [Recipient])	NEG	loud, agressive, meaningless
**lajati 'bark'**	Ta babetina, koja inače na sav svijet laje, ne može ipak nasmagati dosta riječi u hvalu majke i kćeri.<br>'That old woman, who usually barks at the whole world, still can't manage to say enough words in praise of her mother and daughter.'	animal	META	NP_nom [Agent] V PP_na ('at')+acc [Patient]/[Recipient]	NP_nom [Agent] V	NP_nom [Agent] V PP_na ('at')+acc [Recipient])	NEG	agressive, harming (psych.)
**lajati 'bark'**	Katon je još putem lajao o zakonima, o republici.<br>'Katon was still barking on about laws, about the republic.'	animal	META	NP_nom [Agent] V PP_o ('about')+loc [Theme]	NP_nom [Agent] V	NP_nom [Agent] V PP_na ('at')+acc [Recipient])	NEG	irritating, loud
**lajati 'bark'**	Čujem Paolo da laješ protiv nas, ma ćemo ti uvrnut jezik.<br>'I hear, Paolo, that you're barking against=about us, but we'll twist your tongue.'	animal	META	NP_nom [Agent] V PP_protiv ('against')+gen [Theme]	NP_nom [Agent] V	NP_nom [Agent] V PP_na ('at')+acc [Recipient])	NEG	agressive, harming (psych.)
**lajati 'bark'**	Znam ja, tko tebi laje protiv mene!<br>'I know who is barking against me (to you)!'	animal	META	NP_nom [Agent] V PP_protiv ('against')+gen [Theme] NP_dat [Recipient]	NP_nom [Agent] V	NP_nom [Agent] V PP_na ('at')+acc [Recipient])	NEG	agressive, harming (psych.)
**meketati 'bleat'**	Kumica s placa u tradicionalnoj uniformi neproničljivim dijalektom mekeće recept…<br>'The market vendor, dressed in traditional attire, bleats a recipe in an indecipherable dialect…'	animal	META	NP_nom [Agent] V NP_acc [Theme]	NP_nom [Agent] V		NEG	repetitive, irritating
**meketati 'bleat'**	...smiješno je da isti oni koji su projekt podržali bilo šutnjom bilo izravno sad mekeću protiv istog.<br>'...it's funny that the same ones who supported the project either with silence or directly are now bleating against it.'	animal	META	NP_nom [Agent] V PP_protiv ('against')+gen [Theme]	NP_nom [Agent] V		NEG	repetitive, irritating
**meketati 'bleat'**	Ma neeeee - prijetvorno mekeću prijateljice.<br>'Oh nooo – hypocritically, her friends bleat.'	animal	META	NP_nom [Agent] V QUOT [Theme]	NP_nom [Agent] V		NEG	repetitive, irritating
**meketati 'bleat'**	...a ti indoktrinirano mekećeš što su ti usadili…<br>'...and you bleat what they've instilled in you.'	animal	META	NP_nom [Agent] V što ('what')_CP [Theme]	NP_nom [Agent] V		NEG	repetitive, irritating

VERB	EXAMPLE	SOURCE DOMAIN	METAPHOR/METONYMY	VALENCY FRAME OF THE EXAMPLE	VALENCY FRAME IN THE SOURCE DOMAIN	VALENCY FRAME IN THE SOURCE DOMAIN	EMOTIONAL VALENCY	HIGHLIGHTED MAPPING
**mijaukati 'meow'**	A htio sam ići mijaukat protiv one Death of Art, opet se pogoropadila i raspojasala. 'And I wanted to go meow against that Death of Art, it's gotten worse and more unruly again.'	animal	META	NP_nom [Agent] V PP_protiv ('against')+gen [Theme]	NP_nom [Agent] V		NEG	repetitive, loud
**muknuti 'moo'**	Kome vjerovati, kad HŽ niti da mukne o toj stvari… 'Who can you trust when HŽ doesn't moo=even utter a word about that matter…'	animal	META/METO	NP_nom [Agent] V PP_o ('about')+loc [Theme]	NP_nom [Agent] V		NEUTRAL	
**muknuti 'moo'**	Ako im policija naredi da glasaju, oni glasaju, a to toliko mogli bismo i mi muknuti: „Zaaa!" 'If the police order them to vote, they vote, and we could just as well stay silent and moo=murmur, "Yeees!"'	animal	META	NP_nom [Agent] V QUOT [Theme]	NP_nom [Agent] V		NEG	collective, stupid
**njakati 'heehaw'**	Kad jedan drug zalaje da "mrzi fašističku i homofobnu zemlju", kad drugi njače da je Ustavni sud "sprdačina", pa čak i kad treći zareži kako treba razmisliti o povlačenju tužbe za razaranje i genocid, to je demokracija. 'When one friend barks that he "hates a fascist and homophobic country," another heehaws=whinnies that the Constitutional Court is a "mockery," and even when the third growls that we should reconsider the lawsuit for destruction and genocide, that's democracy.'	animal	META	NP_nom [Agent] V da ('that')_CP [Theme]	NP_nom [Agent] V		NEG	loud (meta), stupid
**njakati 'heehaw'**	Ali zato ćete spremno njakat nama Boysima kako su vam ukradene titule i kako repka nikad ne igra u Splitu. 'But then you'll eagerly heehaw=whine to us Boys about how your titles were stolen and how the national team never plays in Split.'	animal	META	NP_nom [Agent] V kako ('that')_CP [Theme] NP_dat [Recipient]	NP_nom [Agent] V		NEG	loud (meta), stupid
**njakati 'heehaw'**	Ne možeš mi njakat o 'šleperima' i ' rodijacima'… 'You can't heehaw=whine to me about 'trucks' and 'relatives'…'	animal	META	NP_nom [Agent] V PP_o ('about')+loc [Theme] NP_dat [Recipient]	NP_nom [Agent] V		NEG	loud (meta), stupid
**pijukati 'chirp, peep'**	Meni se ne događa svaki dan da pročitam tristo stranica (ili, makar, dvjestoosamdesetpet), a da na prvoj, desetoj ili stotoj ne počnem cičati kao da me kolju lošim pisanjem, da ne počnem pijukati kako je pisac kreten i ne počnem vjernom si olovčicom bilježiti sve stranice na kojima je dotični kreten počinio zločine protiv literature i moga ukusa. 'It doesn't happen every day that I read three hundred pages (or, at least, two hundred eighty-five) without starting to squeak as if I were being tortured by bad writing, without beginning to chirp that the author is an idiot, and without faithfully marking with my pencil every page where the said idiot committed crimes against literature and my taste.'	animal	META	NP_nom [Agent] V kako ('that')_CP [Theme]	NP_nom [Agent] V		NEUTRAL	loud
**presti 'purr'**	...još malo na Mediteranski brevijar i poslušajmo Matvejevića kako prede o brodu. '...let's take a little more of the Mediterranean breviary and listen to Matvejević purr about the ship.'	animal	META	NP_nom [Agent] V PP_o ('about')+loc [Theme]	NP_nom [Agent] V		NEG	repetitive, boring
**presti 'purr'**	Mmmm - počela je zadovoljno presti čim je osjetila tu veliku izbočinu. 'Mmmm – she started purring contentedly as soon as she felt that large bump.'	animal	META	NP_nom [Agent] V QUOT [Theme]	NP_nom [Agent] V		POSITIVE	pleasure
**prosiktati 'hiss'**	Mislila sam im u brk prosiktat da si ovakvu EU dobrodošlicu mogu sebi na čelo zalijepit. 'I was thinking of hissing in their face that they can stick this kind of EU welcome on their foreheads.'	animal	META	NP_nom [Agent] V da ('that')_CP [Theme] NP_dat [Patient]/[Recipient]	NP_nom [Agent] V		NEG	agressive, angry

VERB	EXAMPLE	SOURCE DOMAIN	METAPHOR/METONYMY	VALENCY FRAME OF THE EXAMPLE	VALENCY FRAME IN THE SOURCE DOMAIN	VALENCY FRAME IN THE SOURCE DOMAIN	EMOTIONAL VALENCY	HIGHLIGHTED MAPPING
**prosiktati 'hiss'**	Nakon što je bijesno prosiktao svoju žalbu, sjeo je na ležaj te, mršteći se poput nadurena djeteta, čekao da mu u vene injektiraju smrtonosni koktel.	animal	META	NP_nom [Agent] V NP_acc [Theme]	NP_nom [Agent] V		NEG	agressive, angry
**prosiktati 'hiss'**	Ona se prestala hihotati i prosiktala: - Silazi s mene.<br>'She stopped giggling and hissed: "Get off me."'	animal	META	NP_nom [Agent] V QUOT [Theme]	NP_nom [Agent] V		NEG	agressive, angry
**psikati 'yap'**	Pssst - psikali su ljudi uokolo, ali bakin Kristuš se odbijao od crkvenih zidova i vitraja.<br>'"Pssst," people yapped around, but Granny's Kristuš was bouncing off the church walls and stained glass windows.	animal	META	NP_nom [Agent] V QUOT [Theme]	NP_nom [Agent] V		NEUTRAL	silently
**revati 'bray'**	Jučer ste revali kako je Kerum ružan...<br>'Yesterday, you were braying how Kerum is ugly=about how Kerum is ugly...'	animal	META	NP_nom [Agent] V kako ('that')_CP [Theme]	NP_nom [Agent] V		NEG	loud, repetitive, stupid
**revati 'bray'**	Nitko gluposti ne reve kao on.<br>'No one brays nonsense like he does.'	animal	META	NP_nom [Agent] V NP_acc [Theme]	NP_nom [Agent] V		NEG	loud, repetitive, stupid
**revati 'bray'**	...nabi si socijalizam u guzu i ne revi vise o tome.<br>'...shove socialism up your ass and stop braying about it.'	animal	META	NP_nom [Agent] V PP_o ('about')+loc [Theme]	NP_nom [Agent] V		NEG	loud, repetitive, stupid
**revati 'bray'**	Rado bi se uljudno bacila na pod i revala : "Vrati se, vrati"<br>'She would gladly have politely thrown herself on the floor and brayed, "Come back, come back."'	animal	META	NP_nom [Agent] V QUOT [Theme]	NP_nom [Agent] V		NEG	loud, repetitive, stupid
**režati 'howl'**	Joj kad se sjetim kak ste režali za Šukija, a sad režite na njega.<br>'Oh, I remember how you used to all howl for Šuki, and now you're howling at him.'	animal	META	NP_nom [Agent] V PP_na ('at')+acc [Patient]/[Recipient]	NP_nom [Agent] V	NP_nom [Agent] V PP_na ('at')+acc [Recipient]	NEG	agressive, angry
**režati 'howl'**	Joj kad se sjetim kak ste režali za Šukija, a sad režite na njega.<br>'Oh, I remember how you used to all howl for Šuki, and now you're howling at him.'	animal	META	NP_nom [Agent] V PP_za ('for')+acc [Beneficiary]/[Theme]	NP_nom [Agent] V	NP_nom [Agent] V PP_na ('at')+acc [Recipient]	NEG	agressive, angry
**režati 'howl'**	Imao si samo devet, kad je kidnula sa onim svojim pastuhom - režao bi stari.<br>'You were only nine when she ran off with that stallion of hers, the old man would howl.'	animal	META	NP_nom [Agent] V QUOT [Theme]	NP_nom [Agent] V	NP_nom [Agent] V PP_na ('at')+acc [Recipient]	NEG	agressive, angry
**rikati 'roar'**	Bandić je početkom dvijetisućitih gotovo rikao da će se javno spaliti na Jelačić placu ako za četiri godine trajno ne riješi problem otpada.<br>'At the beginning of the 2000s, Bandić almost roared that he would publicly self-immolate at Jelačić Square if he didn't permanently solve the waste problem in four years.'	animal	META	NP_nom [Agent] V da ('that')_CP [Theme]	NP_nom [Agent] V		NEG	loud, agressive
**rikati 'roar'**	Poštenje mi vrati - rikaše gluhim i promuklim glasom luđakinja...<br>'"Return my honor," she roared in a deafening, hoarse voice of a madwoman.'	animal	META	NP_nom [Agent] V QUOT [Theme]	NP_nom [Agent] V		NEG	loud, agressive
**riknuti 'roar'**	Od užasa, ispala mi je iz ruku knjiga, »Nevina u ludnici«, kad je neki dan na tv šef Sabora riknuo da su prevareni.<br>'In horror, the book "Innocent in the Asylum" slipped from my hands when the other day, on TV, the Speaker of the Parliament roared that they were deceived.'	animal	META	NP_nom [Agent] V da ('that')_CP [Theme]	NP_nom [Agent] V		NEG	loud, agressive
**riknuti 'roar'**	Ona mlada majka dignu mrtvo dijete s krila i riknu na Doru: - Gledaj, gledaj, stara vještice, što si učinila!<br>'The young mother lifted the dead child from her lap and roared at Dora: "Look, look, old witch, what you've done!"'	animal	META	NP_nom [Agent] V QUOT [Theme] PP_na ('at')+acc [Patient]/[Recipient]	NP_nom [Agent] V		NEG	loud, agressive
**roktati 'grunt'**	Još nisi rekla smiješ li ti njemu rovati po mobitelu bez da on "rokće" na tebe.<br>'You still haven't said if you're allowed to rummage through his phone without him "grunting" at you.'	animal	META	NP_nom [Agent] V PP_na ('at')+acc [Patient]/[Recipient]	NP_nom [Agent] V		NEG	repetitive, agressive

VERB	EXAMPLE	SOURCE DOMAIN	METAPHOR/METONYMY	VALENCY FRAME OF THE EXAMPLE	VALENCY FRAME IN THE SOURCE DOMAIN	VALENCY FRAME IN THE SOURCE DOMAIN	EMOTIONAL VALENCY	HIGHLIGHTED MAPPING
**roktati 'grunt'**	Lijevi politički blefери već deset godina rokću o vlasti dr. Franje Tuđmana kao o deset godina mraka i pljačke. 'Left-wing political bluffer have been grunting for ten years about Dr. Franjo Tuđman's rule as a decade of darkness and theft.'	animal	META	NP_nom [Agent] V PP_o ('about')+loc [Theme]	NP_nom [Agent] V		NEG	repetitive, stupid, irritating
**roktati 'grunt'**	...pa je opet počeo roktati o nekim tamo predsjednicima, koji su naravno njemu uništili život. '...and he started grunting again about some presidents who, of course, ruined his life.'	animal	META	NP_nom [Agent] V PP_o ('about')+loc [Theme]	NP_nom [Agent] V		NEG	repetitive, stupid
**roktati 'grunt'**	Pucaj, svinjo - prezirno rokćem , izazivajući ga. '"Shoot, pig," I grunt contemptuously, provoking him.'	animal	META	NP_nom [Agent] V QUOT [Theme]	NP_nom [Agent] V		NEG	repetitive
**rzati 'neigh'**	Živ sam, živ sam - radosno ržem. 'I'm alive, I'm alive – I neigh joyfully.'	animal	META	NP_nom [Agent] V QUOT [Theme]	NP_nom [Agent] V		POSITIVE	strong, happy
**siknuti 'hiss'**	Zbog čega ste siknuli na mene, zbog toga što ne dijelim vaše stavove. 'Why did you hiss=snap at me? Because I don't share your views.'	animal	META	NP_nom [Agent] V PP_na ('at')+acc [Patient]/[Recipient]	NP_nom [Agent] V		NEG	agressive, angry
**siknuti 'hiss'**	- Nemojte me prekidati - sikne ona. '"Don't interrupt me," she hisses.'	animal	META	NP_nom [Agent] V QUOT [Theme]	NP_nom [Agent] V		NEG	agressive, angry
**siktati 'hiss'**	Nevjerojatno mi puno znači da je sa mnom, dovoljno mi je da me drži za ruku, a na pokušaje da me hrabri za vrijeme trudova neromantično sikćem da ušuti. 'It means an incredible amount to me that he's with me, it's enough that he holds my hand, and at his attempts to encourage me during labor, I unromantically hiss at him to shut up.'	animal	META	NP_nom [Agent] V da ('that')_CP [Theme]	NP_nom [Agent] V		NEG	agressive, angry
**siktati 'hiss'**	Ipak, morat će još čekati, jer oficir ne pruža ruku prema ženi, i ne sikće ružne riječi. 'However, he'll have to wait a bit longer, because the officer doesn't extend his hand to a woman, nor does he hiss ugly words.'	animal	META	NP_nom [Agent] V NP_acc [Theme]	NP_nom [Agent] V		NEG	agressive, angry
**siktati 'hiss'**	...koji ni mjesec dana nakon izbora još nije prestao uvrijeđeno siktati na "izdajice". '...who, even a month after the elections, still hasn't stopped hissing angrily at the "traitors."'	animal	META	NP_nom [Agent] V PP_na ('at')+acc [Patient]/[Recipient]	NP_nom [Agent] V		NEG	agressive, angry
**siktati 'hiss'**	Kosor sikće na priču o броševima. 'Kosor hisses at the story about the brooches.'	animal	META	NP_nom [Agent] V PP_na ('at')+acc [Theme]	NP_nom [Agent] V		NEG	agressive, angry
**siktati 'hiss'**	Neka sikću po partizanima koji još imaju mirovine i nitko ih ne proziva. 'Let them hiss at the partisans who still receive pensions and no one calls them out.'	animal	META	NP_nom [Agent] V PP_po ('over')+loc [Patient]/[Recipient]	NP_nom [Agent] V		NEG	agressive, angry
**siktati 'hiss'**	"Utopi mule, udaj se za mene!" – sikće joj. '"Drop the mules, marry me!" – he hisses to=at her.'	animal	META	NP_nom [Agent] V QUOT [Theme] NP_dat [Patient]/[Recipient]	NP_nom [Agent] V		NEG	agressive, angry
**siktati 'hiss'**	- Ne možeš samo tako - siktala je prema meni. '"You can't just do that" – she hissed towards=at me.'	animal	META	NP_nom [Agent] V QUOT [Theme] PP_prema+loc [Goal]	NP_nom [Agent] V		NEG	agressive, angry
**skičati 'squeak'**	Oprostite, ali ja ovo love šta zaradim, zaradim pošteno i ne dam je na tarife, nove uređaje, popuste bez popusta ma koliko neke debele drugorazredne pjevačice skičale o tome. 'Sorry, but I earn this money honestly, and I don't spend it on tariffs, new devices, or discounts without discounts, no matter how much some fat, second-rate singers squea about it.'	animal	META	NP_nom [Agent] V PP_o ('about')+loc [Theme]	NP_nom [Agent] V		NEG	loud, irritating, high pitch
**skičati 'squeak'**	Skičao bi: "Debiliiii" 'He would squeal: "Idiots!"'	animal	META	NP_nom [Agent] V QUOT [Theme]	NP_nom [Agent] V		NEG	loud, irritating, high pitch

VERB	EXAMPLE	SOURCE DOMAIN	METAPHOR/METONYMY	VALENCY FRAME OF THE EXAMPLE	VALENCY FRAME IN THE SOURCE DOMAIN	VALENCY FRAME IN THE SOURCE DOMAIN	EMOTIONAL VALENCY	HIGHLIGHTED MAPPING
**skičati 'squeak'**	Kad se spomenilo 10 % pdv-a odma su poćeli skričat kako neće smanjit cjene.<br>'When the 10% VAT was mentioned, they immediately started squealing=screeching about how they wouldn't lower prices.'	animal	META	NP_nom [Agent] V kako ('that')_CP [Theme]	NP_nom [Agent] V		NEG	loud, irritating, high pitch
**skvičati 'squeak'**	...dok ove što skviče kako imaju celulitne guzice i zbog toga puno pate u životu.<br>'...while those who squeal about having cellulite on their butts and suffer a lot in life because of it.'	animal	META	NP_nom [Agent] V kako ('that')_CP [Theme]	NP_nom [Agent] V		NEG	irritating, high pitch
**skvičati 'squeak'**	Uvijek se skoro onesvijesti od oduševljenja i skviči nešto na portugalskom ili španjolskom.<br>'She almost always faints from excitement and squeals something in Portuguese or Spanish.'	animal	META	NP_nom [Agent] V NP_acc [Theme]	NP_nom [Agent] V		NEG	loud, irritating, high pitch
**skvičati 'squeak'**	Kao branitelj s pokrićem nema ništa protiv javne objave, a zaveden masom onih koji skviče o objavi…<br>'As a veteran with backing, he has nothing against the public announcement, while being swayed by the crowd of those who squeal about the release…'	animal	META	NP_nom [Agent] V PP_o ('about')+loc [Theme]	NP_nom [Agent] V		NEG	loud, irritating, high pitch
**skvičati 'squeak'**	Mali vražić istog trena krene hopsati gore-dolje i skvičati na sav glas: " Jel vidiš ti kak ' to odlično izgleda, kak ' to zvuči, kak ' miriše?<br>'The little devil immediately starts hopping up and down, squealing at the top of his lungs: "Do you see how great that looks, how it sounds, how it smells?"'	animal	META	NP_nom [Agent] V QUOT [Theme]	NP_nom [Agent] V		NEG	loud, irritating, high pitch
**šištati 'hiss'**	Uzmi mi lovu za one knjige - šišti ona s ulaza...<br>'"Take my money for those books," she hisses from the entrance.'	animal	META	NP_nom [Agent] V QUOT [Theme]	NP_nom [Agent] V		NEUTRAL	loud
**štektati 'yap'**	Dakle, sljedeći put prije nego počnete štektati na mene, sjetite se da nitko nije savršen i da svatko ima razdoblje u životu kada mu štošta nije jasno.<br>'So, next time before you start yapping at me, remember that no one is perfect and everyone has a period in their life when many things aren't clear.'	animal	META	NP_nom [Agent] V PP_na ('at')+acc [Patient]/[Recipient]	NP_nom [Agent] V	NP_nom [Agent] V PP_na ('at')+acc [Recipient])	NEG	agressive
**zablejati 'bleat'**	Tu i tamo, dnevnopolitičkim ideološkim povodom, bolje reći podvalom, nekakva budala zableji ponešto o njemu kako bi pokriven njegovim genijem i autoritetom napakostio i osudio drugu misao.<br>'Here and there, on a daily political ideological pretext, or rather a trick, some fool bleats a bit about him in order to cover up his genius and authority while harming and condemning another thought.'	animal	META	NP_nom [Agent] V NP_acc [Theme]	NP_nom [Agent] V		NEG	stupid, irritating
**zablejati 'bleat'**	Crkni homofobna, fašistička tvorevino... " zablejala je mlada SDP-ovca sa Korčule.<br>'"Die, you homophobic, fascist creation..." the young SDP member from Korčula bleated.'	animal	META	NP_nom [Agent] V QUOT [Theme]	NP_nom [Agent] V		NEG	stupid, irritating
**zabrundati 'grunt'**	Probudio se i zabrundao nešto.<br>'He woke up and grunted something.'	animal	META	NP_nom [Agent] V NP_acc [Theme]	NP_nom [Agent] V		NEG	complaining, loud
**zabrundati 'grunt'**	Vi biste, kumo, mogli kod kuće ostati, kod preslice – zabrunda Tomo.<br>'You could, godmother, stay at home, by the spinning wheel – Tomo grunted.'	animal	META	NP_nom [Agent] V QUOT [Theme]	NP_nom [Agent] V		NEG	complaining, loud
**zacičati 'squeak'**	Ono nije rakija, ono je otrov za štakore, zacičao je Božo kad smo se jutros probudili u autu na savskom nasipu.<br>'That's not rakija, that's rat poison, Božo squeaked when we woke up this morning in the car on the Sava embankment.'	animal	META	NP_nom [Agent] V QUOT [Theme]	NP_nom [Agent] V		NEG	high pitch, loud

VERB	EXAMPLE	SOURCE DOMAIN	METAPHOR/METONYMY	VALENCY FRAME OF THE EXAMPLE	VALENCY FRAME IN THE SOURCE DOMAIN	VALENCY FRAME IN THE SOURCE DOMAIN	EMOTIONAL VALENCY	HIGHLIGHTED MAPPING
**zacviliti 'whine'**	Naravno da život teče mimo njih, a ti „baš me briga majstori" povremeno zacvile kako nemaju ovo ili ono. 'Of course, life goes on without them, and you, "I couldn't care less, experts," occasionally whine how they don't have this or that=about how they don't have this or that.'	animal	META	NP_nom [Agent] V kako ('that')_CP [Theme]	NP_nom [Agent] V		NEG	complaining, high pitch, irritating
**zacviliti 'whine'**	...stao usred sobe i zacvilio o ljubavi i o strasti. 'He stopped in the middle of the room and whined about love and passion.'	animal	META	NP_nom [Agent] V PP_o ('about')+loc [Theme]	NP_nom [Agent] V		NEG	high pitch, irritating
**zacviliti 'whine'**	U pomoć, u pomoć!- zacvili djevojka. 'Help, help! – the girl whined.'	animal	META	NP_nom [Agent] V QUOT [Theme]	NP_nom [Agent] V		NEG	high pitch, loud, fear
**zacvrkutati 'chirp, cricket'**	Stari je zacvrkutao nešto u smjeru kuhinje. 'The old man chirped something in the direction of the kitchen.'	animal	META	NP_nom [Agent] V NP_acc [Theme]	NP_nom [Agent] V		POSITIVE	cheerful, pleasant
**zacvrkutati 'chirp, cricket'**	Bez Jožice ne može biti nijedne sjajnije i bolje svadbe - zacvrkuta Jaga. 'Without Jožica, there can't be any shinier or better wedding, chirped Jaga.'	animal	META	NP_nom [Agent] V QUOT [Theme]	NP_nom [Agent] V		POSITIVE	cheerful, pleasant
**zacvrkutati 'chirp, cricket'**	"Danas imam date" zacvrkutala mi je na mom odlasku. '"Today I have a date," she chirped to me as I was leaving.'	animal	META	NP_nom [Agent] V QUOT [Theme] NP_dat [Recipient]	NP_nom [Agent] V		POSITIVE	cheerful, pleasant
**zakokodakati 'cluck'**	Joj sada samo čekam da mi ona moja nešto kaže kada dođem kod nje, samo neka zakokodače kako sam joj zapišao wc i sve oko njega. 'Oh, now I'm just waiting for her to say something when I get to her place, just let her cluck how I pissed all over the toilet and everything around it=about how I pissed all over the toilet and everything around it.'	animal	META	NP_nom [Agent] V kako ('that')_CP [Theme]	NP_nom [Agent] V		NEG	loud, irritating, exaggerating importance
**zakokodakati 'cluck'**	...jer kad na plaži zakokodaču u komunističkoj manirima za nekakvim tobože pravom za mjesto na plaži... '...because when they cluck on the beach in communist fashion after=about some so-called right to a spot on the beach.'	animal	META	NP_nom [Agent] V PP_za ('for')+instr [Theme]	NP_nom [Agent] V		NEG	loud, irritating, collective
**zakokodakati 'cluck'**	"Oh, gle", zakokodače Hazel. '"Oh, look," Hazel clucks.'	animal	META	NP_nom [Agent] V QUOT [Theme]	NP_nom [Agent] V		NEG	loud, exaggerating importance
**zakričati 'screech'**	A ja mu, 2 godišnjakinja, zakričah: Marš čiko, piccka ti materina. 'And I, a 2-year-old, screeched=screamed at him: "Go away, you bastard!"'	animal	META	NP_nom [Agent] V QUOT [Theme] NP_dat [Patient]/[Recipient]	NP_nom [Agent] V		NEG	loud, irritating, agressive
**zakričati 'screech'**	Što si se sada ovdje ustobočio? - zakriča zlovoljno na me Mecena. '"What are you standing there for now?" Mecena screeched grumpily at me.'	animal	META	NP_nom [Agent] V QUOT [Theme] PP_na ('at')+acc [Patient]/[Recipient]	NP_nom [Agent] V		NEG	loud, irritating, agressive
**zakriještati 'screech'**	Kako je stari samo zakriještao da moramo doći na cijepljenje. 'How the old man just screeched that we have to go for the vaccination.'	animal	META	NP_nom [Agent] V da ('that')_CP [Theme]	NP_nom [Agent] V		NEG	loud, irritating
**zakriještati 'screech'**	MMF-ovac, neki Grk, odmah je histerično zakriještao protiv toga. 'The IMF guy, some Greek, immediately screeched hysterically against it.'	animal	META	NP_nom [Agent] V PP_protiv ('against')+gen [Theme]	NP_nom [Agent] V		NEG	loud, irritating
**zakriještati 'screech'**	"Čujmo, čujmo", zakriješti Ivan Zaboki. '"Listen, listen," Ivan Zaboki screeched.'	animal	META	NP_nom [Agent] V QUOT [Theme]	NP_nom [Agent] V		NEG	loud, irritating
**zakukurikati 'crow'**	Naravno, netko će zakukurikati : A gdje su dokazi? 'Of course, someone will crow: "But where's the evidence?"'	animal	META	NP_nom [Agent] V QUOT [Theme]	NP_nom [Agent] V		NEG	loud, sudden, irritating

VERB	EXAMPLE	SOURCE DOMAIN	METAPHOR/METONYMY	VALENCY FRAME OF THE EXAMPLE	VALENCY FRAME IN THE SOURCE DOMAIN	VALENCY FRAME IN THE SOURCE DOMAIN	EMOTIONAL VALENCY	HIGHLIGHTED MAPPING
**zalajati 'bark'**	Lovrek Štef se jasno sjećao toga kako ga je Trdak pritegao za remen i kako je posve mekan stao da bljuje krv i kako je Pesek zalajao na njega s revolverom u ruci. 'Lovrek Štef clearly remembered how Trdak grabbed him by the belt and how he suddenly collapsed, vomiting blood, and how Pesek barked at him with a revolver in his hand.'	animal	META	NP_nom [Agent] V PP_na ('at')+acc [Patient]/[Recipient]	NP_nom [Agent] V	NP_nom [Agent] V PP_na ('at')+acc [Recipient])	NEG	agressive, harming (psych.)
**zalajati 'bark'**	Što čekate? - zalaje general. '"What are you waiting for?" the general barked.'	animal	META	NP_nom [Agent] V QUOT [Theme]	NP_nom [Agent] V	NP_nom [Agent] V PP_na ('at')+acc [Recipient])	NEG	agressive, harming (psych.)
**zameketati 'bleat'**	Sladoled, friški, ledeni! – zamekeće katkada ovogodišnji sladoledžija Selim svojim tankim zanosećim, "orijentalnim" glasom. '"Ice cream, fresh, icy!" – the ice cream vendor Selim will occasionally bleat in his thin, enchanting, "oriental" voice.'	animal	META	NP_nom [Agent] V QUOT [Theme]	NP_nom [Agent] V		NEG	repetitive, irritating
**zamijukati 'meow'**	No gdje si sad, zamijaukala je nestrpljivo iz kuhinje. 'Well, where are you now? she meowed impatiently from the kitchen.'	animal	META	NP_nom [Agent] V QUOT [Theme]	NP_nom [Agent] V		NEUTRAL	loud
**zamukati 'moo'**	Naravno, revoltirani biciklisti će odmah u zboru složno zamukati : "Pa kako da se vozimo ulicama u kojima nema označenih... 'Of course, the disgruntled cyclists will immediately moo in unison: "How are we supposed to ride on streets that have no designated..."'	animal	META	NP_nom [Agent] V QUOT [Theme]	NP_nom [Agent] V		NEG	irritating, collective, in vain
**zanjakati 'heehaw'**	Nisam zaspao - zanjače bolesnik - ne znam dalje brojati. 'I haven't fallen asleep, heehaws=whines the sick man – I can't count any further.'	animal	META	NP_nom [Agent] V QUOT [Theme]	NP_nom [Agent] V		NEG	strange, stupid?
**zarežati 'growl'**	Što sam ja? - zarežao sam na nju prekidajući je. 'What am I? – I growled at her, interrupting her.'	animal	META	NP_nom [Agent] V QUOT [Theme] PP_na ('at')+acc [Patient]/[Recipient]	NP_nom [Agent] V	NP_nom [Agent] V PP_na ('at')+acc [Recipient])	NEG	agressive, loud
**zaroktati 'grunt'**	Ovako ćemo, Prdiću... " - zarokće ona. '"Here's how we'll do it, Prdiću..." – she grunted.'	animal	META	NP_nom [Agent] V QUOT [Theme]	NP_nom [Agent] V		NEG	irritating, repulsive
**zaštektati 'yap'**	Da, prije nego zaštekćete kako se to rješava običnim pranjem dotičnih... 'Yes, before you start yapping how this is solved=about how this is solved by simply washing the respective ones...'	animal	META	NP_nom [Agent] V kako ('that')_CP [Theme]	NP_nom [Agent] V		NEG	unpleasant, loud
**zaštektati 'yap'**	Dobna skupina od trideset do četrdeset! – zaštektao je prodorni ženski glas. 'Age group from thirty to forty! – yapped a piercing female voice.'	animal	META	NP_nom [Agent_METO] V QUOT [Theme]	NP_nom [Agent] V		NEG	unpleasant, loud
**zavijati 'howl'**	A vi se okupljajte i zavijajte svi uglas kako vas vrijedja cirilica... 'And you all gather and howl together how offenden you are by Cyrillic=about how offended you are by Cyrillic...'	animal	META	NP_nom [Agent] V kako ('that')_CP [Theme]	NP_nom [Agent] V		NEG	loud, helpless
**zavijati 'howl'**	...pošto će UVJEK upotrijebiti civilizacijske dosege zapadne civilizacije KAD TO NJIMA PAŠE, a ostatak vremena će zavijat o 1232. godini i dekadentnom zapadu. '...since they will ALWAYS use the civilizational achievements of Western civilization WHEN IT SUITS THEM, and the rest of the time, they will howl about the year 1232 and the decadent West.'	animal	META	NP_nom [Agent] V PP_o ('about')+loc [Theme]	NP_nom [Agent] V		NEG	repetitive, irritating, boring

VERB	EXAMPLE	SOURCE DOMAIN	METAPHOR/METONYMY	VALENCY FRAME OF THE EXAMPLE	VALENCY FRAME IN THE SOURCE DOMAIN	VALENCY FRAME IN THE SOURCE DOMAIN	EMOTIONAL VALENCY	HIGHLIGHTED MAPPING
**zavijati 'howl'**	I dgje je onaj strip u kojem ženska zavija protiv svega a kada joj se kaže da neka ona u to vjeruje a nas neka pusti na miru ona počne zavijati kako je proganjaju i osuđuju.<br>'And where is that comic where the woman howls against everything, and when she's told to believe in it herself and leave us alone, she starts howling that she's being persecuted and condemned?'	animal	META	NP_nom [Agent] V PP_protiv ('against')+gen [Theme]	NP_nom [Agent] V		NEG	repetitive, irritating, boring
**živkati 'chirp'**	I živkaj ti o ustašama koliko hoćeš…<br>'And you can chirp about the Ustashe as much as you want...'	animal	META	NP_nom [Agent] V PP_o ('about')+loc [Theme]	NP_nom [Agent] V		NEG	irritating
**žvrgoljiti 'chirp'**	Poslije završila na operaciji, ali ništa nema cijene kada ga sad, nakon 15 mjeseci gledam kako hoda, igra se, žvrgolji neku svoju priču.<br>'Afterwards, she ended up in surgery, but nothing is more valuable than seeing him now, after 15 months, walking, playing, and chirping some of his own stories.'	animal	META	NP_nom [Agent] V NP_acc [Theme]	NP_nom [Agent] V		POSITIVE	pleasant, child speech, hard to understand
**žvrgoljiti 'chirp'**	...mi smo žvrgoljili o koječemu vozeći se starom cestom prema moru.<br>'...we chirped about all sorts of things while driving down the old road to the sea.'	animal	META	NP_nom [Agent] V PP_o ('about')+loc [Theme]	NP_nom [Agent] V		NEUTRAL	superficial
**cvokotati 'chatter, shiver'**	...nećemo u tramvaju cvokotati kako nam ova današnja mladež ne želi ustupiti mjesto.<br>'...we're not going to chatter in the tram how today's youth refuses to give up their seats=about how today's youth refuses to give up their seats.'	bodily processes	META	NP_nom [Agent] V kako ('that')_CP [Theme]	NP_nom [Theme] V NP_inst [Instrument]	NP_nom [Theme] V	NEG	
**cvokotati 'chatter, shiver'**	"Vidjela sam Martina", cvokotala je pored nje.<br>'"I saw Martin," she chattered beside her.'	bodily processes	META	NP_nom [Agent] V QUOT [Theme]	NP_nom [Theme] V NP_inst [Instrument]	NP_nom [Theme] V	NEG	
**drkati 'jerk off'**	...ja nju drkam da shta pije cedevitu balance.<br>'I jerk her off why she drinks cedevita balance=I am criticizing her for drinking Cedevita Balance.'	bodily processes	META	NP_nom [Agent] V NP_acc [Patient] da ('that')_CP [Theme]	NP_nom [Agent] V		NEG	
**drkati 'jerk off'**	Netko tko drka o demokraciji bez da se osvrne na informacije koje sam ponudio u gornjem linku.<br>'Someone who jerks off about democracy without considering the information I provided in the link above.'	bodily processes	META	NP_nom [Agent] V PP_o ('about')+loc [Theme]	NP_nom [Agent] V		NEG	
**kakiti 'poop'**	Naravno, bilo bi i onih koji bi došli na most Voyagera i kakili kako je sve kvadratično i nepraktično.<br>'Of course, there would be those who would come to the Voyager bridge and poop about how everything is square and impractical.'	bodily processes	META	NP_nom [Agent] V kako ('that')_CP [Theme]	NP_nom [Agent] V		NEG	
**kakiti 'poop'**	Ti samo kakiš o moralnosti i osuđuješ male ljude.<br>'You just poop about morality and judge the little people.'	bodily processes	META	NP_nom [Agent] V PP_o ('about')+loc [Theme]	NP_nom [Agent] V		NEG	
**kakiti 'poop'**	...kada ćeš početi malo kakiti po komunjarama?<br>'when will you start pooping/shitting on the communists?'	bodily processes	META	NP_nom [Agent] V PP_po ('over')+loc [Theme]	NP_nom [Agent] V		NEG	
**kakiti 'poop'**	Pristani više kakit protiv Keruma svaki drugi dan.<br>'Stop shitting=talking shit against Kerum every other day.'	bodily processes	META	NP_nom [Agent] V PP_protiv ('against')+gen [Theme]	NP_nom [Agent] V		NEG	
**kenjati 'crap'**	Joj, kak mrzim kad tobožnji radoholičari iz ljubomore kenjaju kako smo mi balkanci.<br>'Ugh, how I hate it when so-called workaholics crap=whine out of jealousy that we are Balkans=about how we Balkans are.'	bodily processes	META	NP_nom [Agent] V kako ('that')_CP [Theme]	NP_nom [Agent] V		NEG	
**kenjati 'crap'**	...onda kenjaju curama na netu kako oni nemaju nikakve pretenzije osim "razgovora".<br>'..then they crap=lie to the girls online that they have no intentions other than "talking."'	bodily processes	META	NP_nom [Agent] V kako ('that')_CP [Theme] NP_dat [Recipient]	NP_nom [Agent] V		NEG	

VERB	EXAMPLE	SOURCE DOMAIN	METAPHOR/METONYMY	VALENCY FRAME OF THE EXAMPLE	VALENCY FRAME IN THE SOURCE DOMAIN	VALENCY FRAME IN THE SOURCE DOMAIN	EMOTIONAL VALENCY	HIGHLIGHTED MAPPING
**kenjati 'crap'**	...ili da ja kenjam nekome građevinaru, a razmem se u to ko krava u balet.<br>'...or should I crap=nag to some construction worker, when I don't know anything about it, like a cow in ballet?'	bodily processes	META	NP_nom [Agent] V NP_dat [Recipient]	NP_nom [Agent] V		NEG	
**kenjati 'crap'**	Ne znam zasto ljudi toliko kenjaju o hrvatskoj obali.<br>'I don't know why people crap=talk shit so much about the Croatian coast.'	bodily processes	META	NP_nom [Agent] V PP_o ('about')+loc [Theme]	NP_nom [Agent] V		NEG	
**kenjati 'crap'**	To je kao da kenjaš po filmu , a nisi ga pogledao.<br>'It's like crapping on the movie=whining about a movie when you haven't even watched it.'	bodily processes	META	NP_nom [Agent] V PP_po ('about')+loc [Theme]	NP_nom [Agent] V		NEG	
**nasrati**	Muskarcina je vjerojatno nasrao da sam ga ja NAGOVORIO da ide a ona je povjerovala.<br>'The guy probably shit-talked her into believing that I CONVINCED him to go. '	bodily processes	META	NP_nom [Agent] V da ('that')_CP [Theme]	NP_nom [Agent] V		NEG	
**nasrati**	Eto, malo sam nasrao gluposti al mi smo presretni sa ovim rezultatom.<br>'Well, I've talked some crap, but we're totally thrilled with these results.'	bodily processes	META	NP_nom [Agent] V NP_acc [Theme]	NP_nom [Agent] V		NEG	
**nasrati**	Što ti je nasrao svakakve gluposti o meni?<br>'What bullshit has he been spouting about me?'	bodily processes	META	NP_nom [Agent] V NP_acc [Theme] NP_dat [Recipien]	NP_nom [Agent] V		NEG	
**nasrati se**	Vidim da ste se nasrali o Boriću.<br>'I see that you've been shit-talking about Borić.'	bodily processes	META	NP_nom [Agent] V PP_o ('about')+loc [Theme]	NP_nom [Agent] V		NEG	
**pljuvati 'spit'**	Odjednom je vaš, a do prije dva tjedan ste pljuvali kako malograđani glasaju za njega.<br>'Suddenly he's yours, yet just two weeks ago you were spitting=slamming how the petty bourgeois vote for him.'	bodily processes	META	NP_nom [Agent] V kako ('that')_CP [Theme]	NP_nom [Agent] V		NEG	
**pljuvati 'spit'**	...koliko god kod kuće pljuvali domovinu , vani je počinjemo strasno voljeti.<br>'...no matter how much we spit on the homeland=badmouth the homeland at home, abroad we begin to love it passionately.'	bodily processes	META	NP_nom [Agent] V NP_acc [Theme]	NP_nom [Agent] V		NEG	
**pljuvati 'spit'**	I zašto ljudi stalno tako pljuju o njoj, a stvarno na temelju ničega.<br>'And why do people keep spitting about her=badmouthing her like that, really without any basis?'	bodily processes	META	NP_nom [Agent] V PP_o ('about')+loc [Theme]	NP_nom [Agent] V		NEG	
**pljuvati 'spit'**	Danas je moderno pljuvati po J. Kosor.<br>'Nowdays, it's trendy to spit on J. Kosor=to badmouth J. Kosor.'	bodily processes	META	NP_nom [Agent] V PP_po ('about')+loc [Theme]	NP_nom [Agent] V		NEG	
**pljuvati 'spit'**	I kako ja to pljujem protiv Hrvata ako tvrdim da su srpski i hrvatski dva jezika?<br>'And how am I spitting against Croats=slamming Croats if I claim that Serbian and Croatian are two languages?'	bodily processes	META	NP_nom [Agent] V PP_protiv ('against')+gen [Theme]	NP_nom [Agent] V		NEG	
**popišati 'piss'**	Meni se to događa ponekad, osjećam se super sa svojom šakom zadovoljstva i sreće i smijem se kao da sam najsretnija na svijetu jer me kukanje ne veseli premda i kukam jer sam eto samo čovjek. Onda dođe netko i popiša me samo da se osjeća bolje, nadmoćnije i sretnije.<br>'This happens to me sometimes, I feel great with my little fist of satisfaction and happiness, and I laugh as if I'm the happiest person in the world because complaining doesn't make me happy, even though I complain because, well, I'm just human. Then someone comes along and pisses me=on me just to feel better, more superior, and happier.'	bodily processes	META	NP_nom [Agent] V NP_acc [Patient]/[Patient/Recipient]	NP_nom [Agent] V NP_acc [Patient]		NEG	
**posrati 'shit'**	Najlakše je posrat svoga, to je nama naša borba dala.<br>'The easiest thing is to shit your own=on your own, that's what our struggle gave us.'	bodily processes	META	NP_nom [Agent] V NP_acc [Patient]/[Theme]	NP_nom [Agent] V (NP_acc[Theme])		NEG	

VERB	EXAMPLE	SOURCE DOMAIN	METAPHOR/METONYMY	VALENCY FRAME OF THE EXAMPLE	VALENCY FRAME IN THE SOURCE DOMAIN	VALENCY FRAME IN THE SOURCE DOMAIN	EMOTIONAL VALENCY	HIGHLIGHTED MAPPING
**posrati 'shit'**	...dala sebi za pravo da me "posere" u komentaru. '...took it upon herself to "shit" me=on me in the comment.'	bodily processes	META	NP_nom [Agent] V NP_acc [Theme/Patient]/[Theme]	NP_nom [Agent]		NEG	
**posrati 'shit'**	Upravo ti ljudi koji su to sve organizirali i koji su se odlucili oprostite na izrazu posrat na Sandru Perković. 'It's precisely those people who organized everything and decided, excuse my language, to shit on Sandra Perković.'	bodily processes	META	NP_nom [Agent] V PP_na ('at')+acc [Theme]	NP_nom [Agent] V (NP_acc[Theme])		NEG	
**prdnuti 'fart'**	...jednako kao što amerika prdne da donosi "demokraciju" i domah svi bleje da je nastupila - demokracija a to što je očito da od demokracije nema ni D. '...just like America farts that it brings "democracy," and immediately everyone bleats that democracy has arrived, even though it's obvious there's not even a D of democracy.'	bodily processes	META	NP_nom [Agent] V da ('that')_CP [Theme]	NP_nom [Agent]		NEG	
**prdnuti 'fart'**	Ajd prdnem i ja nešto na slovenskom. 'Alright, I fart something in Slovenian too.'	bodily processes	META	NP_nom [Agent] V NP_acc [Theme]	NP_nom [Agent]		NEG	
**prdnuti 'fart'**	Kao i svaki put kad pokušavam saznati di je i kaj je i jel mu glava na ramenu, uredno mi u uho prdne ono iritantno " You have reached the O2 mailbox..	bodily processes	META	NP_nom [Agent] V QUOT [Theme] NP_dat [Recipient]	NP_nom [Agent]		NEG	
**prosrati 'shit out'**	Sad, nek mi netko samo prosere da se to radi po "Pruskoj" ili ne znam kakvoj školi… 'Now, let someone just shit to=on me (by saying) that this is done according to "Prussian" or some other school of thought…'	bodily processes	META	NP_nom [Agent] V da ('that')_CP [Theme] NP_dat [Recipient]			NEG	
**prosrati 'shit out'**	A koliko se ja sjecam, cini mi se da je Wall po obicaju prosrao neku glupost sa teatralnim predznakom. 'As far as I remember, it seems to me that Wall, as usual, shit out some nonsense with a theatrical touch.'	bodily processes	META	NP_nom [Agent] V NP_acc [Theme]			NEG	
**prosrati 'shit out'**	...htjela bi izjadati dusu prosrala si pricu o trudnoci. '...she wanted to vent her soul, and you shit out a story about pregnancy.'	bodily processes	META	NP_nom [Agent] V NP_acc [Theme]			NEG	
**prosrati 'shit out'**	...stric se izvlači iz sobe i još nešto prosere bratiču o stilu života i moralu. '...the uncle drags himself out of the room and then shits out something to my cousin about lifestyle and morality.'	bodily processes	META	NP_nom [Agent] V PP_o ('about')+loc [Theme] NP_dat [Recipient]			NEG	
**prosrati 'shit out'**	Mislio sam nešto nešto naširoko prosrati o Mr. Todoriću i njegovom zlom imperiju koji uništava hrvatska poduzeća al neću dužiti. 'I was thinking of shitting out something about Mr. Todorić and his evil empire that's destroying Croatian companies, but I won't drag it out.'	bodily processes	META	NP_nom [Agent] V PP_o ('about')+loc [Theme]			NEG	
**srati 'shit'**	Sereš ljudima da su nepotrebni i neproduktivni, bome mi i ti djeluješ kao beskorisna jedinka. 'You're shitting to=on people, telling them they're unnecessary and unproductive; well, you sure seem like a useless individual to me too.'	bodily processes	META	NP_nom [Agent] V da ('that')_CP [Theme] NP_dat [Recipient]	NP_nom [Agent]		NEG	
**srati 'shit'**	A meni je ok što si ti nezreo, pa sereš gluposti. 'And I'm fine with you being immature, so you're shitting out nonsense.'	bodily processes	META	NP_nom [Agent] V NP_acc [Theme]	NP_nom [Agent]		NEG	
**srati 'shit'**	Samo neka prestane srat o pravednosti i EMPATIJI. 'Just let them stop shitting=going on about righteousness and EMPATHY.'	bodily processes	META	NP_nom [Agent] V PP_o ('about')+loc [Theme]	NP_nom [Agent]		NEG	

VERB	EXAMPLE	SOURCE DOMAIN	METAPHOR/METONYMY	VALENCY FRAME OF THE EXAMPLE	VALENCY FRAME IN THE SOURCE DOMAIN	VALENCY FRAME IN THE SOURCE DOMAIN	EMOTIONAL VALENCY	HIGHLIGHTED MAPPING
**srati 'shit'**	Sram te bilo tako srati po ljudima, a nisi ni istražila što je istina u cijeloj priči.<br>'Shame on you for shitting on people like that, without even researching what the truth is in the whole story.'	bodily processes	META	NP_nom [Agent] V PP_po ('over')+loc [Theme]	NP_nom [Agent]		NEG	
**srati 'shit'**	Sada je ponovo na novinarskoj konferenciji srao protiv Bobana u stilu " Šta on sere, sredit ću ja njega kada ga vidim...<br>'Now he's back at the press conference, shitting against=on Boban in the style of "What's he talking shit about, I'll take care of him when I see him."'	bodily processes	META	NP_nom [Agent] V PP_protiv ('against')+loc [Theme]	NP_nom [Agent]		NEG	
**srati 'shit'**	Onda sam bio sa starim na kavi, pa mi je srao za ocjene.<br>'Then I was having coffee with my dad, and he was shitting=nagging me about my grades.'	bodily processes	META	NP_nom [Agent] V PP_za ('for')+acc [Theme] NP_dat [Recipient]				
**srati 'shit'**	Srali su mnogi i za Cesarca i za Jugovića pa su ispali odlična pojačanja.<br>'Many people were shitting for=talked shit about Česarac and Jugović, but they turned out to be excellent additions.'	bodily processes	META	NP_nom [Agent] V PP_za ('for')+acc [Theme]				
**dahnuti 'gasp'**	Ne zezaj - dahne Vedrana.<br>'"Don't mess with me," Vedrana gasps.'	breathing	META	NP_nom [Agent] V QUOT [Theme]	NP_nom [Agent] V		NEUTRAL	
**dahnuti 'gasp'**	Jurka - dahnu ona baki - u prvome mjestu nabavi mojoj jagodi što god ljepšeg nađete.<br>'Jurka - she breathed=whispered to grandmother - first of all, get my darling the prettiest thing you find.'	breathing	META	NP_nom [Agent] V QUOT [Theme] NP_dat [Recipient]	NP_nom [Agent] V		NEUTRAL	
**dahtati 'gasp'**	"Previše pljuga premalo konde" dahtao je.<br>'"Too many smokes, too little condition," he gasped.'	breathing	META	NP_nom [Agent] V QUOT [Theme]	NP_nom [Agent] V		NEUTRAL	
**dahtati 'gasp'**	Nećeš - strastveno mu je dahtala u vrat.<br>'You won't – she gasped passionately in his ear.'	breathing	META	NP_nom [Agent] V QUOT [Theme] NP_dat [Recipient]	NP_nom [Agent] V		NEUTRAL	
**soptati 'chug'**	Jebote ' ća me bacilo - soptao je nakon Buka naš skiper crven u licu od neugode.<br>'Fuck, what happened to me? – our skipper Buka chuged=mumbled, his face red with embarrassment.	breathing	META	NP_nom [Agent] V QUOT [Theme]	NP_nom [Agent] V		NEUTRAL	
**soptati 'chug'**	Jedva čekam - i dalje je soptao.<br>'"I can't wait," he kept chugging=gasping.'	breathing	META	NP_nom [Agent] V QUOT [Theme]	NP_nom [Agent] V		NEUTRAL	
**stenjati 'moan'**	Nitko od moje generacije ne stenje da mu je loše.<br>'Nobody from my generation moans that they are doing poorly = moans about not doing well.'	breathing	META	NP_nom [Agent] V da ('that')_CP [Theme]	NP_nom [Agent] V		NEG	
**uzdahnuti 'sigh'**	Uzdahnuo sam: "Da, jasno mi je."<br>'I sighed, "Yes, I understand."'	breathing	META	NP_nom [Agent] V QUOT [Theme]	NP_nom [Agent] V		NEUTRAL	
**crniti 'blacken'**	Nažalost, nisam dobio dojam da me nisu crnili na najgori mogući način i to na neki način zamjeram svom protukandidatu.<br>'Unfortunately, I didn't get the impression that they weren't trying to blacken me=my name in the worst possible way, and in a way, I hold that against my opponent.'	coloring	META	NP_nom [Agent] V NP_acc [Patient]/[Theme]	NP_nom [Agent] V NP_acc [Patient]		NEG	
**ocrniti 'blacken'**	Ovaj komentar pišem u najboljoj namjeri, ne želeći ocrniti tu pjevačicu.<br>'I'm writing this comment with the best intentions, not wanting to blacken that singer=tarnish the reputation of that singer.'	coloring	META	NP_nom [Agent] V NP_acc [Patient]/[Theme]	NP_nom [Agent] V NP_acc [Patient]		NEG	
**jaukati 'moan'**	...odmah požurite u banke i poslije počnite jaukati da nema posla...<br>'...rush to the banks right away, and then start moaning=whining that there's no work...'	crying	META	NP_nom [Agent] V da ('that')_CP [Theme]	NP_nom [Experiencer] V	NP_nom [Experiencer] V PP_nad+inst [Cause]	NEG	
**jaukati 'moan'**	Uporno jaučemo kako nas ništa ne ide.<br>'We persistently moan how notjhing is going our way=whine about how nothing is going our way.'	crying	META	NP_nom [Agent] V kako ('that')_CP [Theme]	NP_nom [Experiencer] V	NP_nom [Experiencer] V PP_nad+inst [Cause]	NEG	

VERB	EXAMPLE	SOURCE DOMAIN	METAPHOR/METONYMY	VALENCY FRAME OF THE EXAMPLE	VALENCY FRAME IN THE SOURCE DOMAIN	VALENCY FRAME IN THE SOURCE DOMAIN	EMOTIONAL VALENCY	HIGHLIGHTED MAPPING
**jaukati 'moan'**	...u ta vremena lijepa naša nije jaukala nad natalitetom. '‘… in those times, our beautiful country did not lament over its birthrate.'	crying	META	NP_nom [Agent] V PP_nad ('over')+inst [Theme]	NP_nom [Experiencer] V	NP_nom [Experiencer] V PP_nad+inst [Cause]	NEG	
**jaukati 'moan'**	Što bi tek bilo da nije tako ugrožen kao što je o tome jaukao po Evropi. 'What would have happened if he weren't as endangered as he whined about across Europe?'	crying	META	NP_nom [Agent] V PP_o ('about')+loc [Theme]	NP_nom [Experiencer] V	NP_nom [Experiencer] V PP_nad+inst [Cause]	NEG	
**jaukati 'moan'**	Volim jaukati o ekonomskoj krizi i otpuhivati dimove bijelog Marlbora. 'I love whining about the economic crisis and blowing smoke from white Marlboros.'	crying	META	NP_nom [Agent] V PP_o ('about')+loc [Theme]	NP_nom [Experiencer] V	NP_nom [Experiencer] V PP_nad+inst [Cause]	NEG	
**jaukati 'moan'**	Metež je trajao, ranjeni putnici zvali u pomoć i jaukali za svojim gubicima. 'The chaos lasted, injured passengers called for help and moaned over=about their losses.'	crying	META	NP_nom [Agent] V PP_za ('for')+inst [Theme]	NP_nom [Experiencer] V	NP_nom [Experiencer] V PP_nad+inst [Cause]	NEUTRAL	
**jaukati 'moan'**	Zašto, zašto? - jaukala je pokrivajući uši dlanovima. 'Why, why?, she wailed, covering her ears with her palms.'	crying	META	NP_nom [Agent] V QUOT [Theme]	NP_nom [Experiencer] V	NP_nom [Experiencer] V PP_nad+inst [Cause]	NEUTRAL	
**jauknuti 'moan'**	Eto, postadoh zec bez prijatelja - jauknu progonjeni i naglo odskoči. 'There you go, I became a rabbit without friends – he moaned and suddenly jumped away.'	crying	META	NP_nom [Agent] V QUOT [Theme]	NP_nom [Experiencer] V	NP_nom [Experiencer] V PP_nad+inst [Cause]	NEUTRAL	
**jecati 'sob'**	Samo je jecala kako njezina sina više nema. 'She just sobbed that her son is gone=She was sobbing about her son being gone.'	crying	META	NP_nom [Agent] V kako ('that')_CP [Theme]	NP_nom [Experiencer] V	NP_nom [Experiencer] V PP_nad+inst [Cause]	NEUTRAL	
**jecati 'sob'**	Manaislović je na mjesto nesreće stigao odmah nakon sudara jecajući nad beživotnim tijelom pregažene životinje. 'Manaislović arrived at the scene of the accident immediately after the collision, moaning over the lifeless body of the run-over animal.'	crying	META	NP_nom [Agent] V PP_nad+inst [Theme]	NP_nom [Experiencer] V	NP_nom [Experiencer] V PP_nad+inst [Cause]	NEUTRAL	
**jecati 'sob'**	Naročito je gnjusno kada se potom u tim istim medijima tobože suosjećajno jeca nad jadom prevarene žene i njezine djece nakon što je i njihova privatnost brutalno provaljena i bezobzirno izložena javnosti. 'It is particularly disgusting when, in those same media, they supposedly sympathetically moan over the misery of the deceived woman and her children after their privacy was brutally invaded and recklessly exposed to the public.'	crying	META	NP_nom [Agent] V PP_nad ('over')+inst [Theme]	NP_nom [Experiencer] V	NP_nom [Experiencer] V PP_nad+inst [Cause]	NEG	
**jecati 'sob'**	Priča nema happy end i završava se uzdahom glavnog junaka dok kroz suze jeca o svojoj sudbini. 'The story has no happy ending and it concludes with the protagonist's sigh as he is sobbing through tears about his fate.'	crying	META	NP_nom [Agent] V PP_o ('about')+loc [Theme]	NP_nom [Experiencer] V	NP_nom [Experiencer] V PP_nad+inst [Cause]	NEUTRAL	
**jecati 'sob'**	"Oprosti mi molim te Saro ", jecala je Nina. '"Forgive me, please, Sara," Nina sobbed.'	crying	META	NP_nom [Agent] V QUOT [Theme]	NP_nom [Experiencer] V	NP_nom [Experiencer] V PP_nad+inst [Cause]	NEUTRAL	
**ječati 'groan'**	Nemoj... nemoj... - ječi ona pod njegovim udarcima. '"Don't... don't..." she groans under his blows.'	crying	META	NP_nom [Agent] V QUOT [Theme]	NP_nom [Experiencer] V	NP_nom [Experiencer] V PP_nad+inst [Cause]	NEUTRAL	
**kmečati 'whine'**	...stalno smo kmečali da hoćemo veliki studio. '...we kept whining that we wanted a big studio.'	crying	META	NP_nom [Agent] V da ('that')_CP [Theme]	NP_nom [Experiencer] V		NEG	
**kmečati 'whine'**	...šta si onako kmečao o islamskim vandalima koji su uništili Konstantinopol? '...why were you whining like that about the Islamic vandals who destroyed Constantinople?'	crying	META	NP_nom [Agent] V PP_o ('about')+loc [Theme]	NP_nom [Experiencer] V		NEG	
**kmečati 'whine'**	Ipak je malo preniska.. - kmečala sam. 'It's still a bit too low... – I whined.'	crying	META	NP_nom [Agent] V QUOT [Theme]	NP_nom [Experiencer] V		NEG	

VERB	EXAMPLE	SOURCE DOMAIN	METAPHOR/METONYMY	VALENCY FRAME OF THE EXAMPLE	VALENCY FRAME IN THE SOURCE DOMAIN	VALENCY FRAME IN THE SOURCE DOMAIN	EMOTIONAL VALENCY	HIGHLIGHTED MAPPING
**kriknuti 'scream, shout out'**	Pošto se vratio, Norac je iz sveg glasa kriknuo : "Šta čekate?" 'After he returned, Norac shouted at the top of his lungs: "What are you waiting for?"'	crying	META	NP_nom [Agent] V QUOT [Theme]			NEUTRAL	
**kriknuti 'scream, shout out'**	"Stanite" kriknula je vozaču. '"Stop!" she shouted to=at the driver.'	crying	META	NP_nom [Agent] V QUOT [Theme] NP_dat [Recipient]			NEUTRAL	
**naricati 'lament, wail'**	Jedan od problema je i tipična filozofija mase koja samo nariče da nema novca, nema rezultata i nema gazde. 'One of the problems is the typical philosophy of the masses that just whines that they don't have money, results and boss=about having no money, no results, and no boss.'	crying	META	NP_nom [Agent] V da ('that')_CP [Theme]	NP_nom [Experiencer] V PP_nad+inst [Cause]	NP_nom [Experiencer] V PP_za ('for')+inst [Cause]	NEG	
**naricati 'lament, wail'**	Na tvom mjestu pokrio bih se po ušima i zavukao u mišju rupu, a ne naricao nad mirovinskim fondovima. 'If I were you, I'd cover my ears and crawl into a mouse hole, not whine over=about pension funds.'	crying	META	NP_nom [Agent] V PP_nad ('over')+inst [Theme]	NP_nom [Experiencer] V PP_nad+inst [Cause]	NP_nom [Experiencer] V PP_za ('for')+inst [Cause]	NEG	
**naricati 'lament, wail'**	Kad ga se opomene, onda nariče o patriotizmu Zvonka Makovića. 'When he is admonished, he whines about the patriotism of Zvonko Maković.'	crying	META	NP_nom [Agent] V PP_o ('about')+loc [Theme]	NP_nom [Experiencer] V PP_nad+inst [Cause]	NP_nom [Experiencer] V PP_za ('for')+inst [Cause]	NEG	
**naricati 'lament, wail'**	"Nemoj nas ostaviti" - nariču čobani. '"Don't leave us!" – the shepherds whine.'	crying	META	NP_nom [Agent] V QUOT [Theme]	NP_nom [Experiencer] V PP_nad+inst [Cause]	NP_nom [Experiencer] V PP_za ('for')+inst [Cause]	NEUTRAL	
**plakati 'cry'**	SAMI ste to odlučili, sada plačete kako je to nepravedno. 'YOU decided that yourselves, and now you're crying about how unfair it is.'	crying	META	NP_nom [Agent] V kako ('that')_CP [Theme]	NP_nom [Experiencer] V		NEUTRAL	
**zajecati 'sob'**	Laurice, Laurice - zajeca starica i pritegnu me drhtavim rukama k svojim obrazima. '"Laurice, Laurice" – the old woman sobs and pulls me close with her trembling hands to her cheeks.'	crying	META	NP_nom [Agent] V QUOT [Theme]			NEUTRAL	
**zaplakati 'cry'**	Mislite li da je to šala?", zaplače Magda, "to će biti moja smrt." '"Do you think this is a joke?" Magda cries, "This will be my death."'	crying	META	NP_nom [Agent] V QUOT [Theme]	NP_nom [Experiencer] V		NEUTRAL	
**drobiti 'crush, grind'**	...prvo je drobila da nema broj erste banke. '...first, she was crushing=rambling that she didn't have the Erste Bank number.'	deconstruction	META	NP_nom [Agent] V da ('that')_CP [Theme]	NP_nom [Agent] V NP_acc [Patient]		NEG	
**drobiti 'crush, grind'**	...i drobi bedastoce sto se tice godina. '...and she keeps crushing=rambling nonsense about age.'	deconstruction	META	NP_nom [Agent] V NP_acc [Theme]	NP_nom [Agent] V NP_acc [Patient]		NEG	
**drobiti 'crush, grind'**	...ma šta drobite gluposti o tome kako žena razara u takvim situacijama njegovu obitelj. '...what nonsense are you rambling about, saying how a woman destroys his family in such situations.'	deconstruction	META	NP_nom [Agent] V NP_acc [Theme]	NP_nom [Agent] V NP_acc [Patient]		NEG	
**drobiti 'crush, grind'**	Mozda drobim gluposti. 'Maybe I'm crushing=rambling nonsense.'	deconstruction	META	NP_nom [Agent] V NP_acc [Theme]	NP_nom [Agent] V NP_acc [Patient]		NEG	
**drobiti 'crush, grind'**	No umjesto racionalnog razmišljanja treba ovdje drobiti o kapitalizmu i socrealizmu. 'Instead of rational thinking, here we should crush=ramble about capitalism and socialist realism.'	deconstruction	META	NP_nom [Agent] V PP_o ('about')+loc [Theme]	NP_nom [Agent] V NP_acc [Patient]		NEG	
**drobiti 'crush, grind'**	A šta vi svi drobite protiv nje? 'Why are you all grinding=railing against her?'	deconstruction	META	NP_nom [Agent] V PP_protiv ('against')+gen [Theme]NP_acc [Theme]				

VERB	EXAMPLE	SOURCE DOMAIN	METAPHOR/METONYMY	VALENCY FRAME OF THE EXAMPLE	VALENCY FRAME IN THE SOURCE DOMAIN	VALENCY FRAME IN THE SOURCE DOMAIN	EMOTIONAL VALENCY	HIGHLIGHTED MAPPING
**izrešetati 'riddle with bullets'**	...a kad bih ga izrešetao o teoriji zavjera, o tajnim oružjima, imanentizaciji eshatona, o demonologiji i demoniacima, o crnoj ezoteriji, onak - ekspertno, opet vidiš da pojma nema. '...and if I were to riddle him with bullets=bombard him about=with conspiracy theories, secret weapons, the immanentization of the eschaton, demonology and demonics, black esotericism, you know – expertly, you'd still see that he has no clue.'	deconstruction	META	NP_nom [Agent] V NP_acc [Patient]/[Patient/Recipient] PP_o ('about')+loc [Theme]	NP_nom [Agent] V NP_acc [Patient]		NEG	
**mljeti 'grind'**	Dakle, nemojte mljet da se vozio "bahato brzo". 'So, don't grind that he was driving "recklessly fast"=ramble on about driving "recklessly fast."'	deconstruction	META	NP_nom [Agent] V da ('that')_CP [Theme]	NP_nom [Agent] V NP_acc [Patient]		NEG	
**mljeti 'grind'**	Opet Bozanić melje gluposti. 'Again, Bozanić is grinding=spouting nonsense.'	deconstruction	META	NP_nom [Agent] V NP_acc [Theme]	NP_nom [Agent] V NP_acc [Patient]		NEG	
**mljeti 'grind'**	...melje o nekakvim povijesnim falsifikatima. '...he's grinding=rambling about some historical forgeries.'	deconstruction	META	NP_nom [Agent] V PP_o ('about')+loc [Theme]	NP_nom [Agent] V NP_acc [Patient]		NEG	
**piliti 'saw'**	Uostalo zasto pilim da vozi brzo kad spavam. 'After all, why do I saw=nag that he drives fast =nag at him about driving fast.'	deconstruction	META	NP_nom [Agent] V da ('that')_CP [Theme]	NP_nom [Agent] V NP_acc [Patient]		NEG	
**piliti 'saw'**	Nema potrebe da stalno 'pilite' prijatelje s pričom o svojoj vezi. 'There's no need to constantly "saw"="nag" your friends with stories about your relationship.'	deconstruction	META	NP_nom [Agent] V NP_acc [Patient]/[Patient/Recipient] PP_s+inst [Instrument]	NP_nom [Agent] V NP_acc [Patient]		NEG	
**piliti 'saw'**	...koja je doslovno svima pilila o tome kako ju u školi krivo gledaju. '...who literally kept sawing=nagging everyone about how she's being looked at wrong in school.'	deconstruction	META	NP_nom [Agent] V PP_o ('about')+loc [Theme] NP_dat [Recipient]	NP_nom [Agent] V NP_acc [Patient]		NEG	
**piliti 'saw'**	...naši braniteljski vitezovi … koji su ga pilili za ćirilicu "… our defenders of the knights... who sawed=nagged him about using Cyrillic.'	deconstruction	META	NP_nom [Agent] V PP_za ('for')+acc [Theme] NP_acc [Recipient]/[Patient]	NP_nom [Agent] V NP_acc [Patient]		NEG	
**rešetati 'riddle with bullets'**	Kaže da su inženjeri strepili od susreta s Jobsom u dizalu, jer bi ih tada " rešetao " o tome što trenutno rade. 'He says that engineers dreaded meeting Jobs in the elevator because he would then riddle them with bullets="grill" them about what they were currently working on.'	deconstruction	META	NP_nom [Agent] V NP_acc [Patient]/[Patient/Recipient] PP_o ('about')+loc [Theme]	NP_nom [Agent] V NP_acc [Patient]		NEG	
**hihotati 'giggle'**	...koji su se zadovoljno nešto zezali i hihotali: - Za koga si glasao? '...who were happily joking around and giggling: "Who did you vote for?"'	laughing	META	NP_nom [Agent] V QUOT [Theme]	NP_nom [Agent] V		POSITIVE	
**hihotati 'giggle'**	Ženska djeca stanu hihotati: - A, a, Laurica je žena Ferkonjina. 'The girls start giggling: "Ah, ah, Laurica is Ferkonja's wife."'	laughing	META	NP_nom [Agent] V QUOT [Theme]	NP_nom [Agent] V		POSITIVE	
**hihotati 'giggle'**	Gladna sam, hahahaha - zahihotala je Marina onim nazalnim hihotajem. '"I'm hungry, hahahaha," Marina giggled with that nasal giggle.'	laughing	META	NP_nom [Agent] V QUOT [Theme]	NP_nom [Agent] V		POSITIVE	
**jodlati 'yodel'**	...dok su Hrvati-domoljubi, jodlali o pravednosti i uzvišenim idealima. '...while the patriotic Croats yodeled about justice and noble ideals.'	singing	META	NP_nom [Agent] V PP_o ('about')+loc [Theme]	NP_nom [Agent] V (NP_acc[Theme])		POSITIVE (IRONY)	cheerful, funny
**jebati 'fuck'**	I isto tako kako svi jebu po Kranjčaru, čovjek je fenomenalno igrao. '...And just like everyone else is fucking over=is saying, Kranjčar played phenomenally.	social interaction	META	NP_nom [Agent] V PP_po ('over')+loc [Theme]	NP_nom [Agent] V NP_acc [Patient]		NEG	agressive, repetitive
**brujati 'buzz'**	...američki mediji su tjednima brujali da se zapravo radi o PR potezu starlete. '...American media buzzed for weeks that it was actually a PR move by a starlet.'	sound emission	META	NP_nom [Agent] V da ('that')_CP [Theme]	NP_nom [Theme] V		NEUTRAL	loud (public), constant

VERB	EXAMPLE	SOURCE DOMAIN	METAPHOR/METONYMY	VALENCY FRAME OF THE EXAMPLE	VALENCY FRAME IN THE SOURCE DOMAIN	VALENCY FRAME IN THE SOURCE DOMAIN	EMOTIONAL VALENCY	HIGHLIGHTED MAPPING
**brujati 'buzz'**	Mediji su brujali o iznosima od 13 milijuna kuna.<br>'The media buzzed about the amounts of 13 million kuna.'	sound emission	META	NP_nom [Agent] V PP_o ('about')+loc [Theme]	NP_nom [Theme] V		NEUTRAL	loud (public), constant
**brujati 'buzz'**	Cijela Tuzla samo bruji: Vraća se Naser…<br>'The whole of Tuzla is buzzing: Naser is coming back.'	sound emission	META	NP_nom [Agent] V QUOT [Theme]	NP_nom [Theme] V		NEUTRAL	loud (public), constant
**bubnuti 'slam'**	...proizvođač ionak nema pojma o tome pa je iz fore bubnuo da bi perajica trebala biti 23,5.<br>'...the manufacturer, who has no clue about it anyway, just slammed=threw out that the fin should be 23.5 as a joke.'	sound emission	META	NP_nom [Agent] V da ('that')_CP [Theme]	NP_nom [Agent] V NP_inst [Instrument]	NP_nom [Instrument] V	NEG	stupid (sudden loud sound)?
**bubnuti 'slam'**	...da nesto bubnu o cestarinama.<br>'...to slam=blurt out something about tolls.'	sound emission	META	NP_nom [Agent] V NP_acc [Theme]	NP_nom [Agent] V NP_inst [Instrument]	NP_nom [Instrument] V	NEG	stupid (sudden loud sound)?
**bubnuti 'slam'**	Govore u neprikladno vrijeme i često nepromišljeno "bubnu" odgovore.<br>'They speak at inappropriate times and often thoughtlessly slam=blurt out answers.'	sound emission	META	NP_nom [Agent] V NP_acc [Theme]	NP_nom [Agent] V NP_inst [Instrument]	NP_nom [Instrument] V	NEG	stupid (sudden loud sound)?
**bubnuti 'slam'**	Ono što je Tolja bubnuo za Rozgu više je dosadno nego uvredljivo.<br>'What Tolja slammed=blurted for=about Rozga is more boring than offensive.'	sound emission	META	NP_nom [Agent] V PP_za ('for')+acc [Theme] NP_acc [Theme]				
**bubnuti 'slam'**	Smijući se i dalje, bubnem: - Okladu nije osvojilo niti jedno od vas.<br>'Still laughing, I slammed=blurt out: "Neither of you won the bet."'	sound emission	META	NP_nom [Agent] V QUOT [Theme]	NP_nom [Agent] V NP_inst [Instrument]	NP_nom [Instrument] V	NEG	stupid (sudden loud sound)?
**cinkati 'clang'**	Čim ide cinkat vlastitu mater i ćaću…<br>'As soon as he goes to clang his own mother and father=snitch on his own mother and father…'	sound emission	META	NP_nom [Agent] V NP_acc [Theme]	NP_nom [Theme] V		NEG	snitch (sudden short sound)
**cinkati 'clang'**	Obećao sam da vas ja neću cinkati službama.<br>'I promised I wouldn't clang you=snitch on you to the authorities.'	sound emission	META	NP_nom [Agent] V NP_acc [Theme] NP_dat [Recipient]	NP_nom [Theme] V		NEG	snitch (sudden short sound)
**cinkati 'clang'**	Cinkali smo mu što Šarić drži na polici.<br>'We clanged him what Šarić keeps on the shelf=snitched to him about what Šarić keeps on the shelf.'	sound emission	META	NP_nom [Agent] V što ('what')_CP [Theme] PP_dat [Recipient]	NP_nom [Theme] V		NEG	snitch (sudden short sound)
**gruvati 'pound'**	Iz tog razloga i tvrdim da ipak ponešto znam, da ne gruvam napamet pretpostavke.<br>'For that reason, I claim that I do know something, and that I'm not just blindly pounding=making assumptions.'	sound emission	META	NP_nom [Agent] V NP_acc [Theme]	NP_nom [Theme] V		NEG	loud, incomprehensible
**gruvati 'pound'**	Tko plaća ovu babu da mi je znati, samo gruva gluposti…<br>'Who's paying this old woman, I'd like to know, she's just pounding=spouting nonsense…'	sound emission	META	NP_nom [Agent] V NP_acc [Theme]	NP_nom [Theme] V		NEG	loud, incomprehensible
**jeknuti 'echoe'**	"Hajdemo, djevojko" - jeknu joj iznenada u uho momački glas.<br>'"Come on, girl," a masculine voice suddenly echoed in her ear.'	sound emission	META	NP_nom [Agent_METO] V QUOT [Theme] NP_dat [Recipient]	NP_nom [Theme] V		NEG	loud, unpleasant
**klepetati 'clatter'**	Latinu nije dosta 30 000 kn za 1 tjedan neg klepeće laži o privatizaciji…<br>'Latin doesn't find 30,000 kuna enough for one week, but instead he clatters=blabbers lies about privatization…'	sound emission	META	NP_nom [Agent] V NP_acc [Theme]	NP_nom [Agent] V (NP_inst [Instrument])	NP_nom [Theme] V	NEG	repetitive, irritating
**klepetati 'clatter'**	Bitno je prodavati novine i klepetati o laznom suosjecanju s obitelji.<br>'It is important to sell newspapers and clatter=chatter about fake sympathy with the family'	sound emission	META	NP_nom [Agent] V PP_o ('about')+loc [Theme]	NP_nom [Agent] V (NP_inst [Instrument])	NP_nom [Theme] V	NEG	repetitive, irritating
**lupati 'bang'**	...bolje da nešto naučim nego lupam gluposti o stvarima čiju sam površinu samo zagrebala.<br>'...it's better if I learn something rather than bang=blabber nonsense about things I've only scratched the surface of.'	sound emission	META	NP_nom [Agent] V NP_acc [Theme] PP_o ('about')+loc [Theme]	NP_nom [Agent] V (NP_inst [Instrument])	NP_nom [Theme] V	NEG	stupid? (sudden loud sound)?

VERB	EXAMPLE	SOURCE DOMAIN	METAPHOR/METONYMY	VALENCY FRAME OF THE EXAMPLE	VALENCY FRAME IN THE SOURCE DOMAIN	VALENCY FRAME IN THE SOURCE DOMAIN	EMOTIONAL VALENCY	HIGHLIGHTED MAPPING
**lupati 'bang'**	Danas si se registrirao i lupaš neistine.<br>'Today you registered and you're banging=spouting lies.'	sound emission	META	NP_nom [Agent] V NP_acc [Theme]	NP_nom [Agent] V (NP_inst [Instrument])	NP_nom [Theme] V	NEG	lying (sudden loud sound)?
**lupati 'bang'**	Nemoj lupati gluposti da znaš kome sam sličan.<br>'Don't bang=spout nonsense that you know=about knowing who I resemble.'	sound emission	META	NP_nom [Agent] V NP_acc [Theme] CP_da [Theme]	NP_nom [Agent] V (NP_inst [Instrument])	NP_nom [Theme] V	NEG	stupid? (sudden loud sound)?
**lupati 'bang'**	...nepestano lupali gluposti o žminju.<br>'...they kept banging=spouting nonsense about Žminj.'	sound emission	META	NP_nom [Agent] V NP_acc [Theme] PP_o ('about')+loc [Theme]	NP_nom [Agent] V (NP_inst [Instrument])	NP_nom [Theme] V	NEG	stupid? (sudden loud sound)?
**lupiti 'bang'**	Bude ti neugodno kad lupiš glupost.<br>'You'll feel uncomfortable when you bang nonsense=say something stupid.'	sound emission	META	NP_nom [Agent] V NP_acc [Theme]	NP_nom [Agent] V (NP_inst [Instrument]) / NP_nom [Theme] V	NP_nom [Theme] V	NEG	stupid (sudden loud sound)?
**mrmoriti 'gurgle'**	Kraj nje bi zastali u prolazu stariji ljudi, mrmorili svoje molitve i ubacili po koji novčić.<br>'Older people would stop by her in the passage, gurgle=murmur their prayers, and drop a coin or two.'	sound emission	META	NP_nom [Agent] V NP_acc [Theme]	NP_nom [Theme] V		NEUTRAL	low voice, hard to understand
**mrmoriti 'gurgle'**	Teta kraj stolića je zlamenovala se i mrmorila je: Ujmeoca, isina, iduva	sound emission	META	NP_nom [Agent] V QUOT [Theme]	NP_nom [Theme] V		NEUTRAL	low voice, hard to understand
**otkucati 'strike'**	Ne zna da ju je netko otkucao policiji.<br>'She doesn't know that someone struck=snitched on her to the police.'	sound emission	META	NP_nom [Agent] V NP_acc [Theme] NP_dat [Recipient]	NP_nom [Theme] V		NEG	snitch (sudden short sound)
**pištati 'whistle, shriek'**	"Što je ovo, nesrećo stara?" - pišti snaha.<br>'"What is this, you old misery?" – the daughter-in-law screeches.'	sound emission	META	NP_nom [Agent] V QUOT [Theme]	NP_nom [Theme] V		NEG	high pitch, agressive
**praskati 'crack, pop, snap'**	Pa izjasni se onda, boga mu - praskam, jer više ne mogu izdržati: njen bespomoćni izgled, koji mi je nekada budio zaštitnički nagon prema njenoj krhkosti, sada ima suprotni učinak i budi agresiju.<br>'Well, speak up then, for God's sake – I snap, because I can't take it anymore: her helpless appearance, which once awakened a protective instinct due to her fragility, now has the opposite effect and awakens aggression in me.'	sound emission	META	NP_nom [Agent] V QUOT [Theme]	NP_nom [Theme] V		NEG	angry (explosive sound)
**prasnuti 'crack, pop, snap'**	Prasnuo glupost pa jos ostao i ziv?<br>'He snapped out some nonsense and still lived to tell the tale?'	sound emission	META	NP_nom [Agent] V NP_acc [Theme]	NP_nom [Theme] V		NEG	stupid (sudden loud sound)?
**prošištati 'whistle, shriek'**	Isuse - prošištala je tiho staviviši ruku na suprugovu glavu.<br>'Jesus – she shrieked softly, placing her hand on her husband's head.'	sound emission	META	NP_nom [Agent] V QUOT [Theme]	NP_nom [Theme] V		NEUTRAL	low voice, hard to understand
**prozviždati 'whistle'**	Mnogi koji su prozviždali o velikim kriminalnim radnjama ostali su bez posla.<br>'Many who whistled about=blew the whistle on major criminal activities lost their jobs.'	sound emission	META	NP_nom [Agent] V PP_o ('about')+loc [Theme]			NEUTRAL	make public, loud sound
**prozviždati 'whistle'**	...rekla je kako joj je čudno da je Mesić " prozviždao " o Ini tek sada na kraju svog mandata.<br>'...she said it was strange that Mesić "whistled" about INA only now, at the end of his term.'	sound emission	META	NP_nom [Agent] V PP_o ('about')+loc [Theme]			NEUTRAL	make public, loud sound
**romoriti 'murmur'**	Zli jezici romore da je Konstantin bio rimski, a ne srpski car.<br>'Evil tongues murmur that Constantine was a Roman emperor, not a Serbian one.'	sound emission	META	NP_nom [Agent] V da ('that')_CP [Theme]	NP_nom [Theme] V		NEUTRAL	low voice, hard to hear
**ruknuti 'slam'**	Ej, vidjet ćemo - ruknu Mecena i pokroči sobama poput psa koji njuši lovinu.<br>'"Hey, we'll see," Mecena slammed, prowling through the rooms like a dog sniffing for prey.'	sound emission	META	NP_nom [Agent] V QUOT [Theme]	NP_nom [Theme] V		NEG	agressive, angry (explosive sound)
**škripati 'creak'**	Vidite da je pijan - škripaše Bradić blijed, grozeći se objema šakama.<br>'"You can see he's drunk," Bradić creaked, pale, recoiling with both fists clenched.'	sound emission	META/METO	NP_nom [Agent] V QUOT [Theme]	NP_nom [Agent] V (NP_inst [Instrument])	NP_nom [Theme] V	NEG	agressive, angry

VERB	EXAMPLE	SOURCE DOMAIN	METAPHOR/METONYMY	VALENCY FRAME OF THE EXAMPLE	VALENCY FRAME IN THE SOURCE DOMAIN	VALENCY FRAME IN THE SOURCE DOMAIN	EMOTIONAL VALENCY	HIGHLIGHTED MAPPING
**škripnuti 'creak'**	To mi je za žgaravicu - škripnuo sam pospanim, hrapavim zvukom i njegov univerzum obasjalo je Iskonsko Svjetlo. '"This is giving me heartburn," I creaked with a sleepy, hoarse sound, and his universe was illuminated by the Primordial Light.'	sound emission	META	NP_nom [Agent] V QUOT [Theme]	NP_nom [Agent] V (NP_inst [Instrument])	NP_nom [Theme] V	NEUTRAL	low unrefined voice
**šuškati 'rustle'**	Okupljeni u povjerljivoj grupici, ljudi su šuškali da se Titovo stanje pogoršalo. 'Gathered in a confidential group, people rustled=whispered that Tito's condition had worsened.'	sound emission	META	NP_nom [Agent] V da ('that')_CP [Theme]	NP_nom [Agent] V (NP_inst [Instrument])	NP_nom [Theme] V	NEUTRAL	low voice, hard to hear
**šuškati 'rustle'**	Govorio je o tome zamumuljeno, malo je i pleo jezikom, ali se moglo razabrati da se u mjestu mnogo govori o Terinu slučaju, da ljudi već šuškaju o pravom čudu (čemu pogoduje i Terin bogobojazni život), i da su već spremni na tko zna kakve zaključke i akcije. 'He spoke about it muffled, a little tongue-tied, but it could be understood that the case of Terin was being widely discussed in the town, that people were already rustling=whispering about a real miracle (which is supported by Terin's devout life), and that they were ready for who knows what conclusions and actions.'	sound emission	META	NP_nom [Agent] V PP_o ('about')+loc [Theme]	NP_nom [Agent] V (NP_inst [Instrument])	NP_nom [Theme] V	NEUTRAL	low voice, hard to understand
**tandrkati 'rattle'**	Nastavite tandrkati o YU i CRO-krivici i kronologiji, molim lijepo? 'Please, continue rattling=chattering about YU and CRO guilt and chronology.'	sound emission	META	NP_nom [Agent] V PP_o ('about')+loc [Theme]	NP_nom [Theme] V		NEG	loud, irritating
**tandrkati 'rattle'**	...barem ovaj puta nisu tandrkali o 2 zaleđa kada lopta ide u korner zastavicu. '...at least this time they weren't rattling=chattering about the two back passes when the ball goes to the corner flag.'	sound emission	META	NP_nom [Agent] V PP_o ('about')+loc [Theme]	NP_nom [Theme] V		NEG	irritating, unpleasant
**tresnuti 'slam'**	...čija je voditeljica tresnula da više nitko ne BACA ruku u vatru ni za čije poštenje... '...whose host slammed that no one throws their hand into the fire for anyone's honesty anymore...'	sound emission	META	NP_nom [Agent] V da ('that')_CP [Theme]	NP_nom [Agent] V (NP_inst [Instrument])	NP_nom [Theme] V	NEG	stupid (sudden loud sound)?
**trubiti 'honk'**	Većina trubi kako smo sinoć igrali grozno. 'Most people are honking how badly=about how badly we played last night.	sound emission	META	NP_nom [Agent] V kako ('that')_CP [Theme]	NP_nom [Agent] V (NP_dat [Recipient])	NP_nom [Theme] V	NEG	loud, irritating, repetitive
**trubiti 'honk'**	Ljubomora, pa sada trubi gluposti. 'Jealousy, and now it's honking=blabbering nonsense.'	sound emission	META	NP_nom [Agent] V NP_acc [Theme]	NP_nom [Agent] V (NP_dat [Recipient])	NP_nom [Theme] V	NEG	loud, stupid
**trubiti 'honk'**	...vi hrvatski mediji bi trubili o velikom povratku golgeterske forme. '...you Croatian media would honk=blabber on about the great return of goal-scoring form.'	sound emission	META	NP_nom [Agent] V PP_o ('about')+loc [Theme]	NP_nom [Agent] V (NP_dat [Recipient])	NP_nom [Theme] V	NEG	make public, loud sound
**tuliti 'blare, wail'**	...i dalje ne priznaju svoju krivnju već uporno tule da su oni bili napadnuti. '...they still don't acknowledge their guilt and stubbornly blare that they were the ones attacked.'	sound emission	META	NP_nom [Agent] V da ('that')_CP [Theme]	NP_nom [Theme] V		NEG	loud, irritating, persistent
**tuliti 'blare, wail'**	...a zovem Brazil i tulim frendovima da osjecam saudade i da ne mogu izdrzat... '...and I call Brazil and blare to my friends that I feel saudade and that I can't take it anymore...'	sound emission	META	NP_nom [Agent] V da ('that')_CP [Theme] NP_dat [Recipient]	NP_nom [Theme] V		NEG	crying, loud, persistent
**tuliti 'blare, wail'**	Svaka čast, već neko vrijeme tulim istu stvar i drago mi je vidjeti da ima još ljudi koji tako razmišljaju. 'Kudos, I've been blaring the same thing for a while, and I'm glad to see there are others who think the same way.'	sound emission	META	NP_nom [Agent] V NP_acc [Theme]	NP_nom [Theme] V		NEUTRAL/NEG	persistent
**tuliti 'blare, wail'**	Pa ti tuli o primitivcima koliko hoćeš. 'Pa ti tuli o primitivcima koliko hoćeš.'	sound emission	META	NP_nom [Agent] V PP_o ('about')+loc [Theme]	NP_nom [Theme] V		NEG	loud, irritating, persistent

VERB	EXAMPLE	SOURCE DOMAIN	METAPHOR/METONYMY	VALENCY FRAME OF THE EXAMPLE	VALENCY FRAME IN THE SOURCE DOMAIN	VALENCY FRAME IN THE SOURCE DOMAIN	EMOTIONAL VALENCY	HIGHLIGHTED MAPPING
**tuliti 'blare, wail'**	"Hoćete li Ivana Forčića za podbana?" "Hoćemo", tulila skupština.<br>'"Do you want Ivan Forčić as deputy?" "Yes, we do", the assembly wailed.'	sound emission	META	NP_nom [Agent] V QUOT [Theme]	NP_nom [Theme] V		NEUTRAL/NEG	persistent, loud
**zaječati 'wail'**	Ne govori - zaječala je.<br>'"Don't speak," she wailed.'	sound emission	META	NP_nom [Agent] V QUOT [Theme]	NP_nom [Theme] V		NEUTRAL	low, crying voice
**zapištati 'whistle, shriek''**	Evo šta me košta - bijesno zapišti Jusuf.<br>'"Here's what it costs me," Jusuf whistles=squeals angrily.'	sound emission	META	NP_nom [Agent] V QUOT [Theme]	NP_nom [Theme] V		NEG	angry, pressurized container sound, danger
**zatuliti 'blare, wail'**	...povremeno bi pokoji country muzičar zatulio o odnosu čovjeka i psa ili čovjeka i pištolja.<br>'...occasionally, a country musician would blare about the relationship between a man and a dog or a man and a gun.'	sound emission	META	NP_nom [Agent] V PP_o ('about')+loc [Theme]	NP_nom [Theme] V		NEG	crying sound, unpleasant
**zatuliti 'blare, wail'**	Vidiš ti vraga, zatuli gotovo plačno mali.<br>'"You see, damn it," the little one blared, almost tearfully.'	sound emission	META	NP_nom [Agent] V QUOT [Theme]	NP_nom [Theme] V		NEG	crying, loud, persistent
**zvocati 'clang'**	Hoces li mu zvocati da si ti planirala nesto drugo?<br>'Are you going to clang=nag to him that you had planned something else?'	sound emission	META	NP_nom [Agent] V da ('that')_CP [Theme] NP_dat [Recipient]			NEG	irritating, repetitive
**zvocati 'clang'**	Samo sjediš i ništa ne radiš - zvocala mu je.<br>'"You're just sitting there doing nothing," she clanged=nagged at him.'	sound emission	META	NP_nom [Agent] V QUOT [Theme] NP_dat [Recipient]			NEG	irritating, repetitive
**zvoncati 'clang'**	No, građani stalno zvoncaju da žele "jeftinu državu...<br>'Well, the citizens keep clanging=nagging that they want a "cheap state..."'	sound emission	META	NP_nom [Agent] V da ('that')_CP [Theme]			NEG	irritating, repetitive
**zvoncati 'clang'**	Npr. viši znanstveni savjetnik - docent (znam jednu) će brzo zvoncati kako želi i pripadno zvanje izvanrednog profesora...<br>'For example, a senior research advisor – an assistant professor (I know one) will quickly start clanging=nagging about wanting the corresponding title of associate professor...	sound emission	META	NP_nom [Agent] V kako ('that')_CP [Theme]			NEG	irritating, repetitive
**zvrndati 'twirl'**	Zvrndaju da moramo imat kutije po EU propisima ko da ćemo sutra ući u uniju.<br>'They keep twirling=whining that we need to have boxes according to EU regulations, as if we're going to join the union tomorrow.'	sound emission	META	NP_nom [Agent] V da ('that')_CP [Theme]			NEG	irritating, repetitive
**zvrndati 'twirl'**	...ne zvrndajte mu pretjerano jer se život u kućici ne zbiva onako kako ste si zamislili.<br>'...don't twirl to him=whine at him too much, because life in the little house isn't turning out the way you imagined.'	sound emission	META	NP_nom [Agent] V NP_dat [Recipient]			NEG	irritating, repetitive
**zvrndati 'twirl'**	...informacija je TOČNA i što sad opet zvrndaš o mojoj metodologiji...<br>'...the information is ACCURATE, so why are you whining again about my methodology...'	sound emission	META	NP_nom [Agent] V PP_o ('about')+loc [Theme]			NEG	irritating, repetitive
**zvrndati 'twirl'**	Ako zvrndamo okolo za kriminalce da su kriminalci, a nije im to dokazano sami postajemo kriminalcima.<br>'If we twirl for criminals that are criminals=whine around calling people criminals without it being proven, we ourselves become criminals.'	sound emission	META	NP_nom [Agent] V PP_za ('for')+acc [Theme] da ('that')_CP [Theme]			NEG	irritating, repetitive
**žamoriti 'ripple'**	...začarava ih ulomcima rasprava s katedra svjetske mudrosti, žamori im o afričkim dijamantima i zlatu.<br>'...he enchants them with fragments of discussions from the world's wisdom podium, rippling=murmuring about African diamonds and gold.'	sound emission	META	NP_nom [Agent] V PP_o ('about')+loc [Theme] NP_dat [Recipient]	NP_nom [Theme] V		POSITIVE	low, pleasant sound

VERB	EXAMPLE	SOURCE DOMAIN	METAPHOR/METONYMY	VALENCY FRAME OF THE EXAMPLE	VALENCY FRAME IN THE SOURCE DOMAIN	VALENCY FRAME IN THE SOURCE DOMAIN	EMOTIONAL VALENCY	HIGHLIGHTED MAPPING
**grmjeti 'thunder'**	A da nije pobjede, vjerojatno bi iz Gradskog vrta grmjeli na Marija Strahonju.<br>'If it weren't for the victory, they'd probably be thundering at Mario Strahonja from the City Garden.'	sound emission/weather	META	NP_nom [Agent] V PP_na ('at')+acc [Patient]/[Recipient]	NP_nom [Theme] V		NEG	very loud, agressive
**grmjeti 'thunder'**	...najlakše je onda pravedno grmjeti protiv majki ubojica.<br>'...then it is easiest to righteously thunder against women who abort.'	sound emission/weather	META	NP_nom [Agent] V PP_protiv ('against')+gen [Theme]	NP_nom [Theme] V		NEUTRAL	loud, public
**grmjeti 'thunder'**	Kada je svršio, vratila se duša u načelnika koji je sada prvi počeo iz svega glasa grmjeti : - Živjeli, živjeli - a za njim svi drugi.<br>'When he finished, the soul returned to the chief, who now was the first to start thundering at the top of his lungs: "Cheers, cheers!" and everyone else followed.'	sound emission/weather	META	NP_nom [Agent] V QUOT [Theme]	NP_nom [Theme] V		NEUTRAL	very loud
**zagrmjeti 'thunder'**	Kad je jedan od njih zagrmio da i njih slikam.<br>'Kad je jedan od njih zagrmio da i njih slikam'	sound emission/weather	META	NP_nom [Agent] V da ('that')_CP [Theme]	NP_nom [Theme] V		NEG	very loud, agressive
**zagrmjeti 'thunder'**	Izludjet ću s tobom - zagrmio je Robert.<br>'"I'm going to go crazy with you," Robert thundered.'	sound emission/weather	META	NP_nom [Agent] V QUOT [Theme]	NP_nom [Theme] V		NEG	very loud, agressive
**dobaciti 'throw, toss'**	A slušao sam jednom Cesarića kako je dobacio svomu kolegi romanopiscu da jedna njegova pjesma sadrži više poezije nego svi romani.<br>'Once, I heard Cesarić threw=quip to his colleague, a novelist, that one of his (Cesarić's) poems contained more poetry than all of his colleague's novels combined.'	throwing	META	NP_nom [Agent] V da ('that')_CP [Theme] NP_dat [Recipient]	NP_nom [Agent] V NP_acc [Theme] NP_dat [Recipient]		NEUTRAL	
**dobaciti 'throw, toss'**	Na takve optužbe Čeljuska mu je dobacio kako on njegovu ženu ne bi dotaknuo ni štapom.<br>'In response to such accusations, Čeljuska threw=retorted (to him) that he wouldn't touch his wife with a stick.'	throwing	META	NP_nom [Agent] V kako ('that')_CP [Theme] NP_dat [Recipient]	NP_nom [Agent] V NP_acc [Theme] NP_dat [Recipient]		NEUTRAL	
**dobaciti 'throw, toss'**	Elio mu je dobacio neka se potpiše.<br>'Elio tossed him to sign it.'	throwing	META	NP_nom [Agent] V neka ('to')_CP [Theme] NP_dat [Recipient]	NP_nom [Agent] V NP_acc [Theme] NP_dat [Recipient]		NEUTRAL	
**dobaciti 'throw, toss'**	Nije imao srca biti zajedljiv pa dobaciti nešto o asfaltu.<br>'He didn't have the heart to be sarcastic and toss in something about the asphalt.'	throwing	META	NP_nom [Agent] V NP_acc [Theme]	NP_nom [Agent] V NP_acc [Theme] NP_dat [Recipient]		NEUTRAL	
**dobaciti 'throw, toss'**	Nakon utakmice u prolazu je novinarima dobacio : "Puklo je, gotov sam."<br>'After the match, as he was passing by, he threw out=called out to the reporters: 'It's snapped, I'm done.'	throwing	META	NP_nom [Agent] V QUOT [Theme] NP_dat [Recipient]	NP_nom [Agent] V NP_acc [Theme] NP_dat [Recipient]		NEUTRAL	
**nabacati 'throw around, scatter'**	Sigurno joj je neko nabacao kako mučim Laticu.<br>'Someone probably tossed her that I'm tormenting Latica=the idea that I'm tormenting Latica.'	throwing	META	NP_nom [Agent] V kako ('that')_CP [Theme] NP_dat [Recipient]	NP_nom [Agent] V NP_acc [Theme] NP_dat [Recipient]	NP_nom [Agent] V NP_acc [Theme] AdvP [Goal]	NEUTRAL	
**nabaciti 'throw around, scatter'**	Ivan onako izdaleka nabaci, da bi morao novu kuću načiniti.<br>'Ivan casually threw=mentions from a distance that he would have to build a new house.Sigurno joj je neko nabacao kako mučim Laticu.<br>'Someone probably tossed her that I'm tormenting Latica=the idea that I'm tormenting Latica.'	throwing	META	NP_nom [Agent] V da ('that')_CP [Theme]	NP_nom [Agent] V NP_acc [Theme] NP_dat [Recipient]	NP_nom [Agent] V NP_acc [Theme] AdvP [Goal]	NEUTRAL	
**nabaciti 'throw around, scatter'**	Jedino, kada bi se zavadili, onda bi mu nabacili da je ciganin.<br>'Only, when they fought, they would throw at him that he was a gypsy.'	throwing	META	NP_nom [Agent] V da ('that')_CP [Theme] NP_dat [Recipient]	NP_nom [Agent] V NP_acc [Theme] NP_dat [Recipient]	NP_nom [Agent] V NP_acc [Theme] AdvP [Goal]	NEUTRAL	

VERB	EXAMPLE	SOURCE DOMAIN	METAPHOR/METONYMY	VALENCY FRAME OF THE EXAMPLE	VALENCY FRAME IN THE SOURCE DOMAIN	VALENCY FRAME IN THE SOURCE DOMAIN	EMOTIONAL VALENCY	HIGHLIGHTED MAPPING
**nabaciti 'throw around, scatter'**	Kad su se sreli, Krsto Brodnjak ga je ljubazno pozdravio i nabacio nešto o ulju i poslu. 'When they met, Krsto Brodnjak greeted him politely and casually threw=mentioned something about oil and business.'	throwing	META	NP_nom [Agent] V NP_acc [Theme]	NP_nom [Agent] V NP_acc [Theme] NP_dat [Recipient]	NP_nom [Agent] V NP_acc [Theme] AdvP [Goal]	NEUTRAL	
**nabaciti 'throw around, scatter'**	Pitanja, međutim, što ih je u svome »sporu« nabacio Nazor, ne tiču se toliko umjetničke strane njihova rada, koliko osnovnih pitanja života. 'The questions, however, that Nazor casually threw into his "dispute," are not so much related to the artistic side of their work, but rather to fundamental questions of life.'	throwing	META	NP_nom [Agent] V NP_acc [Theme]	NP_nom [Agent] V NP_acc [Theme] NP_dat [Recipient]	NP_nom [Agent] V NP_acc [Theme] AdvP [Goal]	NEUTRAL	
**nabaciti 'throw around, scatter'**	Kad ju je Hanibal (...) prvi put vidio kano gospodju, bilo je oboje tako već umireno, da je on mogao nabaciti joj nekoliko običnih komplimenata. 'When Hannibal first saw her, they were both so calm that he was able to throw= give her a few ordinary compliments.'	throwing	META	NP_nom [Agent] V NP_acc [Theme] NP_dat [Recipient]	NP_nom [Agent] V NP_acc [Theme] NP_dat [Recipient]	NP_nom [Agent] V NP_acc [Theme] AdvP [Goal]	NEUTRAL	
**nabaciti 'throw around, scatter'**	Kad me nadje slučajno samu, dršćuć vas zasopljen mi nabaci o ljubavi i malo ne uteče od stida. 'When he finds me alone by chance, trembling and out of breath, he casually throws in (to me) something about love and almost runs away from embarrassment.'	throwing	META	NP_nom [Agent] V PP_o ('about')+loc [Theme] NP_dat [Recipient]	NP_nom [Agent] V NP_acc [Theme] NP_dat [Recipient]	NP_nom [Agent] V NP_acc [Theme] AdvP [Goal]	NEUTRAL	
**nabaciti 'throw around, scatter'**	"Oštra jezika u tebe, udovice" – nabacila je suhonjava stara zaova, usidjelica. '"Sharp-tongued at you, widow," the dry, old sister-in-law, the spinster, threw=remarked.'	throwing	META	NP_nom [Agent] V QUOT [Theme]	NP_nom [Agent] V NP_acc [Theme] NP_dat [Recipient]	NP_nom [Agent] V NP_acc [Theme] AdvP [Goal]	NEUTRAL	
**nabacivati 'throw around continuously'**	Svi su nas čudno gledali i nabacivali da bih tokom trudnoće morala ipak jesti meso. 'Everyone looked at us strangely and threw in=suggested that I should still eat meat during my pregnancy.'	throwing	META	NP_nom [Agent] V da ('that')_CP [Theme]	NP_nom [Agent] V NP_acc [Theme] NP_dat [Recipient]	NP_nom [Agent] V NP_acc [Theme] AdvP [Goal]	NEG	
**nabacivati 'throw around continuously'**	Svi su mi nabacivali da ću teško ostat trudna zbog mršavosti! 'Everyone was throwing to me=kept telling me that I'd have trouble getting pregnant because of my thinness!'	throwing	META	NP_nom [Agent] V da ('that')_CP [Theme] NP_dat [Recipient]	NP_nom [Agent] V NP_acc [Theme] NP_dat [Recipient]	NP_nom [Agent] V NP_acc [Theme] AdvP [Goal]	NEG	
**nabacivati 'throw around continuously'**	Metodički i taktično voditelj navodi djecu da nabacuju ideje za film. 'Methodically and tactfully, the host guides the children to throw out ideas for the film.'	throwing	META	NP_nom [Agent] V NP_acc [Theme]	NP_nom [Agent] V NP_acc [Theme] NP_dat [Recipient]	NP_nom [Agent] V NP_acc [Theme] AdvP [Goal]	NEUTRAL	
**nabacivati 'throw around continuously'**	Dečko mi nešto nabacuje jer mi je rođendan blizu… 'My boyfriend is throwing=hinting something my way because my birthday is coming up…'	throwing	META	NP_nom [Agent] V NP_acc [Theme] NP_dat [Recipient]	NP_nom [Agent] V NP_acc [Theme] NP_dat [Recipient]	NP_nom [Agent] V NP_acc [Theme] AdvP [Goal]	NEUTRAL	
**nabacivati 'throw around continuously'**	Nije ona nikoga vriedjala, nije nikomu nabacivala: Gle, ja sam bolja od tebe. 'She didn't insult anyone, she didn't throw out to anyone: "Look, I'm better than you."'	throwing	META	NP_nom [Agent] V QOUT [Theme] NP_dat [Recipient]	NP_nom [Agent] V NP_acc [Theme] NP_dat [Recipient]	NP_nom [Agent] V NP_acc [Theme] AdvP [Goal]	NEG	
**poklopiti 'cover'**	"Ajde muči, ti si svoje proša", poklopi ga žena Mira Paladina, onako kako žene poklapaju muževe nakon pedeset godina braka. '"Come on, shut up, you've had your turn," Mira Paladina covers him= shuts him down, the way wives shut their husbands after fifty years of marriage.'	throwing	META	NP_nom [Agent] V QUOT [Theme] NP_acc [Patient]/[Patient/Recipient]	NP_nom [Agent] V NP_acc [Theme] NP_inst [Instrument]		NEG	
**spustiti 'put down'**	Ovaj (prilično loš) pokušaj sindikalista da 'spuste' premijeru Milanoviću mogao im se obiti o glavu. 'This (rather poor) attempt by the unionists to 'put down' to Prime Minister Milanović=shut up Prime Minister Milanović could backfire on them.'	throwing	META	NP_nom [Agent] V NP_dat [Recipient]	NP_nom [Agent] V NP_acc [Theme]		NEG	
