# Supplementary material for: Bleating, growling, barking, and spitting: Metaphorical extensions and valency patterns of verbs of speaking
Source: PLoS One. 2025 Jun 10;20(6):e0325807. doi: 10.1371/journal.pone.0325807 (PMC12151387; doi:10.1371/journal.pone.0325807)
Supplement: S3 File — (PDF) [file pone.0325807.s003.pdf]

Overview of valency patterns

**Sem. Role**	**Syntactic phrase type**	***govoriti* 'speak'**	***pričati* 'talk'**	***reći* 'say, tell'**	***kazati* 'say, tell'**	**sounds made by animals**	**sound emission**	**bodily processes**	**deconstruction**	**throwing**	**crying**	**breathing**
**Theme**	**QUOT**	+	+	+	+	+	+			+	+	+
	**Clausal complement**	+	+	+	+	+	+	+	+	+	+	+
	**NP in ACC (narrative-related expressions)**	+	+	+	+	+	+	+	+	+	NA	NA
	**PP *za* 'for, about' + ACC (contextualization)**	+	+	+	+		NA (*zvrndati* 'buzz')	context. not needed	*piliti* (stimulus)<br>*režati* (beneficiary)	NA	NA	NA
	**PP *o* 'about' + LOC**	+	+	(context.)	(context.)	+	+	+	+	+	+	NA
	**PP *protiv* 'against' + GEN**	+	+	(context.)	(context.)	+	NA (*grmjeti* 'thunder')	+	context. not needed	NA	NA	NA
	**PP *na* + ACC**	NA	NA	(context.)	(context.)	NA (*kokodaknuti* 'cluck')	NA	-	-	NA	NA	NA
	**PP *za* + INST**	NA	NA	NA	NA	NA (*zakokodakati* 'cluck')	-	-	-	NA	+ (cause)	NA
	**PP *nad* 'over' + INST**	NA	NA	NA	NA	-	NA	-	-	NA	+ (cause)	NA
**Recipient**	**NP in DAT**	+	+	+	+	+	+	+	+	+		+
	**PP *na* 'at' + ACC**	NA	NA	NA	NA	+ (patient/experiencer)	NA (*grmjeti* 'thunder')		-	NA	NA	NA
	**PP *po* 'on' + LOC**	NA	NA	NA	NA	NA	NA	+	NA	NA	NA	NA
	**NP in ACC**	NA	NA	NA	NA	NA	NA	*pljuvati* 'spit', *posrati* 'shit', *drkati* 'jerk'	+	NA	NA	NA
